# Regorafenib inhibits epithelial-mesenchymal transition and suppresses cholangiocarcinoma metastasis via YAP1-AREG axis

**DOI:** 10.1038/s41419-022-04816-7

**Published:** 2022-04-21

**Authors:** Yu-Chan Chang, Chien-Hsiu Li, Ming-Hsien Chan, Ming-Huang Chen, Chun-Nan Yeh, Michael Hsiao

**Affiliations:** 1 Department of Biomedical Imaging and Radiological Sciences, National Yang Ming Chiao Tung University, Taipei, Taiwan; 2 Genomics Research Center, Academia Sinica, Taipei, Taiwan; 3 Center of Immuno-Oncology, Department of Oncology, Taipei Veterans General Hospital, Taipei, Taiwan; 4 School of Medicine, National Yang Ming Chiao Tung University, Taipei, Taiwan; 5 Department of General Surgery, Liver Research Center, Chang Gung Memorial Hospital, Taoyuan, Taiwan; 6 Cancer Genome Research Center, Chang Gung Memorial Hospital, Linkou, Taoyuan, Taiwan; 7 Department of Biochemistry, College of Medicine, Kaohsiung Medical University, Kaohsiung, Taiwan

**Keywords:** Cancer therapy, Cell migration, Cell signalling, Gastrointestinal cancer

## Abstract

Cholangiocarcinoma (CCA) is a subtype of bile duct cancer usually diagnosed late with a low survival rate and no satisfactorily systemic treatment. Recently, regorafenib has been accepted as a second-line treatment for CCA patients. In this study, we investigated the potential signal transduction pathways mediated by regorafenib. We established a transcriptomic database for regorafenib-treated CCA cells using expression microarray chips. Our data indicate that regorafenib inhibits yes-associated protein 1 (YAP1) activity in various CCA cells. In addition, we demonstrated that YAP1 regulates epithelial-mesenchymal transition (EMT)-related genes, including E-cadherin and SNAI2. We further examined YAP1 activity, phosphorylation status, and expression levels of YAP1 downstream target genes in the regorafenib model. We found that regorafenib dramatically suppressed these events in CCA cells. Moreover, in vivo results revealed that regorafenib could significantly inhibit lung foci formation and tumorigenicity. Most importantly, regorafenib and amphiregulin (AREG) neutralize antibody exhibited synergistic effects against CCA cells. In a clinical setting, patients with high YAP1 and EMT expression had a worse survival rate than patients with low YAP1, and EMT expression did. In addition, we found that YAP1 upregulated the downstream target amphiregulin in CCA. Our findings suggest that AREG neutralizing antibody antibodies combined with regorafenib can reverse the CCA metastatic phenotype and EMT in vitro and in vivo. These findings provide novel therapeutic strategies to combat the metastasis of CCA.

## Introduction

Cholangiocarcinoma (CCA) is a malignant tumor that arises from bile duct cells in the biliary tree with a poor prognosis [1]. It is the second most common type of primary liver cancer and accounts for 10–20% of all primary liver cancers [2]. The most factor for favorable prognosis is tumor removal. Unfortunately, radical resection cannot be performed in most cases of CCA. Even with resection, the prognosis is dismal, with a five-year survival rate of only 10–44% [3].

Regorafenib is used to treat metastatic colorectal cancer patients who have previously received fluoropyrimidine-, oxaliplatin-, and irinotecan-based chemotherapy, anti-vascular endothelial growth factor (VEGF) therapy, and (if wild-type RAS) anti-epidermal growth factor receptor (EGFR) treatment [4–6]. Regorafenib can also be used to treat patients with unresectable or metastatic gastrointestinal stromal tumors who have previously received imatinib mesylate and sunitinib malate treatment, and hepatocellular carcinoma who have been previously treated with sorafenib [7]. Recently, a phase II trial showed regorafenib as a single agent with promising efficacy in patients with chemotherapy-refractory, advanced, and metastatic CCA [8].

Yes-associated protein 1 (YAP1) is involved in the Hippo pathway, which regulates various cancer phenotypes, especially angiogenesis, carcinogenesis, and metastasis [9]. YAP1 is a pivotal effector that acts as a transcriptional co-activator and is amplified to promote the epithelial to mesenchymal transition (EMT) and malignant transformation in cancers [10–12]. Amphiregulin (AREG) is produced as a transmembrane precursor that is proteolytically cleaved to produce the soluble factor. According to past studies, AREG was differentially overexpressed in human biliary cancers than the normal epithelium [13]. Furthermore, YAP1 and AREG have been reported to be independent prognostic factors for several cancer types [14–17].

In this study, we investigated the role of YAP1 in the occurrence and regulation of metastasis of CCA. We confirmed that regorafenib could inhibit the activity of YAP1 and affect the expression level of its downstream target AREG. Furthermore, we found that YAP1 enhanced CCA cells’ migration and invasion capabilities and promoted morphological changes during EMT. Therefore, we determined that reversing the phenotype induced by YAP1 is effective [18, 19]. In addition to reversing the YAP1-induced phenotype, we identified that regorafenib could also block tumorigenicity and lung nodule formation in vivo. Most importantly, these results were due to the synergistic effects of regorafenib combined with YAP1 downstream target blockers in vitro. This evidence supports a novel strategy for treating CCA patients.

## Materials and methods

### Cell lines and cell culture conditions

The cell lines HuCCT1, HuH28, SNU-1196, SNU-308, SNU-1079, and SNU-478 were maintained in a Roswell Park Memorial Institute medium containing 10% fetal bovine serum (FBS) and 1% penicillin-streptomycin-glutamine (PSG). KKU-100 and MMNK-1 cell lines were maintained in a Dulbecco’s modified eagle’s medium containing 10% FBS and 1% PSG. All cell lines were purchased from the JCRB cell bank. Human cholangiocyte primary cells were purchased from Celprogen (Cat. 36755–12). YAP1-expression (wt/Y357F) and knockdown were performed with Addgene and the RNAi core, respectively. Verteporfin (Cat.S2075, 10 μM) was purchased from Cayman (St. Louis, MO, USA). All inhibitors were soluble in dimethyl sulfoxide.

### Western blot

Western blot analysis was performed as previously described [20]. Antibodies directed against YAP1 (1:2000, GTX104091, Genetex), p-YAP1 (S127) (1:1000, #18625, IBL), p-YAP1 (Y357) (1:1000, #2101, Cell Signaling), E-cadherin (1:1000, #2109, Cell Signaling), SNAI2 (1:1000, #9101, Cell Signaling), vimentin (1:1000, #9102, Cell Signaling), CTGF (1:5000, GTX24232, Genetex) and α-tubulin (1:10,000, #T5168, Sigma-Aldrich) were used as primary antibodies. The blots were visualized using an ECL-Plus detection kit (PerkinElmer Life Sciences, Boston, MA, USA), Prestained protein marker (10-170kDa, Tools, Taiwna) and antibody hybridization was repeated with stripping buffer (TOOLSrip Buffer, Tools, Taiwan).

### Reverse transcription-PCR

The total RNA was extracted with a TRIzol reagent (#15596026, Thermo Fisher), and the cDNA synthesis was performed using the SuperScript™ IV first-strand synthesis system (#18091200, Thermo Fisher). Quantitative real-time PCR was performed using SYBR green PCR mix (Tools). The primer sequences used for real-time PCR were as follows: UHRF1-forward, 5′-AGG TCA ATG AGT ACG TCG ATG C-3′; UHRF1-reverse, 5′-TTC TCC GGG TAG TCG TCG T-3′; CTGF-forward, 5′-CAG CAT GGA CGT TCG TCT G-3′; CTGF-reverse, 5′-AAC CAC GGT TTG GTC CTT GG-3′; SNAI2-forward, 5′-GAG CAT ACA GCC CCA TCA CT-3′; SNAI2-reverse, 5′-GCA GTG AGG GCA AGA AAA AG-3′; BCL2L11-forward, 5′-TAA GTT CTG AGT GTG ACC GAG A-3′; BCL2L11-reverse, 5′-GCT CTG TCT GTA GGG AGG TAG G-3′; CCND1-forward, 5ʹ-TGG AGG TCT GCG AGG AAC A-3ʹ; CCND1-reverse, 5ʹ-TTC ATC TTA GAG GCC ACG AAC AT-3ʹ; CCNE1-forward, 5ʹ-AGC CAG CCT TGG GAC AAT AAT-3ʹ; CCNE1-reverse, 5ʹ-GAG CCT CTG GAT GGT GCA AT-3ʹ; CDKN1B-forward, 5ʹ-CTG CAAC CGA CGA TTC TTC TAC T-3ʹ; CDKN1B -reverse, 5ʹ-CTT CTG AGG CCA GGC TTC TT -3ʹ;CCN1-forward, 5ʹ-GGT CAA AGT TAC CGG GCA GT-3 ʹ; CCN1-reverse, 5ʹ- GGA GGC ATC GAA TCC CAG C-3 ʹ; PTGS2-forward, 5ʹ- GGC TTC CAT TGA CCA GAG CAG -3ʹ; PTGS2-reverse, 5ʹ- GCC GAG GCT TTT CTA CCA GA-3 ʹ; S26-forward, 5′-CCG TGC CTC CAA GAT GAC AAA G-3′; and S26-reverse, 5′-ACT CAG CTC CTT ACA TGG GCT T-3′.

### TEAD luciferase reporter assay

CCA cancer cells were co-transfected with 0.2 μg of a firefly luciferase reporter gene containing the sequence for transcriptional enhance associated domain (TEAD) and 0.02 μg of Renilla luciferase reporter [21]. The luciferase signals were measured by using a plate reader (Victor3, PerkinElmer) and ONE-Glo Luciferase assay (#E6120, Promega).

### Cell migration/ invasion assay

For migration assays, 8-μm pore size polycarbonate filters (GE Healthcare Life Sciences, Chalfont St. Giles, UK) were coated with 1 mg/mL human fibronectin (Sigma, St. Louis, MO, USA). A medium containing 10% FBS was added to the lower compartment and cells suspended in a serum-free medium were added to the upper compartment of the Boyden chamber. For invasion assays, fibronectin-coated polycarbonate filters were further coated with 10 mg/mL Matrigel (BD Biosciences, San Jose, CA, USA) on the opposite surface. Medium containing 10% FBS was added to the lower compartment and cells suspended in a serum-free medium were added to the Matrigel-coated upper compartment of the Boyden chamber. After the optimized timing (12–16 h), invading or migrating cells were stained with the Giemsa solution and counted under a light microscope (400×, eight random fields of each well). Three independent experiments were performed in quadruplicates.

### In vivo pulmonary nodules model

Age-matched, non-obese, diabetic-severe combined immune-deficient gamma (NOD.Cg-Prkdc^scid^ Il2rg^tm1Wjl^/SzJ JAX^®^, NOD-SCID γ) male mice (age: 6–8 weeks; body weight: 20–25 g) in a specific pathogen free (SPF) room were used for the experiments. All animal experiments were conducted in accordance with a protocol approved by the Academia Sinica Institutional Animal Care and Utilization Committee (IACUC) (IACUC No. 12–02–319). To evaluate the lung colony-forming ability, 1 × 10^6^ HuCCT1 cells were suspended in a 100 μL of phosphate-buffered saline (PBS), injected into the lateral tail vein of the mice, and randomly assigned to different groups (six mice/group), followed by oral feeding (Regorafenib, 30 mg/kg, five times per week) or intraperitoneally (i.p.) (Verteporfin, 100 mg/kg, twice a week) to each mouse on day 7. The lung nodule formation was quantified by hematoxylin and eosin staining of the specimens, followed by observation under a dissecting microscope at the endpoint of the experiment, where all mice were euthanized by CO_2_.

### In vivo tumorigenicity model

Age-matched, non-obese, and diabetic-severe combined immune-deficient gamma (NOD.Cg-Prkdc^scid^ Il2rg^tm1Wjl^/SzJ JAX^®^, NOD-SCID γ) male mice (age: 6–8-week old; body weight: 20–25 g) in a specific pathogen free (SPF) room were used in this study. All animal experiments were conducted in accordance with a protocol approved by the Academia Sinica Institutional Animal Care and Utilization Committee (IACUC) (IACUC No. 12-02-319). For the tumorigenicity assay, 5 × 10^6^ HuCCT1 cells were suspended in a 100 μL of PBS injected into the flank of the mice and randomly assigned to different groups (six mice/group), followed by oral feeding (Regorafenib, 30 mg/kg, 5 times per week) or intraperitoneally (i. p.) (Verteporfin, 100 mg/kg, twice a week) to each mouse on day 7. Every week, tumor growth was measured with a Vernier caliper, and the tumor size was calculated using the formula *V* = 1/2 (length × width^2^). At the end of the experiment, all mice were euthanized by CO_2_.

### Specimens

CCA tissue samples (*n* = 95) were collected from the Taipei Veterans General Hospital with the approval of the Institutional Review Board (2019-03-001BC). Histological diagnosis and staging of CCA were performed according to the recommendations of the World Health Organization (WHO) classification. The clinicopathological features, including tumor size, local invasion, lymph node involvement, and distal metastasis, were determined using the American Joint Committee on Cancer (AJCC) 7th edition staging classification of cholangiocarcinoma [22]. The follow-up period was 200 months. This was a retrospective clinical study and only archival paraffin-embedded samples of surgically removed tumors were used, and the study was not registered on the ClinicalTrials.gov website. Written informed consent for immunohistochemical tumor analysis was obtained from each patient. Finally, all samples were anonymized before histology and immunohistochemistry. Therefore, no further ethical approval was necessary to perform the study.

### Immunohistochemistry

YAP1 expression levels in the aforementioned 95 mass-forming cholangiocarcinoma (MF-CCA) patients were examined by immunohistochemical staining. Tissue sections (4 μm) were prepared from formalin-fixed, paraffin-embedded hepatectomy specimens and incubated with anti-YAP1 primary antibody (1:2000, GTX104091, Genetex) overnight at 4 °C. After three 5 min washes with TBST, the bound antibody signal was visualized using Dako Labeled Streptavidin-Biotin2 (LSAB2) System-HRP (Dako A/S, No. K0675; Dako, Glostrup, Denmark). Control slides were incubated with the secondary antibody only. To assess the immunohistochemical staining, the percentage of stained target cells was evaluated in 10 random microscopic fields of view per tissue section (×400 magnification), and their average scores were subsequently calculated. Staining intensities were scored as 1 (mild), 2 (moderate), or 3 (strong). H scores were calculated as the percentage of positive staining (0–100) × the corresponding staining intensity (0–3). Specimens with H-scores of <120 or ≥120 were classified as having low or high expression, respectively (range: 50–300; median 120).

### In silico study

Clinical information and genomics matrix file of The Cancer Genome Atlas (TCGA) database (https://xenabrowser.net/heatmap/) were downloaded from the United States Cancer Statistics (USCS) Xena browser website. All Gene Expression Omnibus Series (GSE) datasets downloaded from the Gene Express Omnibus (GEO) website were normalized and analyzed using the Genespring software (version 13.1.1., Agilent, Santa Clara, CA, USA) and the Kaplan–Meier plotter website (https://kmplot.com/analysis/). The downloaded datasets included clinical parameters and expression levels of target genes in CCA patients (TCGA_CHOL). This website applied microarray or next-generation sequencing for each probe after normalization. For high expression, the median was set to be higher than the high expression and vice versa. In addition, clinical cases that lacked relevant parameters were eliminated.

### Microarray establishment

Transcriptional profiling data were collected from the GEO database (GSE149131). Gene expression levels were normalized to log2 values using the GeneSpring software (Agilent Technologies, Palo Alto, CA, USA).

### Statistical methods

The Student’s *t*-test was used to analyze the statistical significance of results from at least three independent experiments, and the data are presented as mean ± SD. The clinicopathological characteristics were evaluated for differences using the chi-square test. Survival curves were analyzed using the log-rank test (generated by the Kaplan–Meier method). Univariate and multivariate models were constructed using Cox proportional hazards regression to evaluate the prognostic significance of the factors. All statistical tests were two-sided, and *p* < 0.05 was considered significant. Statistical analyses were performed using the SPSS (Statistical Package for the Social Sciences, version 19.0) software.

## Results

### Regorafenib inhibits the YAP1 signal transduction pathway and molecular events in CCA

**Fig. 1:**
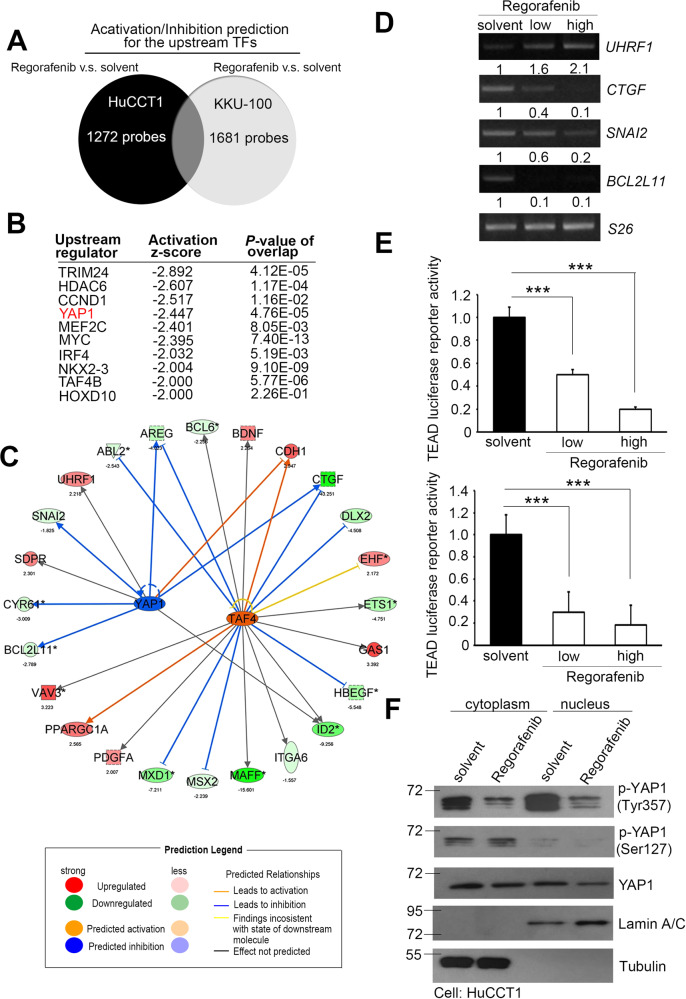
Regorafenib suppresses YAP1 signaling pathway in CCA cells. **A** Venn diagram of the potential transcription factors in HuCCT1 and KKU-100 cells of the regorafenib treatment group and solvent control group. **B** Ranking of the candidate upstream regulators of transcription inhibition from regorafenib treatment and solvent control groups predicted by Ingenuity pathway analysis with a 1.5-fold change cutoff. **C** Core analysis of the YAP1 and TAF4 signatures in regorafenib-treated and control HuCCT1 cells. **D** RT-PCR analysis of YAP1 downstream targets and S26 mRNA expression in regorafenib-treated HuCCT1 cells. **E** Luciferase reporter activity of TEAD in HuCCT1 and SNU-1079 cells treated with or without regorafenib. **F** Western blots showing YAP1 and p-YAP1 (Ser127 and Tyr357) protein levels after treatment of HuCCT1 cells with regorafenib (10 μM). Data are presented as the mean of three independent experiments ± SEM. The significance of the difference was determined using a nonparametric Mann–Whitney *U*-test. *p < 0.05, **p < 0.01, ***p < 0.001.

To investigate the importance of regorafenib in CCA, we established transcriptomic datasets by using microarray chips for regorafenib-treated CCA cells. HuCCT1 and KKU-100 cells were treated with low and high doses of regorafenib for 6 h (0.5 μM and 5 μM, respectively). With sample normalization and change cutoff of >1.5-fold, we selected features to predict the potential canonical pathways and upstream regulators (Fig. 1A and Supplementary Table S1). We observed that regorafenib downregulated the expression of YAP1 (Fig. 1B) and activated TAF4 (Fig. Supplementary Table S2). The core analysis of the results revealed several common downstream factors between YAP1 and TAF4 (Fig. 1C). Thus, through high-throughput screening analysis, we found that YAP1 is one of the transcription factors affected by regorafenib treatment. YAP1 has been claimed to play a dominant role in the tumorigenesis of cholangiocarcinoma, but the related molecular mechanism or clinical strategy remains unclear [23]. Therefore, we hypothesized that regorafenib could inhibit the activity of YAP1 in CCA. We further evaluated the expression levels of each downstream factor of YAP1, including *UHRF1*, *CTGF*, *SNAI2*, and *BCL2L11*, by RT-PCR analysis and found that some of the genes were regulated in a dose-dependent manner (Fig. 1D and Supplementary Fig. S1). Moreover, we confirmed these candidates in two CCA cells through q-PCR analysis and collected several previously known YAP1 downstream targets [24] (Supplementary Figs. S2, S3). In addition, a trend consistent with the ingenuity pathway analysis (IPA) software predictions was observed (Fig. 1C). This shows that after regorafenib treatment, it is possible to regulate these downstream factors through YAP1. These results confirmed that YAP1 plays an important role in CCA. The mammalian TEAD protein family contains YAP1/TAZ and DNA binding domains to construct a co-transcription complex [25]. Recently, the phosphorylation and translocation status of YAP1 has also been used as typical methods to evaluate its function [26, 27]. Therefore, we recruited TEAD reporter plasmids to confirm YAP1 activity and cytoplasmic/nucleus protein components to ensure that YAP1 translocation events occur in the regorafenib treatment model of CCA cells (Fig. 1E, F). Our results identified that regorafenib can interrupt YAP1 translocation status and suppress YAP1 activity thereby reducing downstream target expression.

**Fig. 2:**
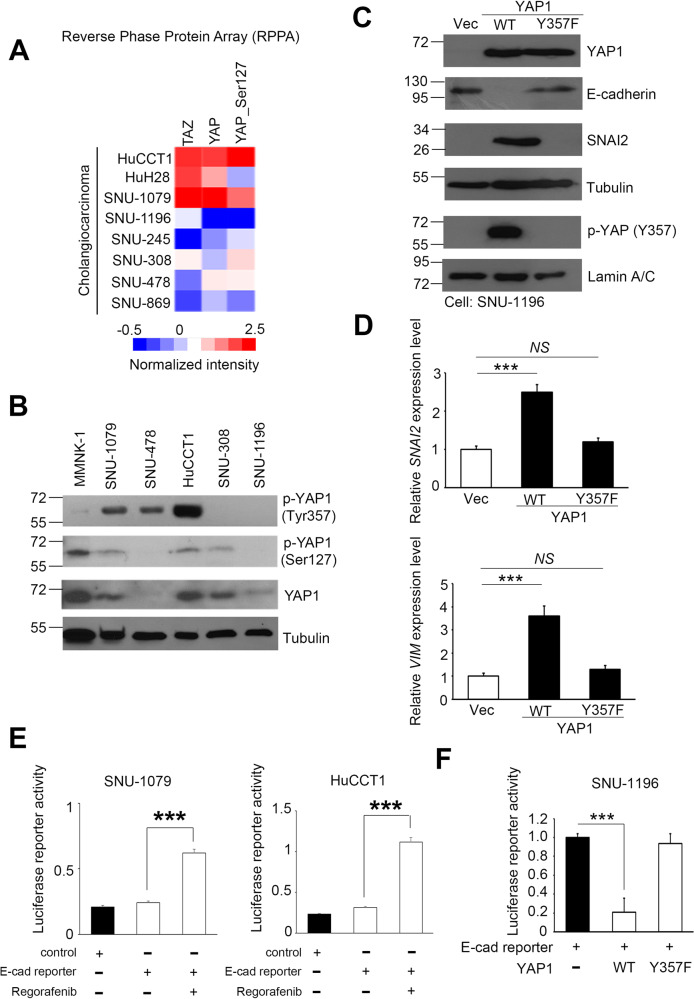
Regorafenib regulates YAP1 activity, phosphorylation, and EMT changes. **A** Heat map showing YAP1 and p-YAP1 (Ser127) levels in CCA in a reverse-phase protein array (RPPA) database. **B** Western blot analysis of YAP1 and p-YAP1 (Ser127 and Tyr357) levels in a CCA cell panel. **C** Western blot analysis of YAP1, p-YAP1 (Tyr357), and EMT-related protein levels in SNU-1196 cells with or without exogenous YAP1 (WT/Y357F). **D** Q-PCR analysis of EMT-related markers, *SNAI2*, and vimentin mRNA expression in SNU-1196 cells with or without exogenous YAP1 (WT/Y357F). **E** Luciferase reporter activity of E-cadherin in HuCCT1 and SNU-1079 cells treated with or without regorafenib. **F** Luciferase reporter activity of E-cadherin in SNU-1196 cells treated with or without exogenous YAP1 (WT/Y357F). Data are presented as the mean of three independent experiments ± SEM. The significance of the difference was determined using a nonparametric Mann–Whitney *U*-test. ***p < 0.001.

In addition to drug treatment of cells, we also analyzed the protein levels of TAZ, YAP1, and phosphorylated YAP1 (p-YAP1; Ser127) in CCA using the cancer cell line encyclopedia reverse-phase protein array (CCLE_RPPA) database [28]. The heat map showed that HuCCT1 and SNU-1079 had higher levels of p-YAP1 than other CCA cells (Fig. 2A). Previous studies have shown that YAP1 accumulates on the transcription factor FOS and activates the transcriptional program involved in EMT regulation [29, 30]. Even the phosphorylated YAP1 (Ser127) can bind to 14-3-3 protein and remains in the cytoplasm for degradation [31]. However, another phosphorylated form of YAP1 (Y357) confirmed that YAP is located in the nucleus and constitutive signaling pathways [32]. Due to the lack of the specific phosphorylation form of YAP1 (Y357) in the RPPA profile, we further measured p-YAP1 (Y357) levels in various CCA cells by western blot analysis. We also validated the expression and translocation status of p-YAP1 (S127), p-YAP1 (Y357), and YAP1 in initial experiments. The results shown are consistent with the trends obtained from the RPPA profile and our previous research (Fig. 2B and Supplementary Fig. S4). In addition, we calculated EMT scores based on an algorithm from a previous study [33]. We observed that EMT scores were higher (M > E) in HuCCT1, HuH28, and SNU-1079 cells, while SNU-478, SNU-308, SNU-1196, and SNU-245 cells had lower EMT scores (E > M) (Supplementary Fig. S5). These phenotypes were closely related to the phosphorylation status of YAP1 (Fig. 2A, B). Based on these evidences, we selected the lower expressing YAP1 phosphorylated CCA cells to explore whether the degree of phosphorylation at Tyr357 sit affects EMT events. We established an exogenous YAP1 model containing wild-type or Y357F mutant sequences in SNU-1196 cells and compared the mRNA levels of EMT-related markers, the protein, and phosphorylation status of YAP1. Our data indicate that Y357 has an important function in the translocation of YAP1 to the nucleus and regulating downstream EMT-related markers. Once the tyrosine structure is altered, YAP1 activity will be deprived (Fig. 2C, D).

Moreover, we measured the reporter activity of E-cadherin in the regorafenib treatment model. The luciferase reporter assay showed that the E-cadherin activity was restored in the treatment group than the solvent control group. At the same time, the reverse model demonstrated that YAP1 did not inhibit downstream E-cadherin expression when the Y357 phosphating site was mutated (Fig. 2E, F). Similarly, in this model, mesenchymal-related marker proteins are also suppressed (Supplementary Fig. S6). Therefore, we propose that YAP1 is an important transcription factor for CCA tumorigenesis and is inhibited by regorafenib.

### Regorafenib can reduce the cell proliferation and tumorigenicity

**Fig. 3:**
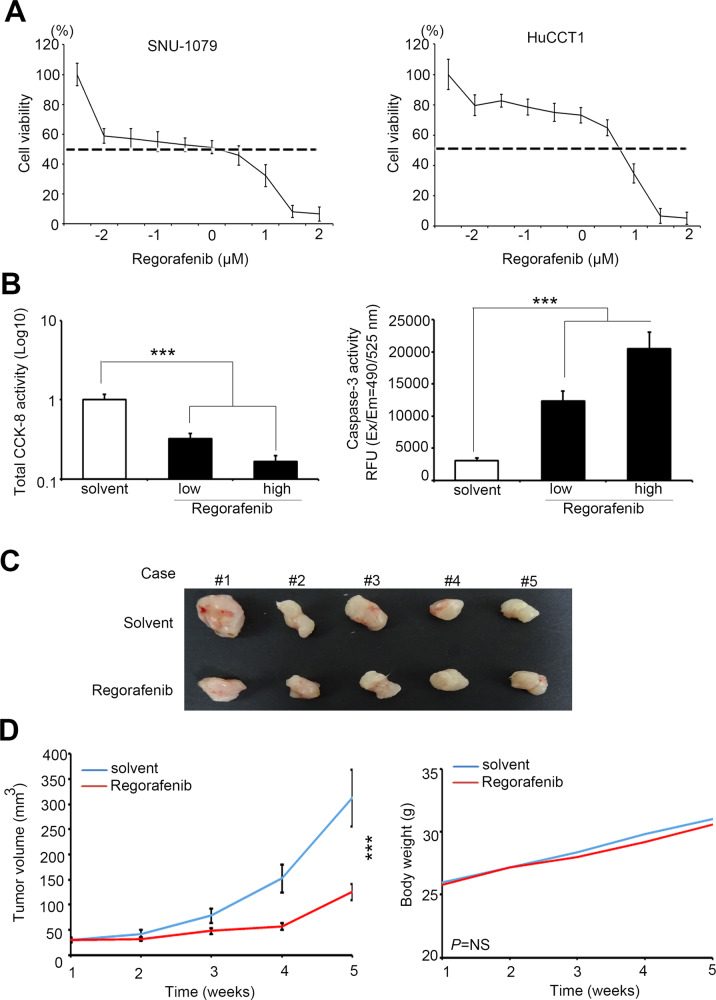
Regorafenib also interferes cell viability and tumorigenesis of CCA cells. **A** Viability of SNU-1079 and HuCCT1 cells after regorafenib treatment. **B** Quantitative CCK-8 activity and Caspase 3 activity with or without regorafenib treatment in CCA cells. **C** HuCCT1 cells were subcutaneous injected into NOD-SCID mice that were treated over an interval of 2 day with saline only (PBS) and regorafenib. **D** Quantified plot of the growth curve and body weight in each group. Data are presented as the mean of three independent experiments ± SEM. The significance of the difference was determined using a nonparametric Mann–Whitney *U*-test. *p < 0.05, **p < 0.01, ***p < 0.001.

To validate our hypothesis that YAP1 modulates the various phenotypes of CCA, we measured the IC50 of regorafenib in several CCA cells (Fig. 3A). Our results also showed that regorafenib is more effective on CCA cell viability than other available YAP1 inhibitors, Verteporfin (Supplementary Fig. S7A). In the cell model, the regorafenib decrease the CCA cell proliferation rate and induced apoptosis (Fig. 3B and Supplementary Fig. S7B, C). Similarly, we have noticed that the significant effect of regorafenib was able to suppress tumorigenicity in the mouse model (Fig. 3C and Supplementary Fig. S7D). After the experimental endpoint, we collected solid tumors from the subcutaneous group mice (*n* = 5 in each group). Furthermore, the tumor volume decreased in the regorafenib group compared to solvent control. Most importantly, the regorafenib-related procedure did not induce strong cytotoxicity (Fig. 3D).

### Regorafenib inhibits YAP activity, EMT status, and metastatic properties in CCA models

**Fig. 4:**
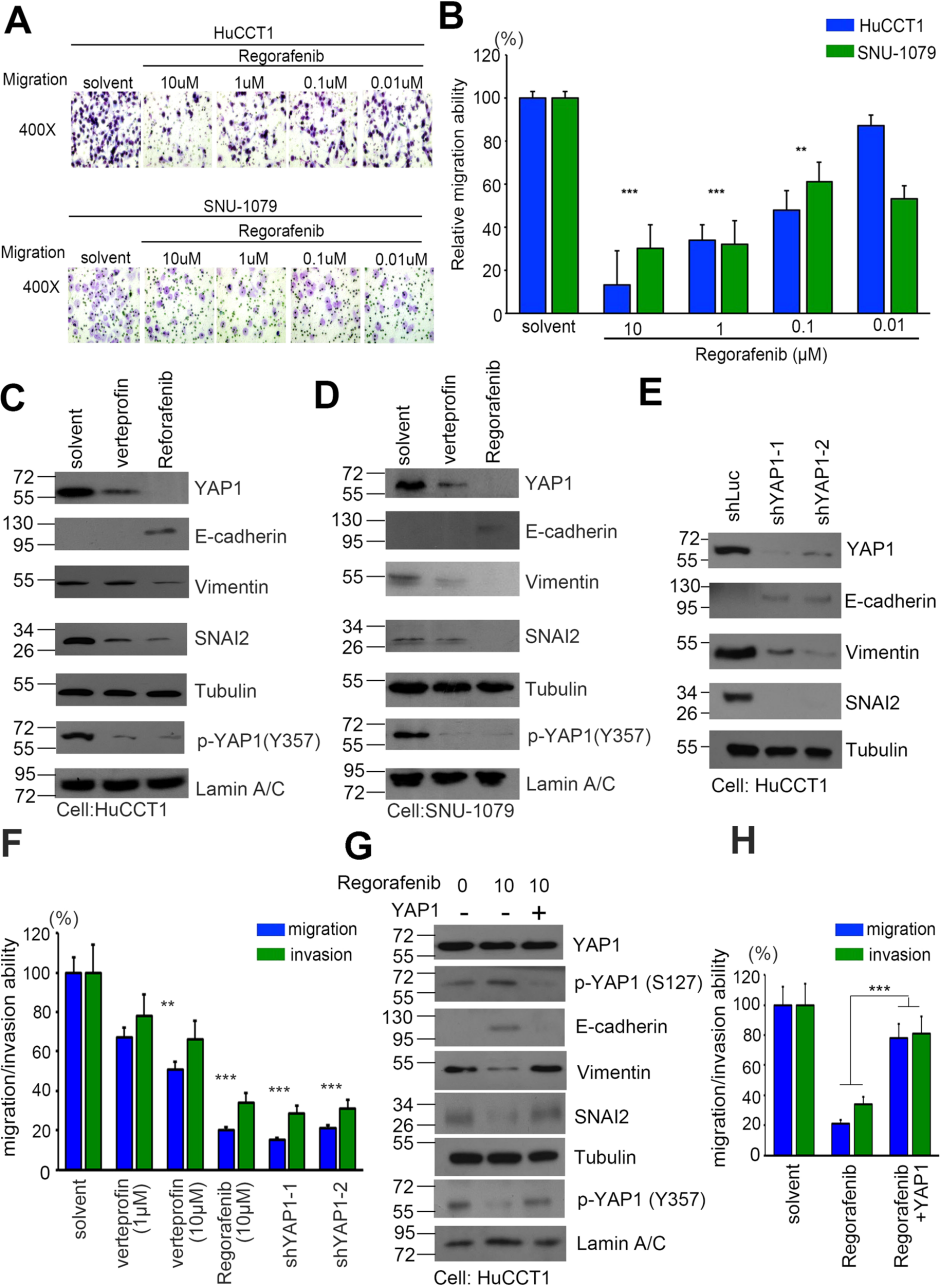
YAP1 triggers the migration/invasion ability of EMT and CCA cells. **A** Migratory abilities of HuCCT1 and SNU-1079 cells treated with regorafenib (0.01, 0.1, 1, and 10 μM), evaluated by Giemsa staining. **B** Quantitation of migration ability of HuCCT1 and SNU-1079 cells treated with regorafenib (0.01, 0.1, 1, and 10 μM). **C** Western blots showing E-cadherin, vimentin, and SNAI2 protein levels after treatment of HuCCT1 cells with YAP1 inhibitors verteporfin (10 μM) and regorafenib (10 μM), respectively. **D** Western blots showing E-cadherin, vimentin, and SNAI2 protein levels after treatment of SNU-1079 cells with YAP1 inhibitors verteporfin (10 μM) and regorafenib (10 μM), respectively. **E** Western blots show E-cadherin, vimentin, and SNAI2 protein levels after treating HuCCT1 cells with YAP1 shRNAs, respectively. **F** Quantitation of migration/invasion ability of HuCCT1 cells treated with or without verteporfin (1 μM and 10 μM), regorafenib (10 μM) and YAP1 shRNAs, respectively. **G** Western blots showing phospho-YAP1, YAP1, E-cadherin, vimentin, and SNAI2 protein levels after treatment of HuCCT1 cells with regorafenib (10 μM) or combined with YAP1 overexpression plasmids, respectively. **H** Quantitation of migration/invasion ability of HuCCT1 cells treated with regorafenib (10 μM) with or without YAP1 overexpression plasmids, respectively. Data are presented as the mean of three independent experiments ± SEM. The significance of the difference was determined using a nonparametric Mann–Whitney *U*-test.

Moreover, we evaluated various phenotypes of HuCCT1 and SNU-1079 cells after exposure to regorafenib in vitro and in vivo. According to previous literature, YAP1 has a variety of roles in cancer cells, including those associated with CCA [34]. As well as the proliferation and tumorigenic activity of cancer cells. It also modulates EMT changes and migration/invasion capabilities [29, 35]. We then added regorafenib to cells in the Boyden chambers to investigate their effects on CCA cells’ migration and invasion capabilities (Fig. 4A). Quantitative cell count, migration of HuCCT1 and SNU-1079 cells were inhibited in the regorafenib treatment group (Fig. 4B). We further determined the effects of these inhibitors on the RNA and protein expression levels of several EMT-related markers, including E-cadherin, vimentin, and SNAI2. We found that E-cadherin expression levels were increased in inhibitor-treated CCA cells. In contrast, SNAI2 and vimentin levels were decreased in this model (Fig. 4C, D). To determine if YAP1 activity is affected, we recruited verteporfin, an available YAP1 inhibitor, to compare effects on CCA cell migration/invasion. Furthermore, we generated a YAP1 knockdown stable cell model to ensure that YAP regulates EMT changes and functions (Fig. 4E, F). In addition, we used a complementary model of regorafenib treatment and exogenously expressed YAP1. Our results indicated that regorafenib inhibited YAP1 activity as well as modulated the expression of markers associated with EMT, inhibiting CCA cells migration and invasion (Fig. 4G, H).

**Fig. 5:**
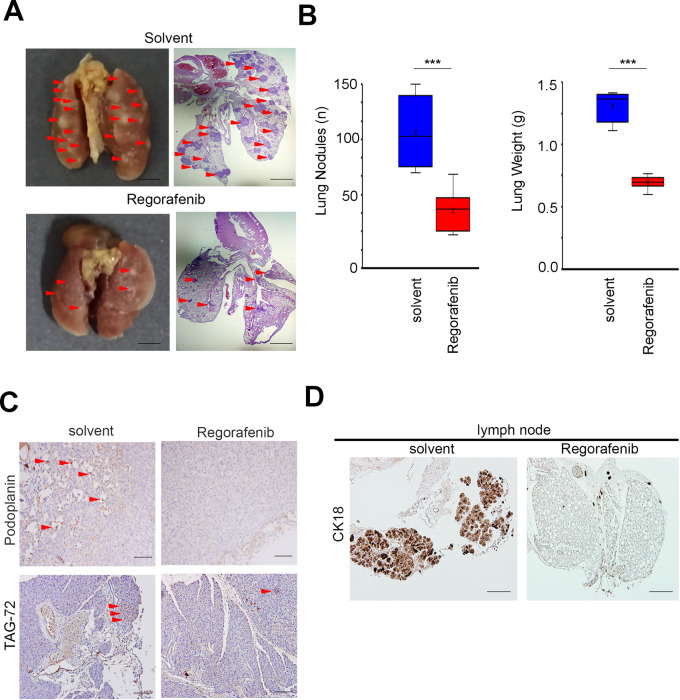
Regorafenib inhibits lung foci formation and metastatic ability of CCA in vivo. **A** Metastatic lung foci (indicated by arrows in the left panel) in mice (*n* = 6) implanted with HuCCT1 cells through tail vein injection and foci morphology (middle panel, ×12.5 magnification and right panel, ×100 magnification). **B** Quantified plot of the number of metastatic lung foci in each group. **C** Representative IHC staining intensity of podoplanin and TAG-72 protein in lymph nodes of CCA tissues. **D** Representative IHC staining intensity of CK18 protein in CCA tissues. Scale bar: 50 µm. Scale bar: 50 µm. Data are presented as the mean of three independent experiments ± SEM. The significance of the difference was determined using a nonparametric Mann–Whitney *U*-test.

To observe whether similar trends in animal studies. We further established CCA animal models through intravenous injections. After randomization, treatments were administered daily by oral feeding (30 mg/kg in 100 μL per day). Multiple organs were collected from the animals at the experimental endpoint, and solid tumors were used for further experiments. In the intravenous model, regorafenib significantly reversed these phenotypes than solvent control (Fig. 5A). Our results also showed that the number of lung nodules and total lung weight after treatment were lower than solvent control (Fig. 5B). In addition, we found that expression of the lymph node invasion markers podoplanin, tumor-associated glycoprotein 72 (TAG-72), and cytokeratin 18 (CK18) were lower in the combination group as compared to the solvent control group (Fig. 5C, D) [36, 37]. Thus, Regorafenib could induce mesenchymal-epithelial transition and block metastatic ability in CCA in vitro and in vivo.

### Regorafenib regulates AREG expression via the YAP pathway

**Fig. 6:**
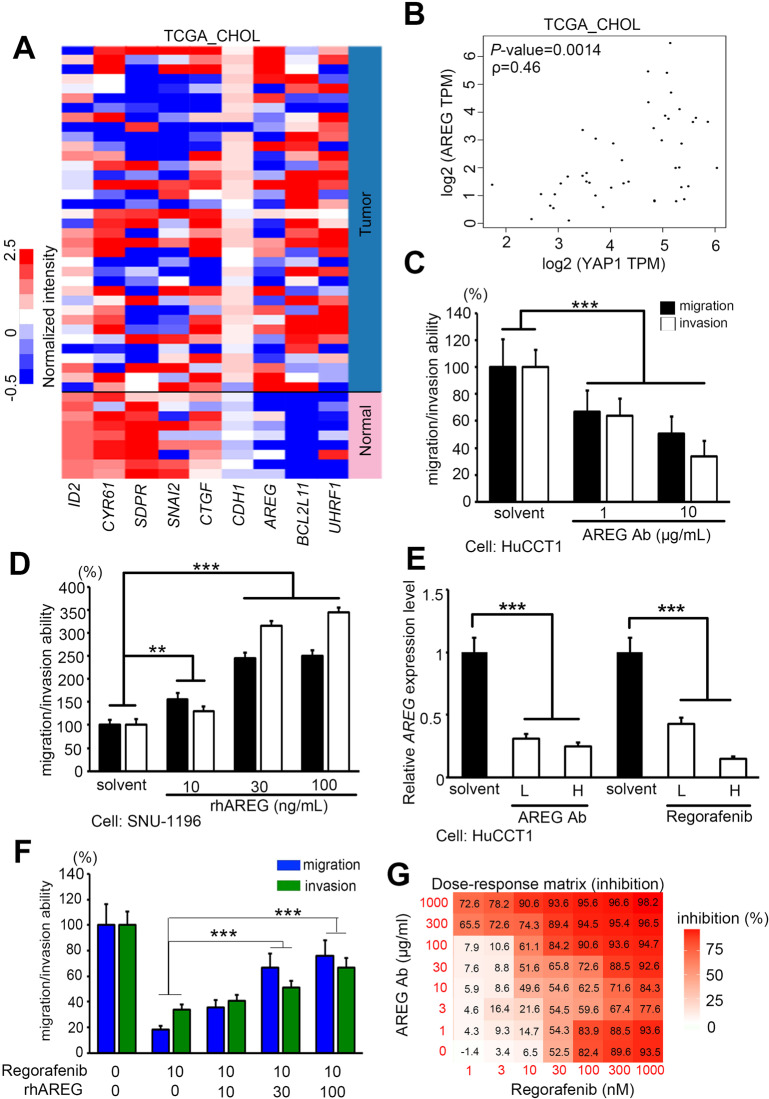
YAP1 modulates migration/invasion capability through downstream target AREG. **A** Heat map of the expression of YAP1 downstream genes from the TCGA RNA sequencing database in noncancerous and tumor tissues derived from CCA patients. **B** Correlation between YAP1 and AREG mRNA levels in TCGA_CHOL clinical patients (*ρ* = 0.46, *p* = 0.0014). **C** Treatment with anti-AREG antibody suppressed the invasive ability of HuCCT1 cells. Cells treated with anti-AREG antibodies (1 and 10 µg/mL) were seeded into the upper well of the Boyden chamber with Matrigel and subjected to invasion assays for 18 h. The columns represent mean values from three independent experiments. Bars: means ± SD. **D** AREG recombinant protein treatment increased the invasive ability of SNU-1196 cells. Cells with AREG recombinant protein (10, 30, and 100 ng/mL) were seeded into the upper well of the Boyden chamber with Matrigel and subjected to invasion assays for 18 h. The columns represent mean values from three independent experiments. Bars: means ± SD. **E** qRT-PCR analysis of AREG mRNA expression in HuCCT1 cells treated with AREG neutralize antibody or regorafenib. **F** Quantitation of migration/invasion ability of HuCCT1 treated with regorafenib (10 μM) with or without combined AREG recombinant protein (10, 30, and 100 ng/mL). **G** Calculate the synergy of the combined use of regorafenib and AREG neutralize antibody through the SynergyFinder service. Data are presented as the mean of three independent experiments ± SEM. The significance of the difference was determined using a nonparametric Mann–Whitney *U*-test. *p < 0.05, **p < 0.01, ***p < 0.001.

To determine the role of YAP1 in the migration/invasion of CCA, we examined the expression levels of YAP1 downstream genes from our microarray datasets. After qPCR analysis and applying a >1.5-fold change cutoff, we found that several genes may play important roles in CCA by targeting YAP1 directly in its promoter region. The expression of these genes was significantly altered in primary tumors as compared to normal adjacent tissues (Fig. 6A). We further determined the correlation between YAP1 and various downstream factors in cholangiocarcinoma patients. Our data showed that AREG is the most pivotal factor (Fig. 6B and Supplementary Fig. S8). Recently, it was demonstrated that AREG expression levels are related to proliferation, and the EGFR signaling pathway is activated in a YAP1-dependent manner [38, 39]. However, the detailed function of AREG in CCA has rarely been studied. Therefore, we screened candidate genes in a CCA cell panel (Supplementary Fig. S9A). Through human cholangiocytes, benign cells MMNK-1 and malignant cells HuCCT1, we also observed an increase in AREG in CCA cancer progression (Supplementary Fig. S9B). To confirm that YAP1 can indeed regulate downstream AREG expression in our model, AREG expression was re-examined in verteporfin-treated and shYAP-stablized cells. These results indicated that YAP1 regulates AREG in CCA cells (Supplementary Fig. S10A). In contrast, YAP1 overexpression restored AREG expression in the regorafenib treated model (Supplementary Fig. S10B). These observations demonstrate that AREG is one of the downstream factors of YAP1 in CCA cells. We also applied AREG neutralized antibodies to compete with the ligand in the malignant cell line HuCCT1. The results showed that AREG antibodies could inhibit the migratory/invasive abilities of HuCCT1 in a dose-dependent manner (Fig. 6C). On the other hand, the benign cell line SNU-308 with recombinant AREG protein exhibited increased migratory and invasive ability. Moreover, AREG enhanced the migratory/invasive abilities of CCA cells in a dose-dependent manner (Fig. 6D). In addition, we examined the mRNA and protein expression levels of AREG, including regorafenib and AREG neutralized antibodies (Fig. 6E and Supplementary Fig. S11). Our results confirmed that AREG is located downstream of YAP1, and its expression is suppressed by regorafenib and AREG antibodies (Fig. 6E). Through the complementary model, we found that the AREG recombinant protein can partially reverse the migration/invasion ability inhibited by regorafenib (Fig. 6F). Therefore, we evaluated novel synergistic drug combinations in our cell models. We tested the cell viability of 7 concentrations of regorafenib and 8 conditions of AREG antibodies in the same CCA cell line. Through the SynergyFinder server (https://synergyfinder.fimm.fi) [40], we clearly obtained appropriate doses of combined regorafenib and AREG antibodies (Fig. 6G). These combinations show that when regorafenib >10 nM and AREG > 10 μg, the combined use of the two can have a significant inhibitory effect instead of the high dose of a single drug. These combination options can be used in other therapy strategies for CCA.

### YAP1 was correlated with clinical parameters in patients with cholangiocarcinoma

**Fig. 7:**
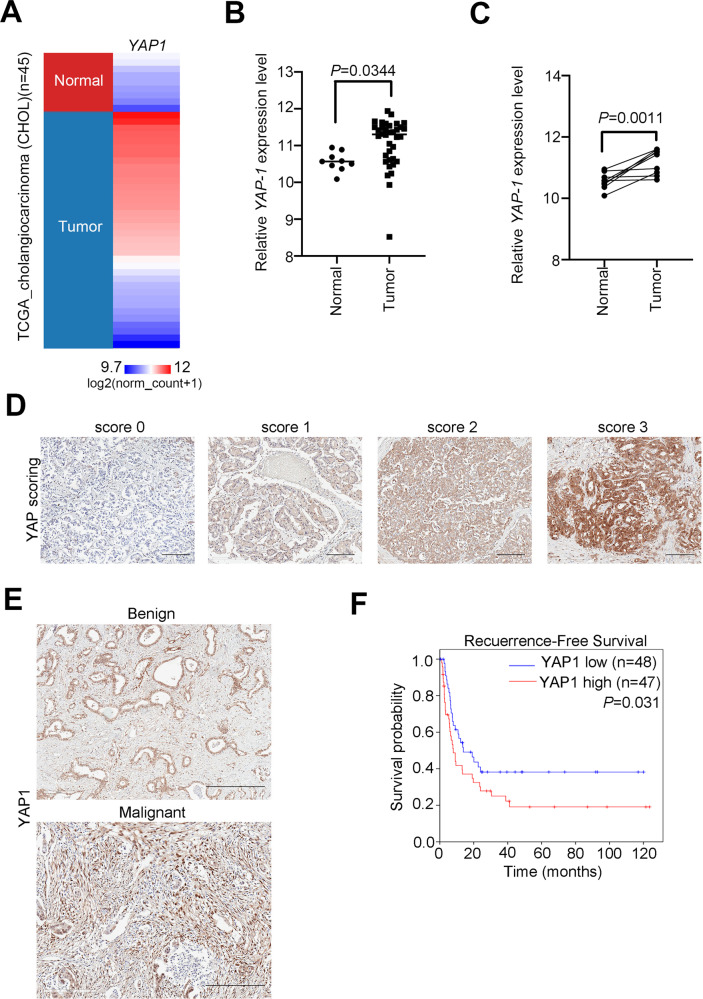
YAP1 expression level correlates with poor survival and clinicopathological features in CCA patients. **A** Heat map of YAP1 gene expression from the TCGA RNA sequencing database in noncancerous and tumor tissues derived from CCA patients. **B** YAP1 gene expression from the TCGA RNA sequencing database in unpaired noncancerous and tumor tissues derived from CCA patients. **C** YAP1 gene expression from the TCGA RNA sequencing database in paired noncancerous and tumor tissues derived from CCA patients. **D** Representative IHC staining intensity of YAP1 protein in CCA tissues. Scale bar: 50 µm. **E** Representative IHC staining for YAP1 protein expression in benign and malignant tissues derived from patients with CCA. Scale bar: 50 µm. **F** Kaplan–Meier plots of recurrence-free survival of 95 CCA patients underwent hepatectomy according to YAP1 expression. The significance of the difference was determined using the nonparametric Mann–Whitney *U*-test.

**Fig. 8:**
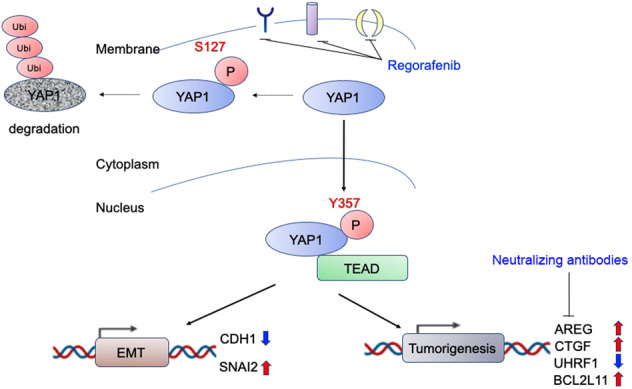
Schematic model of this study. Evidence was pooled to illustrate the combined approach as a potential treatment option targeting YAP1-AREG axis for CCA patients.

To evaluate the prognostic value of YAP1 in CCA patients, we detected the expression level of YAP1 in the TCGA cohort by an in silico analysis (Fig. 7A). We found that the expression of YAP1 in the primary tumors was higher than that in the adjacent normal tissues (Fig. 7B, *p* = 0.0344). Moreover, YAP1 was performed, resulting in the same patient's normal tissues and tumor pairing (Fig. 7C, *p* = 0.0011). We further measured the expression level of YAP1 in the available tissue arrays by immunohistochemical staining (Fig. 7D). After quantitation and scoring, we observed that YAP1 expression was higher in malignant than in benign tumor samples. In addition, we observed high levels of nuclear staining for YAP1 in malignant tissues (Fig. 7E). Through univariate and multivariate analyses of the clinical cases, we confirmed that YAP1 was an independent prognostic factor, and its expression level was correlated with recurrence-free survival in the clinical cohort (Supplementary Tables 3, 4). In our tissue array cohort, we identified that YAP1 has a prognostic value for the recurrence-free survival of CCA, and we determined the relationship between AREG, p-YAP1 (Tyr 357), and p-YAP1 (Ser 127) (Fig. 7F and Supplementary Fig. S12). We found that AREG and p-YAP1 (Tyr357, nuclear form) are positively correlated in CCA and negatively correlated with p-YAP1 (Ser 127, cytoplasmic form). Combining all evidence, we conclude that regorafenib dramatically inhibits metastasis and EMT progression and acts by regulating the YAP1-AREG axis in CCA (Fig. 8).

## Discussion

Regorafenib is a multitargeted tyrosine kinase inhibitor of VEGFR-1/2/3, KIT, RET, RAF1, BRAF, PGDFR, and FGFR [41]. Regorafenib is considered a potential therapeutic agent for CCA as several molecular alterations [42], particularly disruption of the MAPK pathway and activation of Ras and BRAF by mutations, have been described in CCA [43–45]. In addition, our previous study identified MALT1 as the downstream mediator of the Raf/Erk/Elk-1 pathway and its potential to serve as a new therapeutic target for the successful treatment of CCA with regorafenib [20]. The clinical therapeutic efficacy of regorafenib was also approved in a recent phase II clinical trial with an 11% response rate and 40% survival rate at 12 months in patients with chemotherapy-refractory CCA [8].

MMNK-1 cells were obtained by immortalizing cholangiocytes. Therefore, MMNK-1 cells may contain various unknown genetic alterations and epigenetic modifications. In addition, MMNK-1 cells have a high degree of differentiation and proliferation [46]. This may be caused by YAP1 overexpression. For this purpose, we purchased primary human cholangiocytes from the company to compare the available CCA cells. We observed that the expression level of YAP1 in cholangiocytes was lower than that in CCA cells (Supplementary Fig. S13). We also examined ALOX5 as a positive control and recently reported that it functions as an oncogene in the CCA model [47]. Thus, our evidence shows that MMNK-1 is not an appropriate group under certain conditions, but it needs to pass through human cholangiocytes to reflect the reality alterations between normal and tumor.

In the present study, we demonstrated that regorafenib could inhibit EMT through the YAP1 pathway. However, one of the limitations of our findings is that the mechanism by which regorafenib inhibits the YAP1 pathway remains unclear. Rizvi et al. demonstrated a fibroblast growth factor (FGF)-YAP-FGFR autocrine loop that drove oncogenic signaling in multiple CCA models. Downregulation of FGF signaling in these models by a small molecule inhibitor, BGJ398, disrupted this autocrine loop and inhibited YAP activity [48]. Smoot RL et al. demonstrated that crenolanib, a small molecule inhibitor, could downregulate PDGF signaling, which is associated with the downregulation of SRC family kinase activity, YAP tyrosine phosphorylation, and CCA cell viability [49]. These observations partially explain the mechanism of YAP pathway inhibition by regorafenib. Thus, Regorafenib is a multitargeted tyrosine kinase inhibitor that affects both FGFR and PDGFR. However, the underlying mechanism remains unclear and needs to be further explored.

YAP1 is an important transcription factor with multiple downstream targets, and E-cadherin (*CDH1*) and SLUG (*SNAI2*), which regulate cell morphology during EMT, have been widely studied. In this study, we determined the expression levels of other downstream genes, including *UHRF1* and *BCL2L11*, by RT-PCR, and found that *AREG* was one of the most significant downstream targets of YAP1. Our results also demonstrated that the expression levels of *UHRF1* and *BCL2L11* were higher in malignant cells than in adjacent normal tissues. Furthermore, we analyzed the expression levels of *UHRF1* and *BCL2L11* in the TCGA_CHOL cohort and confirmed that *UHRF1* and *BCL2L11* were highly expressed in tumor tissues than in the adjacent normal tissues. These results suggest that YAP1 regulates new phenotypes and functions during tumorigenesis.

YAP expression regulates the crosstalk between immune cells and tumor cells in the tumor microenvironment through its influence on T cells, myeloid-derived suppressor cells, and macrophages [50]. Therefore, it is reasonable that regorafenib might regulate the tumor microenvironment by inhibiting the YAP pathway. A phase 1b study showed that regorafenib enhanced the efficacy of immune checkpoint inhibitors by inhibiting regulatory T cells and tumor-associated macrophages in microsatellite stable colorectal and gastric cancer [51]. However, further investigations are necessary to prove this hypothesis.

## Conclusion

In summary, our study demonstrated that regorafenib inhibits tumorigenesis of CCA both in vitro and in vivo. Through the established transcriptome dataset, we confirmed that YAP1 plays an important role in the efficacy of regorafenib. We also found that YAP1 promotes EMT and increases AREG expression levels. Furthermore, we supplied a combination therapy of regorafenib and AREG neutralize antibody, and found that they had synergistic effects against CCA (Fig. 8).

## Supplementary information

Supplementary profile

Author contributions

Reproducibility checklis

Supplemental Table S1

original WB blots

## Data Availability

All data needed to evaluate the conclusions in the paper are present in the paper. Additional data related to this paper may be requested from the corresponding author.
