# Supplementary material for: Regorafenib inhibits epithelial-mesenchymal transition and suppresses cholangiocarcinoma metastasis via YAP1-AREG axis
Source: Cell Death Dis. 2022 Apr 21;13(4):391. doi: 10.1038/s41419-022-04816-7 (PMC9023529; doi:10.1038/s41419-022-04816-7)

**Supplementary Information**

**Regorafenib Inhibits Epithelial-Mesenchymal Transition and Suppresses Cholangiocarcinoma Metastasis**

Yu-Chan Chang^1^, Chien-Hsiu Li^2^, Ming-Hsien Chan^2^, Ming-Huang Chen^3,4#^ Chun-Nan Yeh^5,6#^, and Michael Hsiao^2,7#^

^1^ Department of Biomedical Imaging and Radiological Sciences, National Yang Ming Chiao Tung University, Taipei, Taiwan

^2^ Genomics Research Center, Academia Sinica, Taipei, Taiwan

^3^ School of Medicine, National Yang Ming Chiao Tung University, Taipei, Taiwan

^4^ Center of Immuno-Oncology, Department of Oncology, Taipei Veterans General Hospital, Taipei, Taiwan

^5^ Department of General Surgery, Liver Research Center, Chang Gung Memorial Hospital, Taoyuan, Taiwan

^6^ Cancer Genome Research Center, Chang Gung Memorial Hospital, Linkou, Taoyuan, Taiwan.

^7^ Department of Biochemistry, College of Medicine, Kaohsiung Medical University, Kaohsiung, Taiwan.

#To whom correspondence should be addressed:

Dr. Michael Hsiao, Genomics Research Center, Academic Sinica, 128 Academia Road, Section 2, Taipei 115, Taiwan. Phone: 886-2-2787-1243; Fax: 886-2-2789-9931; E-mail: [mhsiao@gate.sinica.edu.tw](mailto:mhsiao@gate.sinica.edu.tw)

Or

Dr. Chun-Nan Yeh, Department of General Surgery, Liver Research Center, Chang Gung Memorial Hospital, Linkou, Taoyuan 333, Taiwan. Phone: 886-2-3281200; E-mail: yehchunnan@gmail.com.

Or

Dr. Ming-Huang Chen, Center of Immuno-Oncology, Department of Oncology, Taipei Veterans General Hospital, 201 Shipai Road, Section 2, Taipei, 112, Taiwan. Phone: 886-2-2875-7270; E-mail: mhchen9@vghtpe.gov.tw

**Running Title:** Regorafenib inhibits cancer metastasis in cholangiocarcinoma

**Conflict of interest:** The authors disclosed no conflicts

**Supplemental Table 1**. List of microarray probes with a cutoff change of > 1.5-fold.

| **Upstream regulator** | **Activation Z-score** | ***p*-value of overlap** |
| --- | --- | --- |
| IRF1 | 3.5 | 1.33E-09 |
| IRF7 | 3.461 | 4.05E-10 |
| STAT1 | 3.073 | 3.97E-13 |
| IRF5 | 2.578 | 1.70E-06 |
| NAFTC2 | 2.578 | 1.29E-04 |
| IRF3 | 2.577 | 3.51E-12 |
| TAF4 | 2.576 | 1.48E-06 |
| MSC | 2.407 | 4.45E-04 |
| ATF4 | 2.333 | 8.18E-12 |
| PHB2 | 2.331 | 1.60E-04 |
| SMAD5 | 2.236 | 5.01E-04 |
| STAT2 | 2.067 | 1.23E-05 |
| NFKNIA | 2.015 | 3.97E-20 |

**Supplemental Table 2**. Ranking of the candidate upstream regulators of transcription activation from regorafenib treatment and solvent control groups predicted by Ingenuity pathway analysis with a 1.5-fold change cutoff.

Supplemental Table 3. Clinicopathological features and YAP1 expression in cholangiocarcinoma patients.

|  | YAP-1 low expression  (n = 50) | YAP-1 high expression  (n = 55) | *p* |
| --- | --- | --- | --- |
| Age |  |  | 0.642 |
| <65y | 26(52.0%) | 28(56.0%) |  |
| >65y | 24(48.0%) | 27(44.0%) |  |
| Gender |  |  | 0.642 |
| Male | 22(44.0%) | 30(54.5%) |  |
| Female | 28(56.0%) | 25(45.5%) |  |
| Tumor status |  |  | 0.211 |
| T1+T2 | 32(64.0%) | 20(38.5%) |  |
| T3+T4 | 18(36.0%) | 32(61.5%) |  |
| Lymph node status |  |  | 0.071 |
| N0 | 30(76.1%) | 20(45.5%) |  |
| N1-3 | 20(23.9%) | 35(54.5%) |  |
| TNM stage |  |  | 0.967 |
| I+II | 24(48.0%) | 16(29.1%) |  |
| III+IV | 26(52.0%) | 39(70.9%) |  |
| Recurrence |  |  | 0.967 |
| No | 15(30.0%) | 24(43.6%) |  |
| Yes | 35(70.0%) | 31(56.4%) |  |
|  |  |  |  |

**p* < 0.05 was considered statistically significant (Student’s *t*-test for continuous variables and Pearson’s chi-square test for discrete variables). SD represents the standard deviation. ^#^tumor stage, tumor, lymph node, and distal metastasis status were classified according to the International System for Staging Lung Cancer.

**Supplemental Table 4**. Univariate analysis of YAP1 expression in cholangiocarcinoma.

| Cox univariate analysis (OS) | | |  |  |  |
| --- | --- | --- | --- | --- | --- |
| Variables | | Comparison | HR (95% CI) | | *P*-value |
| T |  | T3-T4 vs. T1-T2 | 1.917 (1.581-3.410) | | 0.0029 |
| N |  | N1-N3 vs. N0 | 2.503 (1.663-3.791) | | <0.0001^*^ |
| Stage |  | III-IV vs. I-II | 3.227 (1.812-4.905) | | <0.0001^*^ |
| YAP-1 |  | High vs. Low | 2.511 (1.520-3.880) | | <0.0001^*^ |
| Cox multivariate analysis (OS) | | |  |  |  |
| Variables | | Comparison | HR (95% CI) | | *P*-value |
| T |  | T3-T4 vs. T1-T2 | 1.313 (0.817-2.542) | | 0.1243 |
| N |  | N1-N3 vs. N0 | 1.623 (0.956-3.095) | | 0.0681 |
| Stage |  | III-IV vs. I-II | 2.551 (1.376-4.012) | | 0.0006 |
| YAP-1 |  | High vs. Low | 2.112 (1.177-3.002) | | <0.0001^*^ |
| Cox univariate analysis (RFS) | | |  |  |  |
| Variables | | Comparison | HR (95% CI) | | *P*-value |
| T |  | T3-T4 vs. T1-T2 | 1.758 (1.283-3.332) | | 0.0027 |
| N |  | N1-N3 vs. N0 | 2.538 (1.648-4.550) | | <0.0001^*^ |
| Stage |  | III-IV vs. I-II | 2.764 (1.459-4.162) | | <0.0001^*^ |
| YAP-1 |  | High vs. Low | 2.116 (1.174-2.789) | | 0.0031 |
| Cox multivariate analysis (RFS) | | |  |  |  |
| Variables | | Comparison | HR (95% CI) | | *P*-value |
| T |  | T3-T4 vs. T1-T2 | 1.228 (0.792-2.672) | | 0.0906 |
| N |  | N1-N3 vs. N0 | 1.502 (1.078-3.189) | | 0.0366 |
| Stage |  | III-IV vs. I-II | 1.989 (1.167-3.384) | | <0.0001^*^ |
| YAP-1 |  | High vs. Low | 1.719 (1.239-3.289) | | 0.0047^*^ |

**Supplement Figure S1.** RT-PCR analysis of YAP1 downstream targets and S26 mRNA expression in regorafenib-treated SNU-1079 cells.


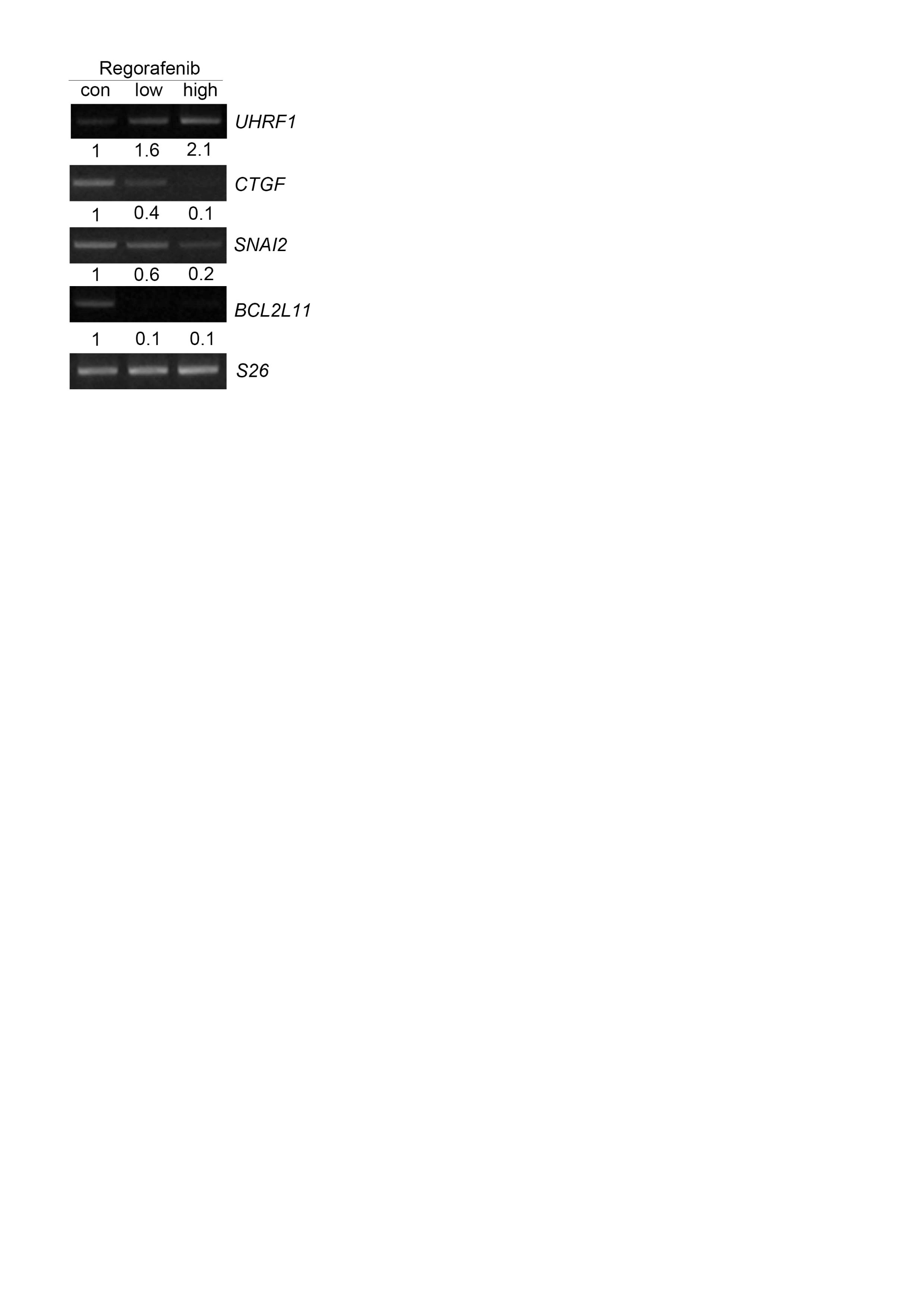


**Supplement Figure S2.** Q-PCR analysis of YAP1 downstream targets and GAPDH mRNA expression in regorafenib-treated HuCCT1 and SNU-1079 cells, respectively. **: *p*<0.01, ***: *p*<0.001.

**
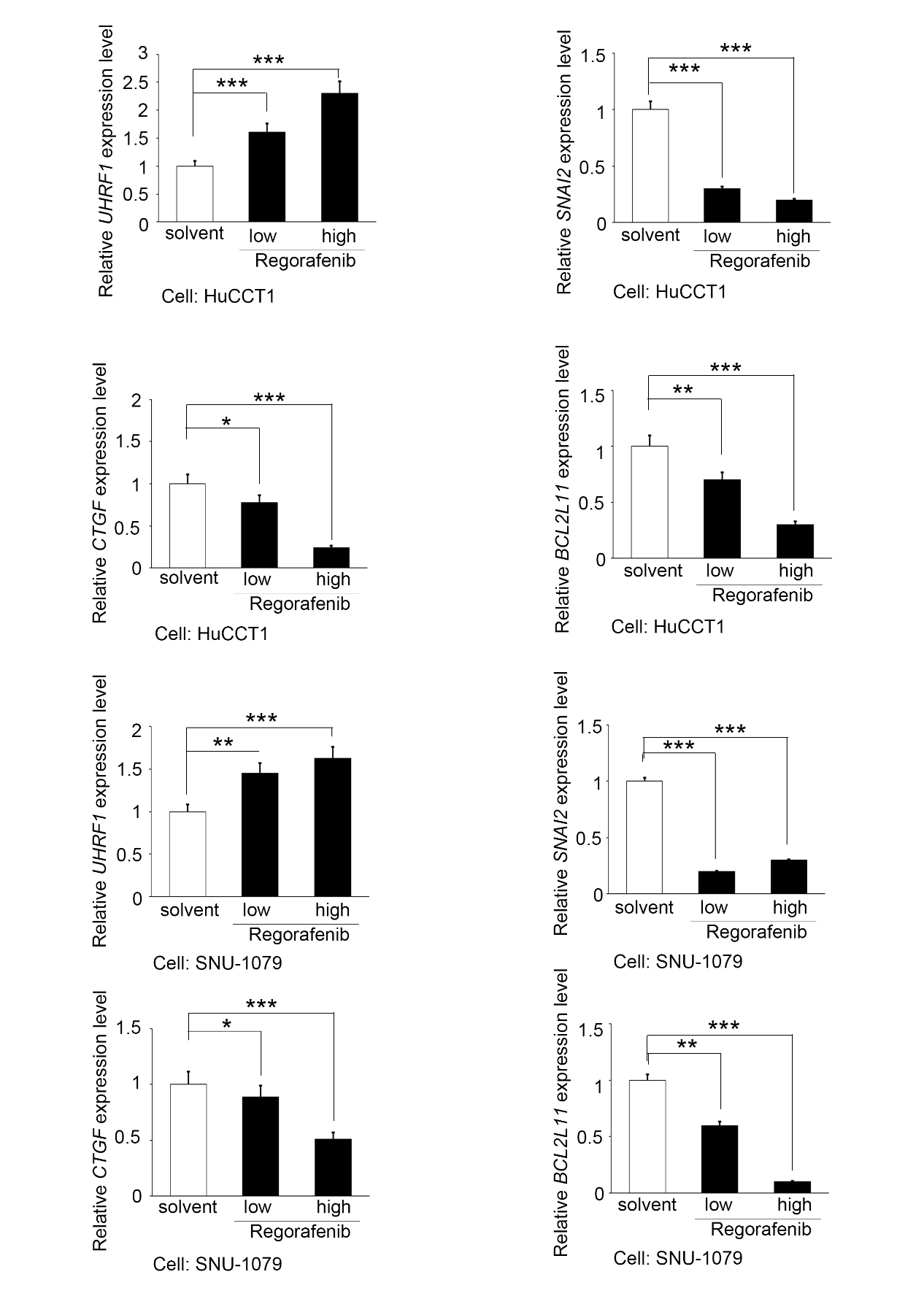
**

**Supplement Figure S3.** Q-PCR analysis of YAP1 downstream targets, *CCN1, CCNE1, CCND1, PTGS2, CDKN1B*, and GAPDH mRNA expression in regorafenib-treated HuCCT1 and SNU-1079 cells, respectively. **: *p*<0.01, ***: *p*<0.001.

**
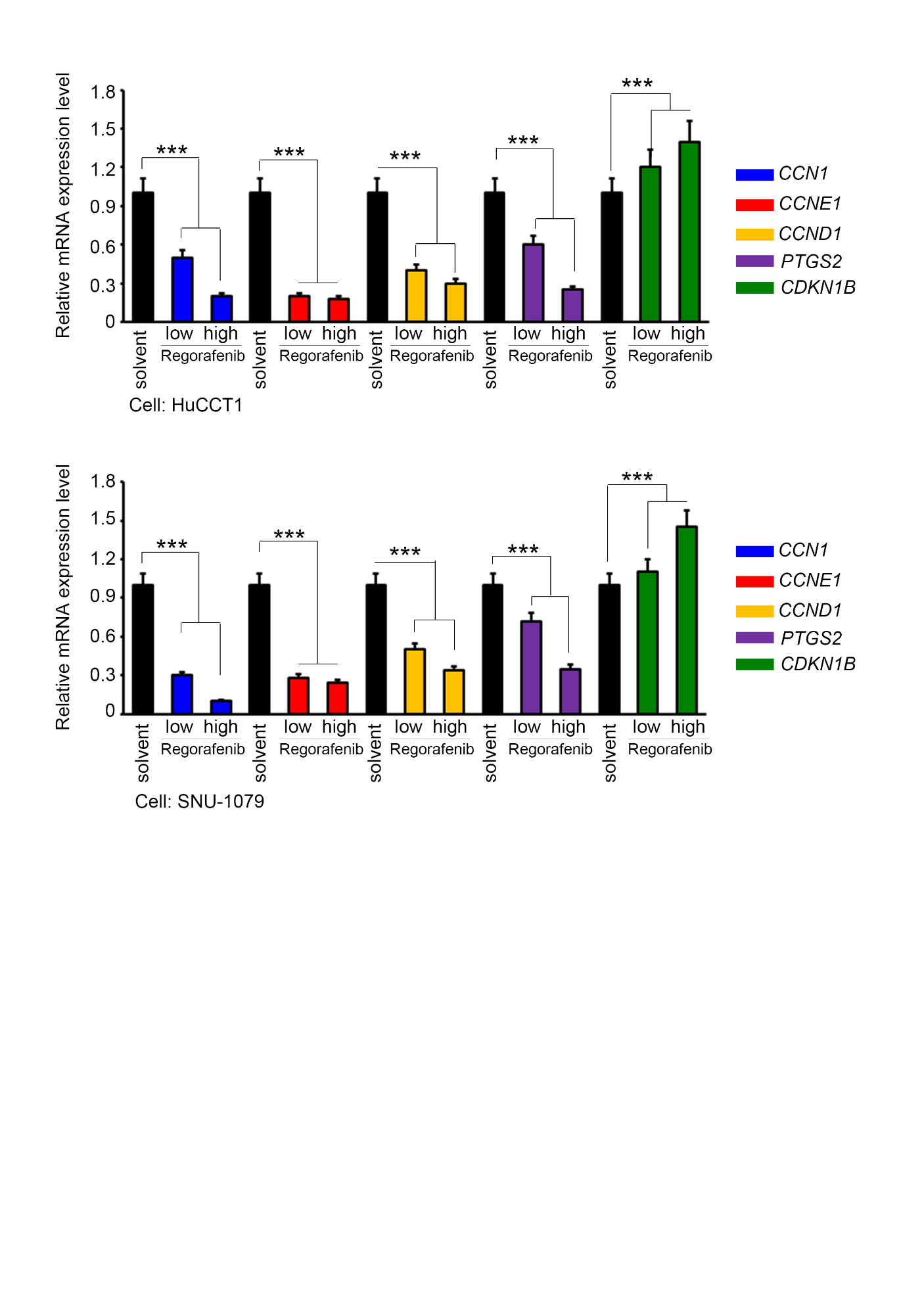
**

**Supplement Figure S4.** Western blot analysis of YAP1, p-YAP1 (Ser127 and Tyr357) , SNAI2 and CTGF levels in regorafenib-treated HuCCT1 cells.


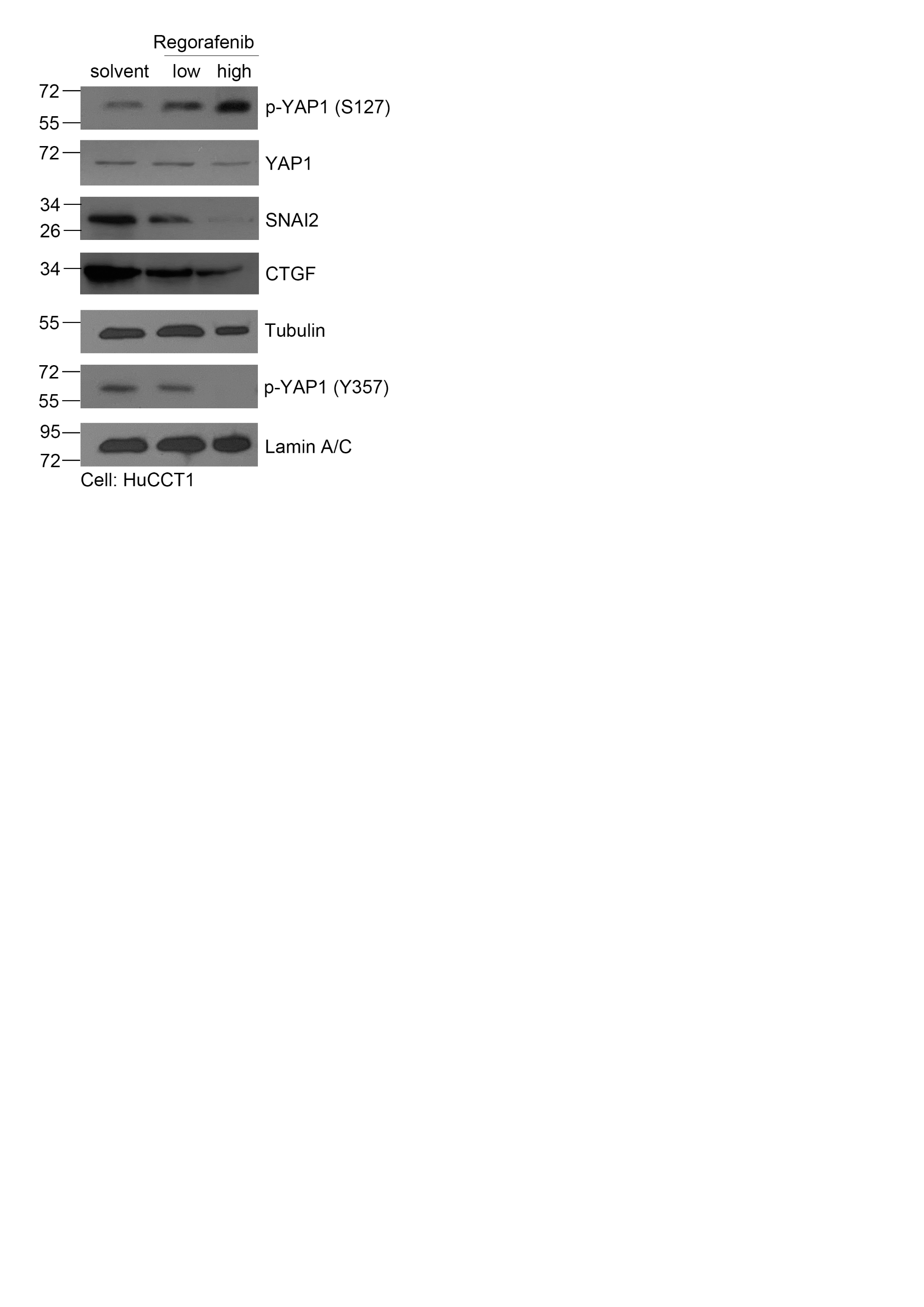


**Supplement Figure S5.** Heat map showing the expression levels of EMT markers and EMT score in a CCA cell panel; red: mesenchymal-type and green: epithelial-type.


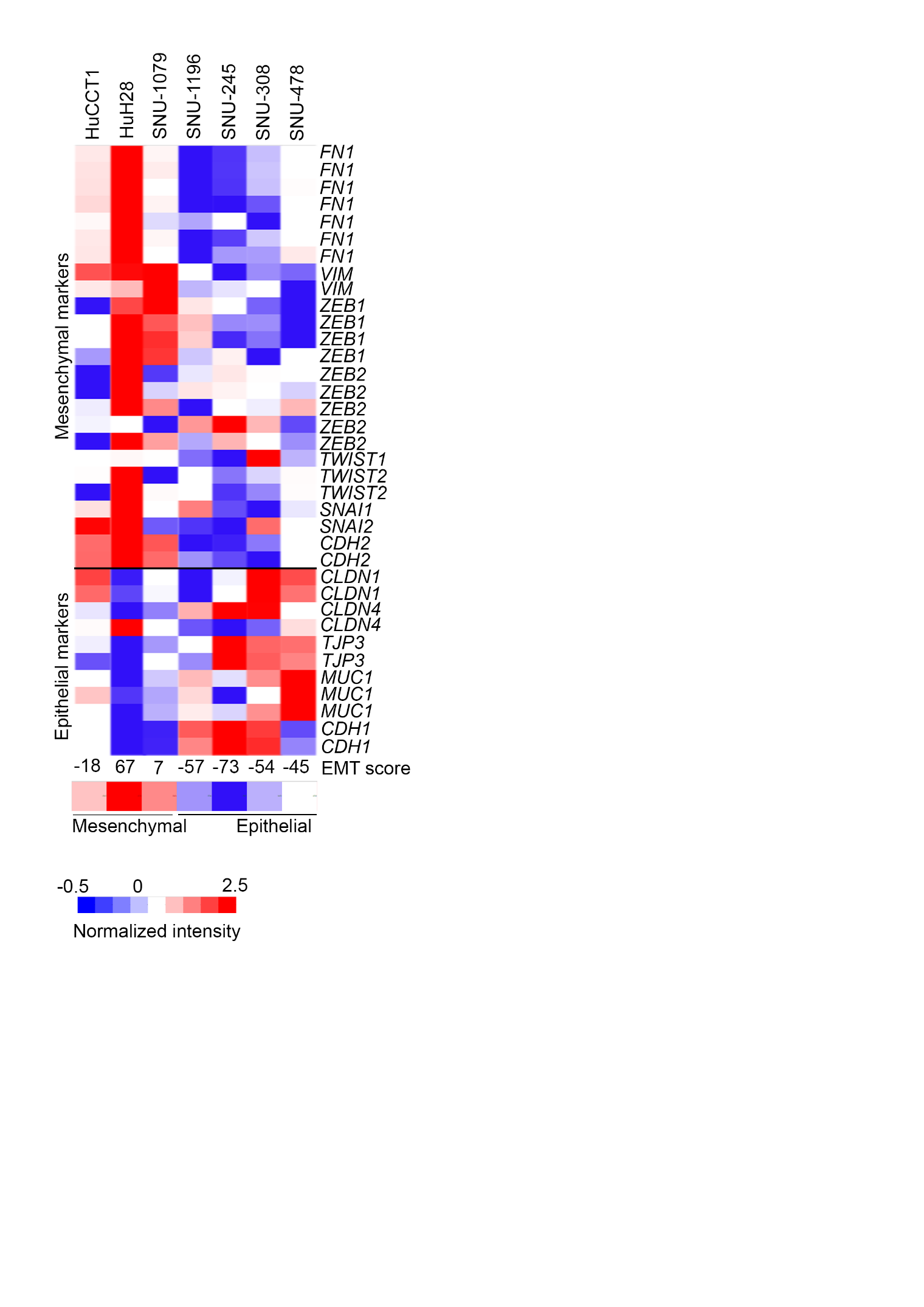


**Supplement Figure S6.** Q-PCR analysis of mesenchymal-type markers mRNA expression in regorafenib-treated SNU-1079 cells and HuCCT1 cells, respectively. ***: *p*<0.001.


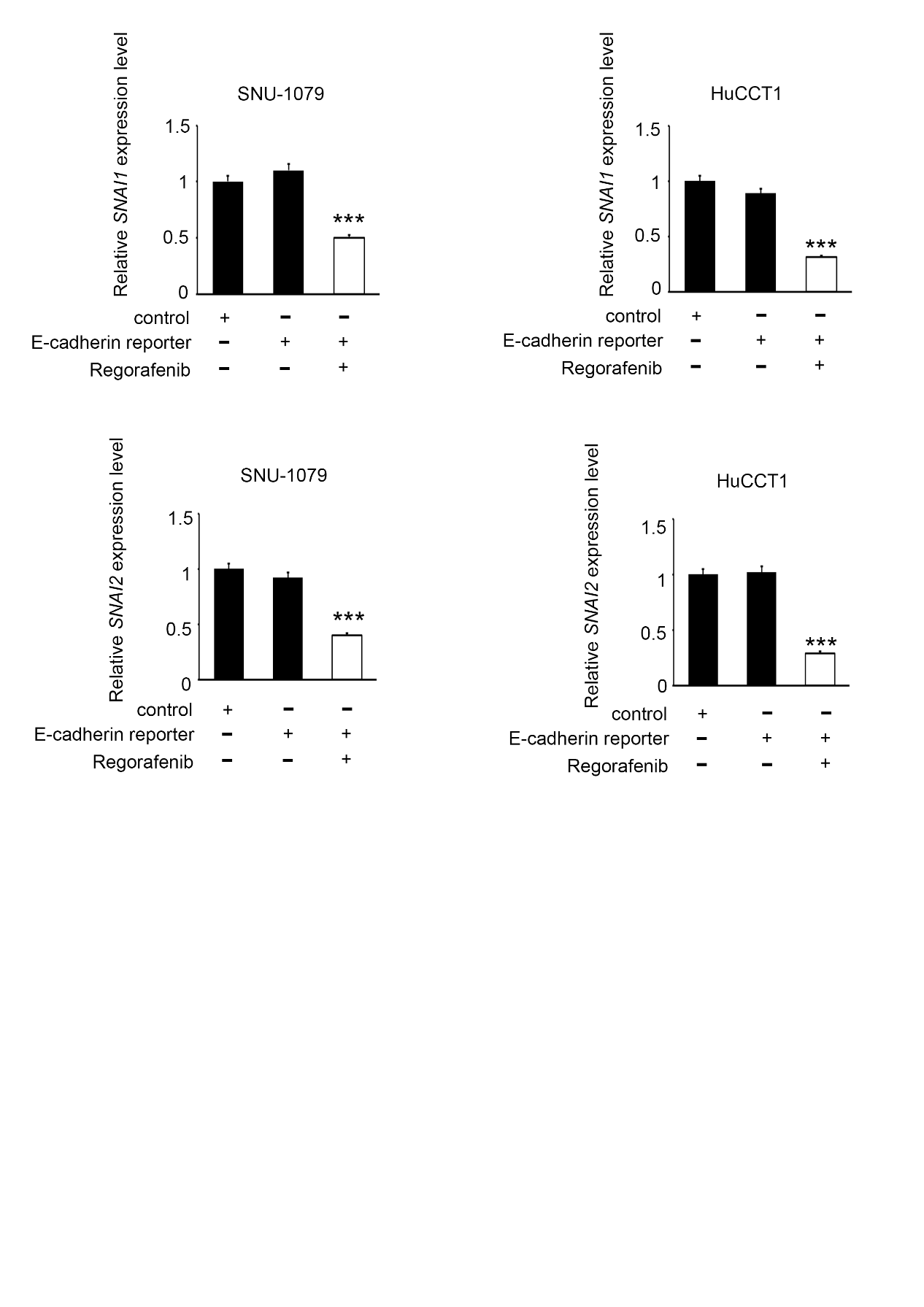


**Supplement Figure S7.** (A) Viability of SNU-1079 and HuCCT1 cells after verteporfin treatment. (B) Quantitative CK-8 activity and (C) Caspase 3 activity with or without verteporfin and regorafenib treatment in CCA cells, respectively. (D) Quantitation of migration ability of HuCCT1 and SNU-1079 cells treated with verteporfin and regorafenib, respectively. *: *p*<0.05, **: *p*<0.01, ***: *p*<0.001.

**
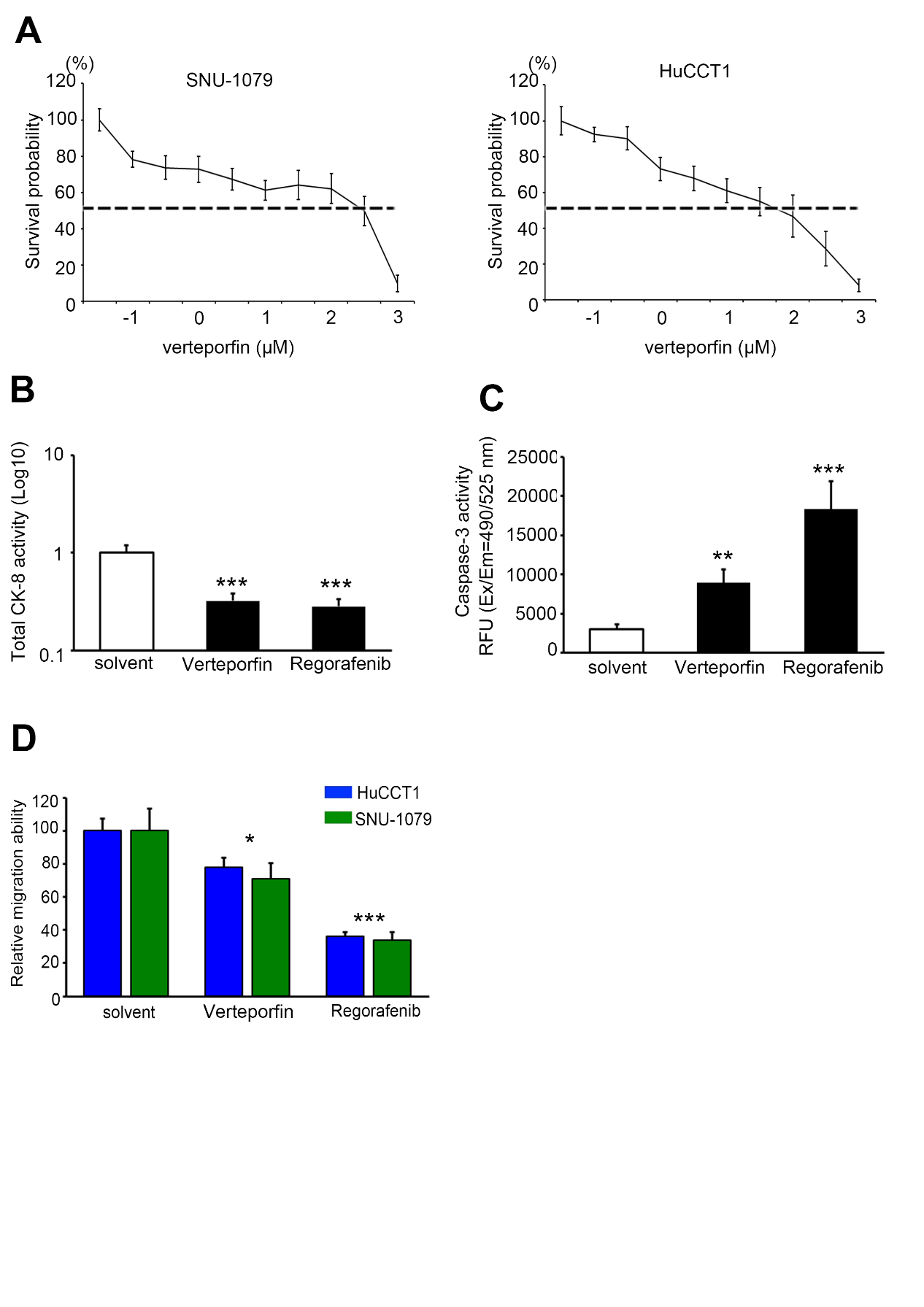
**

**Supplement Figure S8.** Correlation between YAP1 with UHRF1, BCL2L11 and CDH1 mRNA levels in TCGA_CHOL clinical patients (ρ = 0.24, *p* = 0.15; ρ = 0.35, *p* = 0.0024; ρ = 0.27, *p* = 0.11, respectively).

**
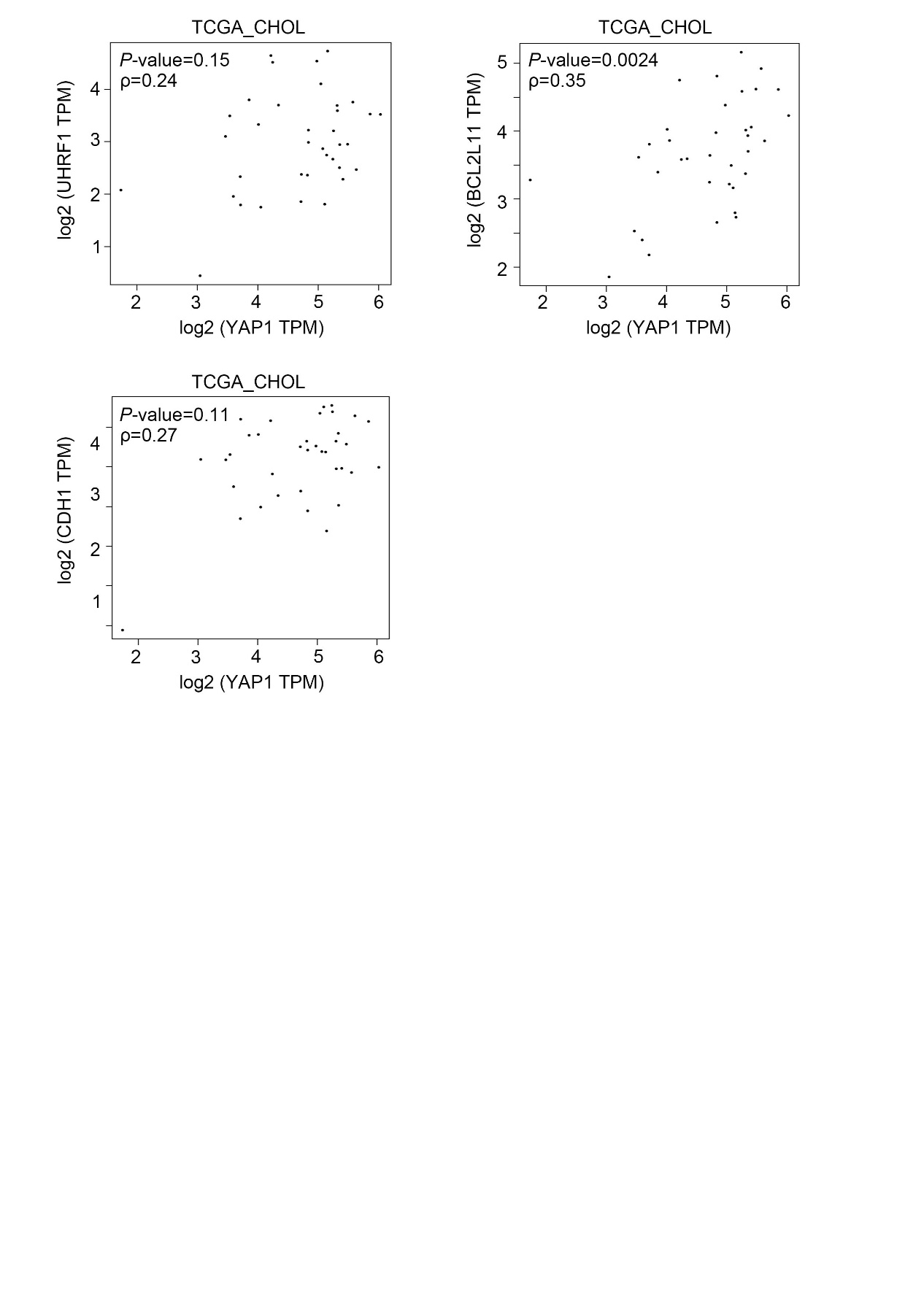
**

**Supplement Figure S9.** (A) Endogenous RNA levels of YAP1 downstream genes in a cholangiocarcinoma cell panel. (B) qRT-PCR of AREG levels in a CCA cell panel (human cholangiocyte primary cell, MMNK-1, and HuCCT1).


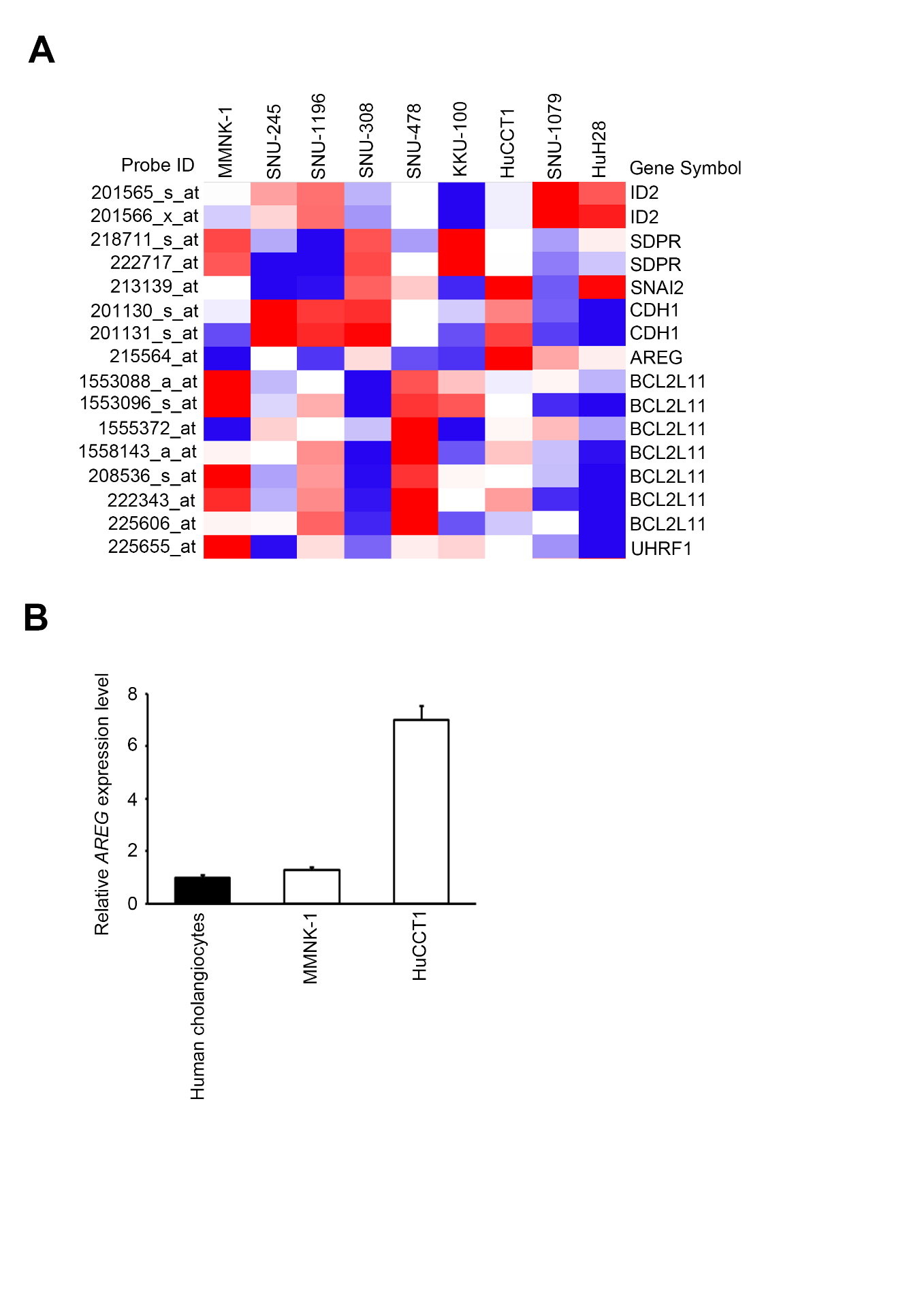


**Supplement Figure S10.** qRT-PCR analysis of AREG mRNA expression (A) in HuCCT1 or SNU-1079 cells treated with the YAP1 inhibitor verteporfin (1μM and 10 μM) and YAP1 shRNAs, respectively. (B) in HuCCT1 or SNU-1079 cells treated with regorafenib (10 μM) or combined with overexpression plasmids for YAP1, respectively. *: *p*<0.05, **: *p*<0.01, ***: *p*<0.001.

**
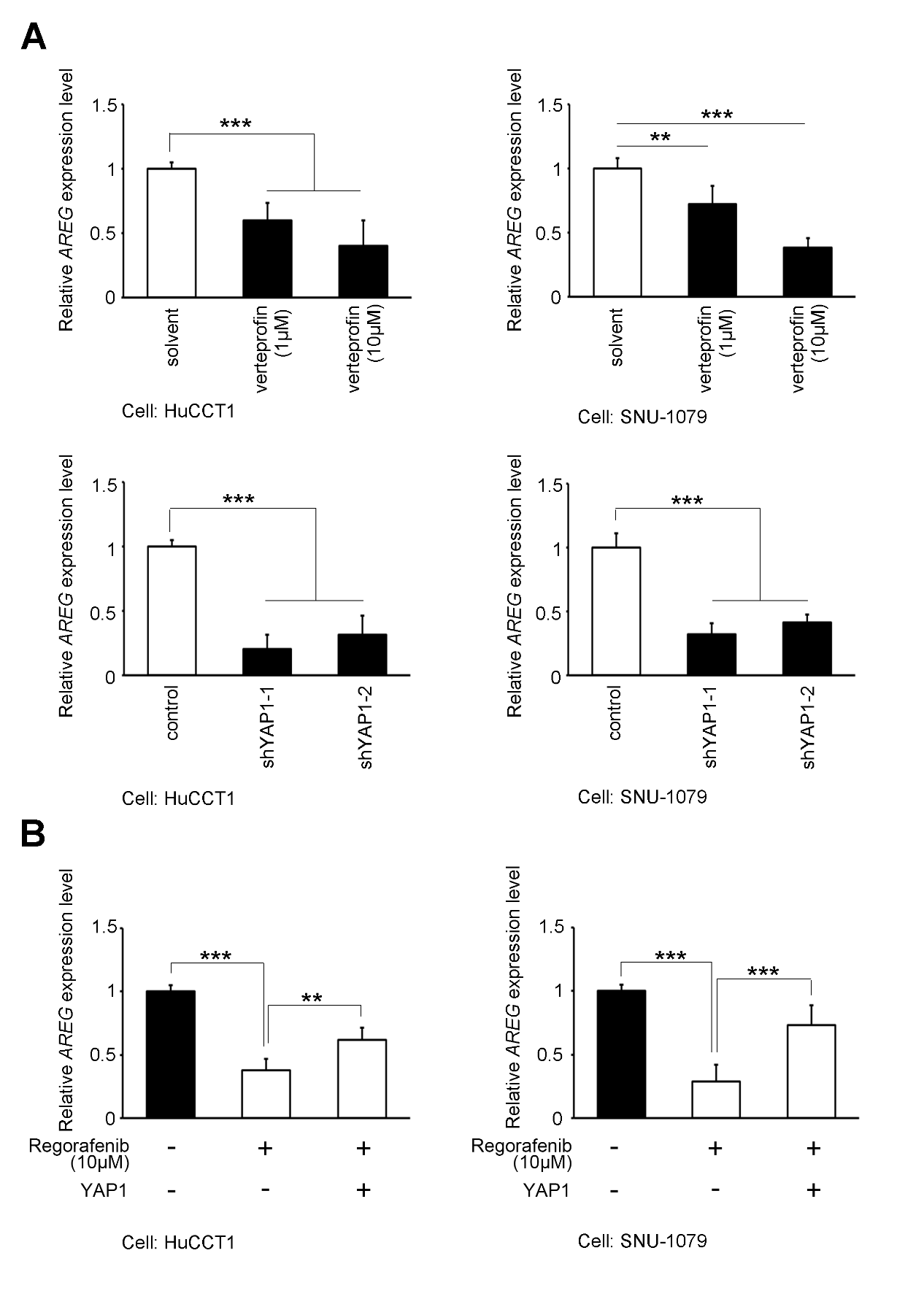
**

**Supplement Figure S11.** Western blots showing YAP1 protein levels after treatment of HuCCT1 and SNU-1079 cells with anti-AREG antibodies.


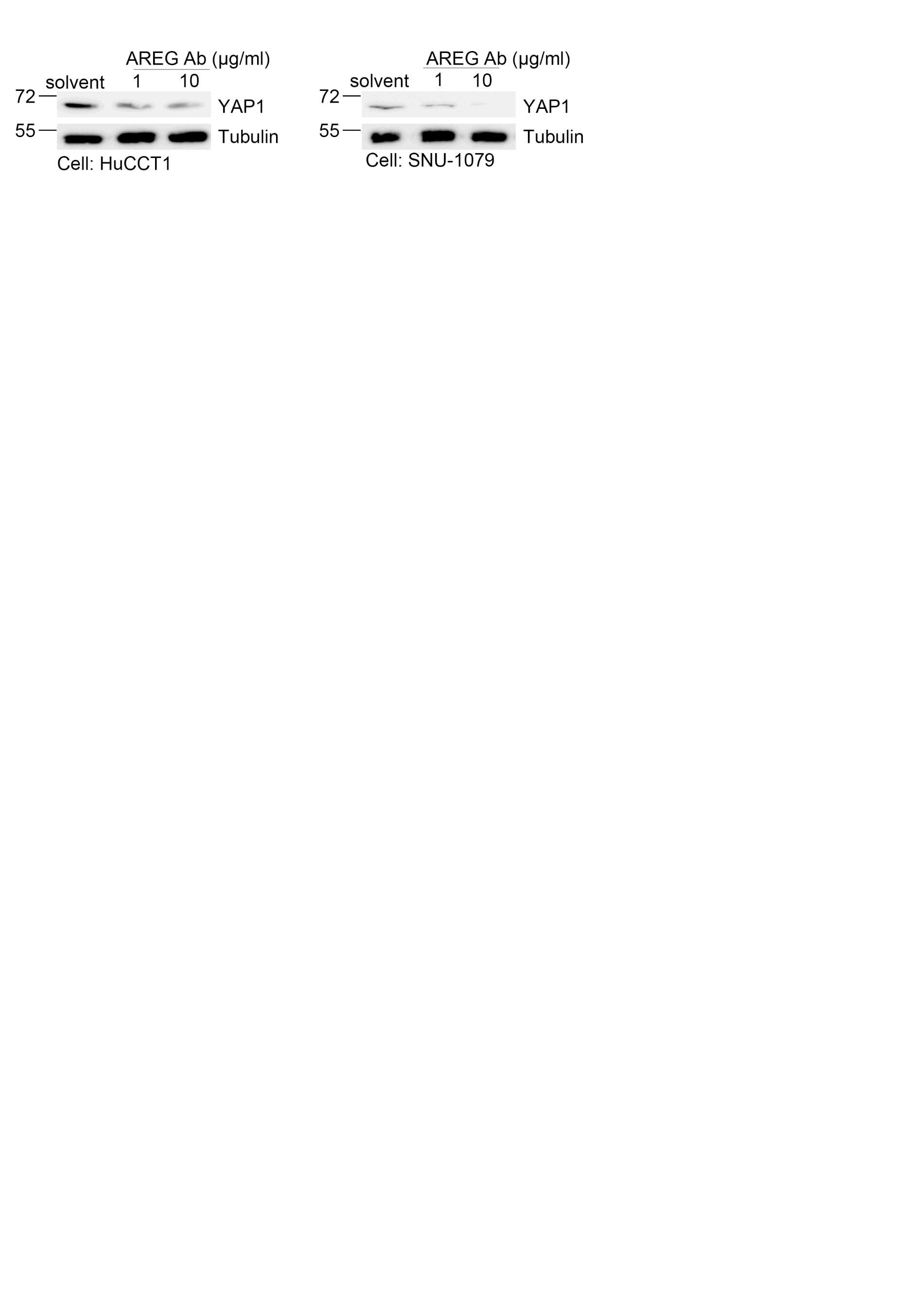


**Supplement Figure S12.** Representative IHC staining intensity of p-YAP1 (Y357), p-YAP1 (S127), and AREG protein in CCA tissues. Scale bar: 50 µm.


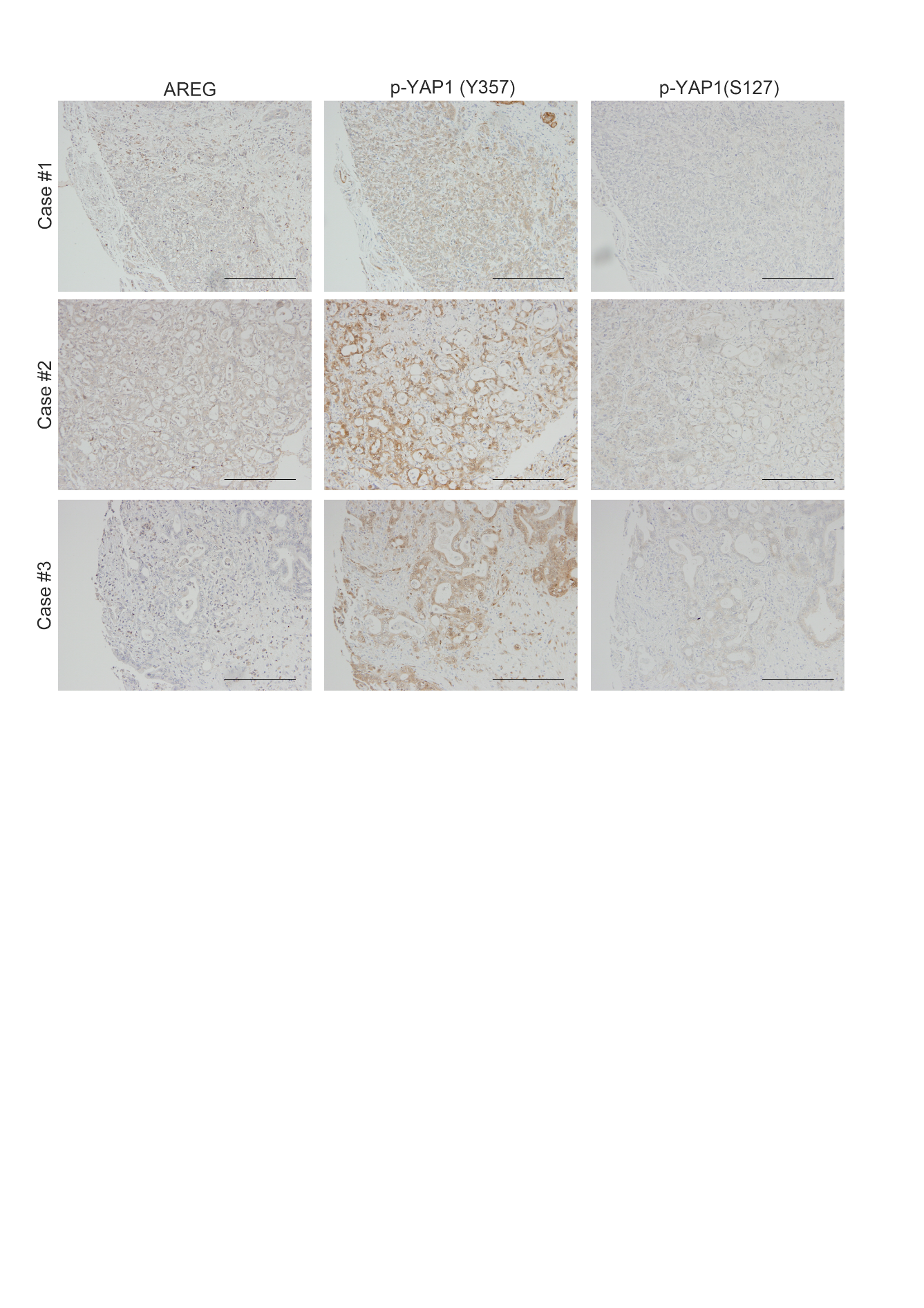


**Supplement Figure S13.** Western blot analysis of YAP1, ALOX5, and tubulin levels in a CCA cell panel (human cholangiocyte primary cell, MMNK-1, and HuCCT1).


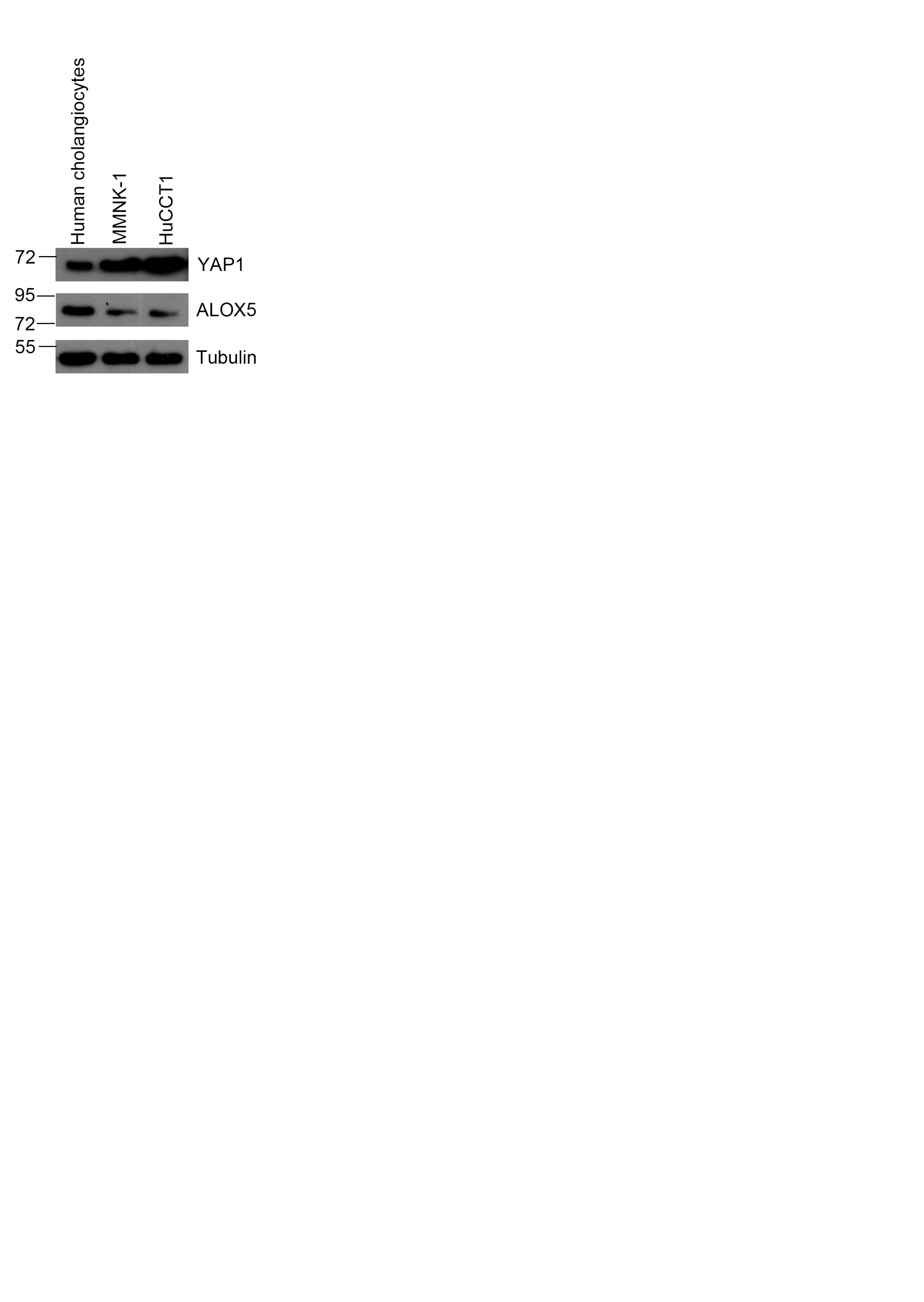

Supplement: Supplementary file 1 — Supplementary profile [file 41419_2022_4816_MOESM1_ESM.docx]
