# Supplementary material for: Regorafenib inhibits epithelial-mesenchymal transition and suppresses cholangiocarcinoma metastasis via YAP1-AREG axis
Source: Cell Death Dis. 2022 Apr 21;13(4):391. doi: 10.1038/s41419-022-04816-7 (PMC9023529; doi:10.1038/s41419-022-04816-7)
Supplement: Supplementary file 2 — Author contributions [file 41419_2022_4816_MOESM2_ESM.docx]

**Author contributions**

**Conception and design:** Yu-Chan Chang, Ming-Huang Chen, Michael Hsiao

**Development of methodology:** Yu-Chan Chang, Chien-Hsiu Li, Ming-Hsien Chan, Chun-Nan Yeh, Ming-Huang Chen

**Acquisition of data (provided animals, acquired and managed patients, provided facilities, etc.):** Yu-Chan Chang, Chien-Hsiu Li, Ming-Hsien Chan, Chun-Nan Yeh, Ming-Huang Chen, Michael Hsiao

**Analysis and interpretation of data (e.g., statistical analysis, biostatistics, computational analysis):** Yu-Chan Chang, Ming-Huang Chen

**Writing, review, and/or revision of the manuscript:** Yu-Chan Chang, Chien-Hsiu Li, Ming-Hsien Chan, Chun-Nan Yeh, Ming-Huang Chen, Michael Hsiao

**Administrative, technical, or material support (i.e., reporting or organizing data, constricting databases):** Chun-Nan Yeh, Ming-Huang Chen

**Study supervision:** Michael Hsiao
