# Supplementary material for: Regorafenib inhibits epithelial-mesenchymal transition and suppresses cholangiocarcinoma metastasis via YAP1-AREG axis
Source: Cell Death Dis. 2022 Apr 21;13(4):391. doi: 10.1038/s41419-022-04816-7 (PMC9023529; doi:10.1038/s41419-022-04816-7)
Supplement: Supplementary file 4 — Supplemental Table S1 [file 41419_2022_4816_MOESM4_ESM.pdf]

Probe Set ID	Regorafenib low dose v.s. solvent (fold-change)
206924_at	-2.8053937
204614_at	-3.1492376
204014_at	-1.3948965
208893_s_at	-2.0481815
208891_at	-2.0158043
208892_s_at	-1.9753656
226034_at	-1.3335123
238689_at	-1.4022322
230398_at	-1.3814888
202126_at	-0.6745806
236835_at	-0.64784336
203889_at	-1.3116541
208025_s_at	-0.9715986
227529_s_at	-2.1145353
202068_s_at	-0.7190161
230493_at	-2.9847832
234700_s_at	-1.5142913
204015_s_at	-1.3367205
206170_at	-0.95517635
232291_at	-1.6083956
243409_at	-1.0221124
237411_at	-1.5205169
212444_at	-0.6651106
242396_at	-0.41300488
1555673_at	-1.8683424
1563057_at	-0.45380497
230710_at	0.19733524
237435_at	-0.8108702
207345_at	-0.35540915
220468_at	-1.2179089
227530_at	-2.0930686
206029_at	-0.3515272
228923_at	-1.2180676
203108_at	-0.6769619
235988_at	-1.1642437
213895_at	-1.5621033
205844_at	0.02322197
205067_at	-1.2834225
201860_s_at	-1.3309364
231798_at	-1.440568
239410_at	-0.8352585
209909_s_at	-0.25515366
227486_at	-1.7366161
202067_s_at	-0.8098459
235086_at	-1.2678003

200769_s_at	-1.4484854
229332_at	-0.5563226
225673_at	-0.38287163
219795_at	-1.2140465
219476_at	-0.17140293
227475_at	-0.34095
235251_at	-1.0793362
39402_at	-1.1702337
204602_at	-0.51068115
217655_at	-1.2531276
243871_at	-0.3510728
237166_at	-0.6509199
236480_at	-0.24702072
201109_s_at	-0.505991
205330_at	-0.620903
205680_at	-1.0367231
209101_at	-1.3403306
41469_at	-1.0341725
228776_at	-1.4185991
220407_s_at	-0.34792233
228573_at	-1.6076317
239463_at	-0.53569365
226847_at	-0.0673666
205490_x_at	-0.922843
203691_at	-1.042757
230250_at	-0.5186224
200983_x_at	-0.12202263
210517_s_at	-1.7926927
234219_at	-1.3684473
229092_at	-0.38313007
232290_at	-0.78185606
242005_at	-1.4689112
209765_at	-0.3943143
227792_at	-0.5786381
1557285_at	-1.5236793
236893_at	-1.2029414
204794_at	-1.2576284
229103_at	-0.33968163
227335_at	-0.6405692
228748_at	-0.46327305
239973_at	-0.5671997
203833_s_at	-0.21478939
1556385_at	-0.9537735
219026_s_at	-0.8541455
211518_s_at	-0.053554535
232344_at	-0.4801054

216623_x_at	-0.39777565
1561418_at	-0.6385231
214803_at	0.2208848
227514_at	-0.60382366
243888_at	-0.78204155
202934_at	-1.0563164
200768_s_at	-0.9861355
1557754_at	-0.8223171
201250_s_at	-0.79738903
204475_at	-1.8269005
220289_s_at	-0.5841565
204948_s_at	-0.10529232
225524_at	-1.5278511
236489_at	-0.9200783
200984_s_at	-0.13032913
229371_at	-0.8261218
1554020_at	-1.2240429
205302_at	-0.9605894
230369_at	-0.06568766
1555976_s_at	-0.89618397
202431_s_at	-1.1036978
210017_at	-0.5301485
217999_s_at	-0.93993235
229151_at	-1.5168743
235168_at	-0.3799281
219334_s_at	-0.94739866
201703_s_at	-0.57317257
1558280_s_at	-1.3730955
215243_s_at	-0.9762931
209598_at	0.54227257
204774_at	-0.4457059
237252_at	-0.60675573
1568619_s_at	-0.67936134
1564906_at	-0.86795664
219522_at	-0.6503792
221538_s_at	-0.106425285
229347_at	-0.59876966
217173_s_at	-0.69307137
203726_s_at	-0.13769054
228977_at	-0.9104085
226064_s_at	-0.37735367
242979_at	0.024742603
230127_at	-0.68417454
36711_at	-1.3314409
234699_at	-1.1882563
200985_s_at	-0.102458954

227368_at	-0.10786772
205264_at	-1.1534243
1558549_s_at	0.085100174
226484_at	-0.08706522
212942_s_at	-0.18103313
230112_at	-1.1692896
210026_s_at	-0.40973186
213069_at	-0.057282448
206685_at	-0.29401588
219395_at	-0.6052532
204011_at	-1.3008575
218095_s_at	-0.31307125
213310_at	-0.9129529
227140_at	-0.23457909
204420_at	-1.2746649
227954_at	-0.6550579
222305_at	-0.8235564
226825_s_at	-0.32330227
209260_at	-0.8771639
202815_s_at	-0.52817345
205596_s_at	-1.0851154
225646_at	-0.6752558
205014_at	-0.12061262
237466_s_at	-0.67455626
230820_at	-1.0367012
219181_at	-0.75541735
228865_at	-0.19362736
205600_x_at	-0.8317332
39248_at	-0.6227608
224917_at	0.03730774
230345_at	-0.4624405
1560071_a_at	-0.6081042
217833_at	-0.3676548
210511_s_at	-0.18320274
238243_at	-0.7556238
226237_at	0.44149685
208016_s_at	-1.0387917
223470_at	-0.33148766
1553994_at	-0.8879452
205856_at	-1.2793381
202628_s_at	-1.1902285
204363_at	-0.4026785
1555847_a_at	0.16556072
222108_at	0.04432392
225120_at	-0.44638538
205466_s_at	-0.8713455

221840_at	-0.6641903
244503_at	-0.6402345
203887_s_at	-0.5264702
203834_s_at	-0.42197847
227978_s_at	-0.12818003
205357_s_at	-1.146492
238551_at	-0.7140651
230061_at	1.0498295
231775_at	-1.2062426
219836_at	-0.040929794
226987_at	-0.53069925
236622_at	-0.3165083
210793_s_at	-1.1232452
228280_at	-0.83938026
203888_at	-0.5273142
201667_at	-0.23255014
215034_s_at	0.057670593
33323_r_at	-0.705492
213913_s_at	-0.86953545
200020_at	-0.40400124
219500_at	-0.689693
1553947_at	-0.7564292
212753_at	-0.44947577
209446_s_at	-0.68062973
224733_at	-0.47208405
225647_s_at	-0.49142742
242539_at	-1.371891
227604_at	-0.60232687
204037_at	-0.5005207
1553995_a_at	-0.864316
205465_x_at	-0.86797476
236115_at	-0.31815195
235247_at	0.7962694
210621_s_at	-0.5921087
204998_s_at	-0.80390406
228987_at	-0.19567299
242450_at	-0.97119045
1552283_s_at	-0.835608
223690_at	-0.21200562
219856_at	-0.04203415
235497_at	-1.0133915
221873_at	-0.68288517
233488_at	-1.3935304
230300_at	-0.67487574
228353_x_at	-0.9775195
226860_at	0.14054585

225239_at	0.023989677
233487_s_at	-0.7205324
232667_at	-0.6416831
230998_at	-0.6164355
224783_at	-0.44500637
244669_at	-0.9049363
226775_at	-0.4894867
224558_s_at	-0.29742146
223707_at	-0.7171707
33322_i_at	-0.61273956
224219_s_at	-0.17333078
1562367_at	-0.77156115
220102_at	-0.2739601
209457_at	-1.5881948
230598_at	-0.024343014
201249_at	-0.87670755
212040_at	-0.2674141
219710_at	-0.30044413
223834_at	-2.438192
204036_at	-0.55048084
201585_s_at	-0.9690771
204012_s_at	-0.6753888
225511_at	0.67806625
236892_s_at	-0.97448206
1552648_a_at	-1.1738405
1569582_at	-1.1280563
223314_at	-0.6280308
210018_x_at	-0.6201792
235661_at	-0.59682226
236129_at	0.1022892
205032_at	-0.950264
205366_s_at	-1.09519
209387_s_at	0
212168_at	-0.6157532
205193_at	-1.0848413
217028_at	-0.20053768
238050_at	-1.0150414
205846_at	-0.59421444
229691_at	-0.1991725
210248_at	-1.0190897
201325_s_at	-0.7608137
1555789_s_at	-0.35156727
226756_at	-0.03297615
1553108_at	-0.4876814
238909_at	-0.7774396
228523_at	-0.5878563

204956_at	-0.6890278
223081_at	-0.39185238
225842_at	-0.56215286
1562048_at	-0.5088644
1554239_s_at	-0.20832682
201702_s_at	-0.33716345
238933_at	-0.55577135
222906_at	-0.90497494
225978_at	-0.54595137
227164_at	-0.83741283
241726_at	-0.4999013
214894_x_at	-0.37514687
219371_s_at	-0.8076649
213094_at	-0.57997227
205601_s_at	-0.8919468
237097_at	-1.0205665
218590_at	-1.0340233
207358_x_at	-0.34297562
200685_at	-0.42298794
226762_at	-0.20206165
227617_at	-1.2281885
226129_at	-0.3115406
229126_at	0.32654476
202814_s_at	-0.34427166
225733_at	-0.4043436
225805_at	-0.56458855
228820_at	-0.19591045
202856_s_at	-1.0169544
1555963_x_at	0.41998196
210473_s_at	-0.50689507
243431_at	-0.8607063
239331_at	-1.5364442
1556037_s_at	-0.8634176
209386_at	0.06713104
208634_s_at	-0.3478279
235692_at	-0.37850904
235014_at	-0.10625839
208309_s_at	-0.5705881
227314_at	-0.975893
238529_at	0
220459_at	0.105091095
238965_at	-0.69116926
212170_at	-0.40388966
243426_at	-0.49173355
1555355_a_at	-1.005827
227593_at	-0.17542696

211919_s_at	-0.28961277
218691_s_at	-0.1795206
231892_at	-0.21714401
227847_at	-0.41879177
235245_at	0.12603235
219596_at	-0.2554884
224920_x_at	-0.18279839
236769_at	-0.24157763
203580_s_at	-0.96357346
202855_s_at	-1.1573896
228397_at	-0.04310417
1564796_at	-0.8944168
227993_at	-0.74997663
205527_s_at	-0.6213865
219369_s_at	-0.8926139
218016_s_at	-0.7487879
209431_s_at	0.04421234
235882_at	-0.8236966
203578_s_at	-1.0738392
213684_s_at	-0.33595324
213899_at	-0.69300795
227044_at	-0.11146641
202436_s_at	-1.0234876
203243_s_at	-0.35884857
204595_s_at	-0.60241795
217651_at	-0.76835203
227337_at	-0.6953049
1554486_a_at	-0.617198
200753_x_at	-0.6380644
210143_at	-0.22670746
243417_at	0.14051867
225655_at	-0.27794647
1559964_at	-0.44860363
209119_x_at	-0.31927538
1555996_s_at	-0.9780836
1553708_at	0.5556216
209201_x_at	-0.31252003
209125_at	-0.51292896
231984_at	-0.9035654
221470_s_at	-1.3251643
230296_at	-0.82895947
1569003_at	-0.24191856
202241_at	-1.0566139
227517_s_at	-0.7130375
202206_at	-0.24393177
212693_at	-0.7538047

207517_at	-0.60964966
227095_at	-0.12448406
201129_at	-0.4976492
229811_at	-0.37760305
230051_at	-0.8045955
227448_at	-0.3824234
224602_at	-0.017220497
201324_at	-0.6924467
230746_s_at	-0.43883324
205258_at	-0.11996937
220961_s_at	-0.54400206
235563_at	-0.5899091
204597_x_at	-0.5352359
227578_at	-0.1820054
240189_at	-0.8179617
222480_at	-0.49985504
222335_at	-0.84111357
244647_at	-0.33956337
241529_at	-1.142117
220915_s_at	-0.3679099
238462_at	-0.5043993
219937_at	-0.47433615
204038_s_at	-0.7051754
204718_at	-0.24832153
214587_at	0.050691605
212043_at	-0.060456276
227458_at	-2.442151
214014_at	-0.69230366
206079_at	-0.5719032
203579_s_at	-1.1242595
243612_at	-0.952548
203643_at	-0.1413045
1553106_at	-0.4964118
238065_at	-0.6582875
225562_at	-0.87989664
232504_at	-0.10656643
206382_s_at	-0.22630596
228121_at	0.2942667
228066_at	-0.6046257
1552822_at	-0.41373444
1566557_at	0.49489594
211576_s_at	-0.38630867
209567_at	-0.8466282
203757_s_at	-0.59244204
233589_x_at	-0.42520285
236106_at	-0.4530115

239730_at	-0.522984
235729_at	-0.19428158
205513_at	-0.6032896
226413_at	-0.34527636
219987_at	-0.8734374
221799_at	-0.45605755
241773_at	-0.9094653
204686_at	0.25002766
1561042_at	-0.8152356
228191_at	-0.81335354
218723_s_at	-0.5529313
212864_at	-0.40371132
236632_at	-0.4196396
212412_at	-0.32624817
228039_at	-0.49615622
219491_at	-0.55920124
222589_at	-0.59046936
212636_at	-0.83229256
232431_at	-0.60861754
1555920_at	-0.72035885
213718_at	-0.5262966
211985_s_at	-0.6423235
211742_s_at	-0.14224005
212925_at	0.023981094
202936_s_at	-1.0329304
209884_s_at	-0.56062317
202935_s_at	-1.2067041
203699_s_at	-0.06500578
222006_at	-0.6362486
214774_x_at	-0.36413097
216685_s_at	-0.6180973
213390_at	0.001040459
228343_at	-0.6627078
238750_at	1.1207352
218544_s_at	-0.9597502
229801_at	-0.9330187
227749_at	-0.33182335
242866_x_at	-0.62040615
201675_at	-0.33867168
239914_at	-0.67806196
226347_at	-0.58071136
232020_at	-0.8935833
202208_s_at	-0.4376011
215073_s_at	-0.24418831
244523_at	-0.626461
203139_at	0.7844553

212114_at	0.00124073
235060_at	-0.5925541
214499_s_at	-0.71442413
211363_s_at	-0.67997074
211090_s_at	-0.7963877
238299_at	-0.80785227
241371_at	-1.0422978
202207_at	-0.15174007
206558_at	-0.5063591
206429_at	-0.8797121
206919_at	-0.7523737
1559006_at	-0.3423109
227977_at	-0.13207912
242439_s_at	-0.67479324
229964_at	1.3389359
229054_at	0.047890663
224624_at	-0.47121525
219620_x_at	-0.31019402
202516_s_at	-0.38325787
228904_at	-0.7440758
202677_at	-0.41202927
238044_at	-0.5102663
220907_at	-0.6494942
204222_s_at	-0.50995064
201110_s_at	-0.15714264
227272_at	-0.642148
226638_at	-0.011610031
204330_s_at	-0.6282368
202437_s_at	-0.7953119
211984_at	-0.5734806
226431_at	-0.7180295
213338_at	-0.627059
231199_at	-1.0470295
206972_s_at	0.1680603
225897_at	0.7387104
228287_at	-0.55274343
238587_at	-0.7307291
226206_at	-0.88251495
230676_s_at	0
227477_at	-0.6765318
55093_at	-0.44980288
239050_s_at	-0.483675
203180_at	-0.792078
228955_at	-0.70445776
226734_at	-0.34792137
201196_s_at	-0.7769203

214447_at	-0.6824274
208712_at	-0.9450216
214255_at	0.2756729
204337_at	-0.65709066
1553300_a_at	-0.2479043
207907_at	-0.31649303
205284_at	-1.0745826
235760_at	-0.73492
225342_at	-0.2900648
221688_s_at	-0.52586174
219539_at	-0.29520416
235392_at	-0.4188323
205015_s_at	-0.8320813
210643_at	-0.5770507
215108_x_at	-0.17677903
212012_at	-0.07978916
227263_at	-0.17071342
239392_s_at	-0.6486063
226350_at	0.18981266
242162_at	-0.711215
231228_at	-0.29708147
201586_s_at	-0.7167988
215222_x_at	-0.39409924
211657_at	-0.3080821
203286_at	-0.16500854
222265_at	-0.5622449
232361_s_at	-0.52150583
201487_at	-0.09530735
236937_at	-0.45230818
206926_s_at	-0.78962374
233364_s_at	-1.1414914
224785_at	-0.35058975
232360_at	-0.3258686
214011_s_at	-0.86320114
203821_at	-1.350915
212107_s_at	-0.33209276
218641_at	-0.837028
229899_s_at	-0.95311165
242273_at	-0.48297405
219515_at	-0.192204
200931_s_at	-0.31009293
204926_at	0.023715973
209121_x_at	-0.16216373
215033_at	0
203242_s_at	-0.38310528
228762_at	-0.16674328

223038_s_at	-0.71401596
242538_at	-0.7724719
225569_at	-0.8478832
210656_at	-0.52838135
223541_at	-1.0043154
38037_at	-1.4444342
242521_at	-1.124095
235606_at	1.0179553
202917_s_at	-1.619019
216870_x_at	-1.1736984
233078_at	-1.0144405
228043_at	-1.1233487
1552546_a_at	-1.1488509
201041_s_at	-1.4594193
235020_at	-1.0718651
200796_s_at	-1.4340243
206108_s_at	-1.1385117
214099_s_at	-1.0459971
224480_s_at	-1.6549101
218000_s_at	-1.2037349
230267_at	-1.0664716
223533_at	-1.1673808
211756_at	-1.6081724
1555137_a_at	-1.1279273
218605_at	-1.0939131
1552658_a_at	-1.6810322
202613_at	-1.0091476
232113_at	-1.0683246
201694_s_at	-1.7998023
1559139_at	-1.2242103
219131_at	-1.0155559
235236_at	-1.1037712
212838_at	-1.1321144
1557810_at	-1.2890286
236907_at	-1.0719967
214056_at	-1.0076237
200798_x_at	-1.2501192
227559_at	-1.0321498
223413_s_at	-1.0071096
242905_at	-1.0076442
204823_at	-1.4106388
242963_at	-1.0558681
244546_at	-1.221456
227345_at	-1.2255025
221020_s_at	-1.0124159
242116_x_at	-1.0463467

212434_at	-1.0458832
1560318_at	-1.0046699
203068_at	-1.089355
219969_at	-1.092627
227404_s_at	-1.0378823
225381_at	-1.20018
225763_at	1.4467602
214975_s_at	-1.0471745
218647_s_at	-1.0922623
221011_s_at	1.1589613
237110_at	-1.0618501
231863_at	-1.1177773
215111_s_at	-1.0024481
209803_s_at	-1.0059118
203824_at	1.0727139
1554785_at	-1.0607061
239835_at	-1.8922944
228640_at	1.1220932
201466_s_at	-1.4007869
232105_at	1.0660558
213281_at	-1.2076082
219768_at	1.0378213
223204_at	1.1843295
239430_at	1.0895844
213317_at	1.2908783
224823_at	1.0995798
223709_s_at	1.2550507
213456_at	1.1676736
222073_at	1.3281293
207069_s_at	1.2266808
202237_at	1.0333672
224901_at	1.0448065
201510_at	1.0041595
218541_s_at	1.6625738
219529_at	1.1021357
227654_at	1.4767504
227450_at	1.1039557
219427_at	1.0745959
202688_at	1.3788414
227145_at	1.3328528
226333_at	-1.2042165
202687_s_at	1.4472313
232184_at	-1.0183463
226636_at	1.0068555
227811_at	1.0645761
204435_at	-1.0245776

204933_s_at	1.6477222
239370_at	1.0660491
214329_x_at	1.4095469
205681_at	-1.0095673
229842_at	1.2734013
204932_at	1.2887297
236656_s_at	1.032403
230999_at	1.2828364
223599_at	1.0877509
238983_at	1.264193
216598_s_at	1.4123087
201565_s_at	1.1097231
1555852_at	1.2201576
201465_s_at	-1.2440495
239241_at	1.2980499
201566_x_at	1.1286325
236982_at	1.4327459
223595_at	1.0227633
228890_at	1.2756281
221729_at	1.3601937
229817_at	1.3145056
206632_s_at	1.0809174
201744_s_at	1.4999299
210912_x_at	1.0666637
209716_at	1.1780539
204298_s_at	1.1342854
210166_at	1.1930337
1552365_at	1.0242271
207761_s_at	2.1392288
228067_at	1.1063595
239169_at	1.0079465
227925_at	1.7581882
239085_at	1.1485205
207339_s_at	2.1270704
201009_s_at	-1.712697
212614_at	1.32863
203278_s_at	1.0500307
201008_s_at	-1.7617574
225987_at	1.7344642
221766_s_at	1.0192184
222857_s_at	1.1899462
204288_s_at	1.4374065
205870_at	1.0470085
214321_at	1.5143228
203153_at	1.7884102
219125_s_at	0.56718826

210346_s_at	0.62651443
242864_at	0.47727394
229069_at	0.0400362
209636_at	0.3502736
242020_s_at	0.7259226
207219_at	0.05929184
242998_at	0.3562193
1559535_s_at	1.0483294
227222_at	0.4251933
49452_at	0.4742403
209281_s_at	0.18825626
208813_at	0.29461384
236442_at	0.13952589
241808_at	0.4179368
221667_s_at	0.34336233
230036_at	0.9095063
1554148_a_at	-0.007556915
230329_s_at	0.71143246
218627_at	0.36462688
209200_at	0.25388765
225728_at	1.6375589
229231_at	0.25697517
235522_at	0.4223323
219959_at	0.25593376
204605_at	0.17687798
229759_s_at	0.37577105
233647_s_at	-0.051506996
218976_at	0.4638908
227572_at	0.81559896
229700_at	0.09179163
226546_at	0.5421319
205652_s_at	0.8200536
218692_at	0.18822002
200670_at	0.37972832
64900_at	0.46251488
210993_s_at	0.25763464
233564_s_at	0.04490614
235608_at	0.016537666
212971_at	0.73509216
215716_s_at	0.1819992
1569864_at	-0.36268067
222316_at	0.15296745
202558_s_at	0.043524742
209205_s_at	0.73108006
237127_at	0
1569973_at	0.3304987

202557_at	0.29939365
1553301_a_at	-0.028720379
228354_at	0.452343
226084_at	0.25249052
229255_x_at	-0.21165371
1553987_at	0.24513721
AFFX-HUMRGE/I	0.39160728
213452_at	0.35842228
204340_at	1.1092014
227230_s_at	0.69823885
220466_at	0.30490112
227859_at	0.17388058
224792_at	0.7173662
203508_at	0.5603018
239012_at	1.0980706
213393_at	-0.28685427
219736_at	0.023245335
219290_x_at	0.30974197
242313_at	1.3210874
228316_at	0.40967083
222585_x_at	0.4885831
219287_at	1.1413441
1554249_a_at	0.11178589
204203_at	0.15233517
225626_at	0.008088112
228812_at	0.41024733
228768_at	0.37744713
230185_at	0.10480833
201010_s_at	-1.5112629
235369_at	0.62027884
212634_at	0.1783104
231416_at	0.34339762
240983_s_at	0.67780113
204286_s_at	-0.06949234
225420_at	-0.21514463
241825_at	0.27453184
210946_at	0.30846786
225283_at	-1.0138426
1560156_at	0.6275463
235242_at	0.4801569
228291_s_at	0.53800297
225487_at	0.23323822
238383_at	0.04081583
225183_at	-0.059505463
235698_at	0.71399784
235300_x_at	0.102996826

1552664_at	0.2713828
213930_at	0.29397678
1559003_a_at	0.27344894
220121_at	-0.27350998
230383_x_at	1.2972441
209325_s_at	-0.09141445
224835_at	0.21508312
202270_at	0.4813404
228582_x_at	0.44433117
238532_at	-0.10054827
231899_at	-0.12362385
212745_s_at	0.44328928
227052_at	0.955657
230821_at	0.6105161
49306_at	0.7949357
204085_s_at	0.42291594
224452_s_at	0.25901604
203763_at	0.6293349
202509_s_at	0.43418407
237690_at	0.5041051
229963_at	0.20509386
212633_at	0.42175388
233375_at	0.35998583
202772_at	0.6924095
227180_at	0.09539795
225163_at	0.5721979
203765_at	0.41405964
209147_s_at	0.15642548
211555_s_at	0.7976546
238867_at	0.07177544
209750_at	0.46292114
226603_at	1.1985407
202393_s_at	-0.14845562
241745_at	0.38181567
235939_at	0.30904102
243754_at	0.62802553
222787_s_at	1.044344
218651_s_at	1.0268574
219658_at	0.07808304
219020_at	0.6576934
222383_s_at	0.11134267
236623_at	0.91341734
228250_at	0.31368113
220091_at	0.20575905
207381_at	0.42180157
222869_s_at	0.28751373

1553167_a_at	0.54095745
203868_s_at	1.6912284
207001_x_at	0.3028965
214474_at	0.29534912
206118_at	0.48304224
228027_at	0.5016742
235378_at	0.29413795
227268_at	0.16349792
218769_s_at	0.65390396
218953_s_at	0.618351
225919_s_at	-0.139462
229886_at	0.6664505
226534_at	0.48298025
232014_at	-0.11521578
230815_at	0.3223977
225188_at	0.11299038
1568954_s_at	-0.3179679
215209_at	0.11561942
203817_at	1.0014691
1564475_s_at	0.7584801
209795_at	2.0303917
205111_s_at	0.92557955
202887_s_at	1.0419874
229228_at	-2.706733
241617_x_at	-0.1400981
204279_at	0.7283764
210472_at	0.07415581
206101_at	0.23906732
227582_at	0.7866936
225068_at	0.49865055
1558027_s_at	-0.08790636
220183_s_at	0.9163718
213206_at	-0.1539998
238510_at	0.21056366
201502_s_at	0.8149643
229254_at	0.7242775
218912_at	0.22656918
204103_at	0.56576633
1559532_at	0.9878354
213480_at	0.32498264
206448_at	-0.15100431
236321_at	0.47572136
229312_s_at	0.6628337
237154_at	0.6996584
228604_at	0.2896762
218656_s_at	0.49402

202402_s_at	0.78883266
206433_s_at	0.2553587
238912_x_at	0.083413124
207181_s_at	0.0871191
226449_at	0.24250984
1569969_a_at	0.51534605
227125_at	1.0018067
204674_at	0.6879988
230758_at	0.5497298
242727_at	-0.22055912
239451_at	0.29268694
1553815_a_at	0.5128541
218377_s_at	0.6328554
235643_at	1.2511673
227866_at	0.20584583
214453_s_at	0.78077793
238982_at	-0.30601358
228697_at	0.9307289
239142_at	0.34339666
64432_at	0.1599102
244749_at	0.9769845
238853_at	0.53340816
210056_at	0.4942026
228373_at	-0.12885427
229849_at	0.42693472
235199_at	-0.21711254
220673_s_at	-0.043687344
205205_at	0.54585457
222614_at	0.7565565
232167_at	0.3442917
221868_at	0.32414436
228968_at	0.46802092
205112_at	1.1730795
218303_x_at	0.66306114
235611_at	0.18039608
204971_at	0.4551754
212070_at	0.69269276
228867_at	0.2366314
238126_at	0.2763157
219806_s_at	-0.077690125
243661_at	0.32202244
205931_s_at	-1.5606015
208786_s_at	-0.17405319
228568_at	0.46202564
1564757_a_at	0.7293148
1553193_at	0.38103962

240994_at	0.8028774
228969_at	-0.020208836
229596_at	0.18345809
214472_at	-0.41587067
1558094_s_at	0.20316076
205595_at	0.54761696
237654_at	0.8285656
235191_at	0.25174856
202014_at	-0.6807866
222830_at	0.22999
227755_at	-0.019795418
1554287_at	0.19574642
211470_s_at	0.51788807
239682_at	1.077992
228937_at	0.006775379
1557300_s_at	-0.04070711
212423_at	0.21934414
225189_s_at	0.16681576
219647_at	0
202269_x_at	0.5249939
243463_s_at	0.33339596
208791_at	-0.11292696
206858_s_at	0.42868328
226423_at	1.323708
213212_x_at	0.32492304
234929_s_at	0.70113754
1557116_at	1.0698037
214023_x_at	0.87970304
201169_s_at	0.58145714
222444_at	0.7276225
224797_at	0.46144295
223544_at	0.35249186
219211_at	0.29823542
223494_at	-0.3633256
212930_at	0.36004925
244734_at	-0.15799046
213272_s_at	0.6175966
225573_at	0.21029139
227443_at	0.13985348
208158_s_at	0.68969345
225698_at	0.50364685
204786_s_at	0.9778285
208249_s_at	-0.27229214
212239_at	0.14132214
228440_at	0.16777658
218361_at	0.6572981

228441_s_at	0.3519349
228618_at	-0.12825966
1559496_at	-0.089711666
227210_at	0.41363287
207170_s_at	0.64477634
227798_at	0.51206493
242835_s_at	1.1212387
238526_at	0.36961985
232369_at	1.1596198
228328_at	0.0828433
221477_s_at	0.44702053
241418_at	0.02110672
203640_at	1.1319704
235971_at	1.0196805
225539_at	0.2886963
229072_at	0.2386899
208869_s_at	1.3551455
220755_s_at	0.40751076
219551_at	0.11859608
219405_at	0.33096504
209199_s_at	0.54531336
1554447_at	0.69120455
225527_at	0.3204069
224990_at	0.9934597
208965_s_at	0.423378
226474_at	0.45517206
211692_s_at	0.49691534
226962_at	0.69005203
217858_s_at	0.47197437
226135_at	0.77218056
1553683_s_at	1.0030856
229450_at	1.5702238
238078_at	0.381732
209304_x_at	0.5007715
228416_at	0.47241116
215485_s_at	0.48681545
215945_s_at	1.0125546
239007_at	-0.018882513
231950_at	0.33281517
238575_at	0.4269657
201000_at	0.88005066
203165_s_at	-0.006093979
236987_at	0.4801402
220066_at	0.43003988
231577_s_at	0.6354847
243542_at	0.057609558

211458_s_at	1.2050085
209324_s_at	-0.3851509
242586_at	-0.41280556
224989_at	1.2008224
229488_at	0.20391655
207113_s_at	0.95202255
207574_s_at	0.5575199
1553293_at	0.10890627
205890_s_at	1.8413696
205298_s_at	0.6286483
203574_at	0.578187
235143_at	0.001992226
228275_at	0.20280075
204747_at	1.7031059
212686_at	0.9122901
224610_at	-0.06903362
225278_at	0.16741562
239240_at	0.75065184
203556_at	0.6333976
242234_at	0.56971025
223504_at	0.44143295
224492_s_at	1.1124091
232024_at	1.338294
235533_at	0.2527647
228751_at	0.6777768
209425_at	0.9133806
227558_at	0.56949806
224973_at	1.4156318
227809_at	0.47785044
206133_at	0.72662735
37028_at	-0.44534588
213293_s_at	0.7366581
243747_at	0.24193668
207528_s_at	0.41363287
211786_at	0.28985214
208966_x_at	0.58457375
241360_at	0.6167588
202637_s_at	0.68617153
209424_s_at	0.8675904
226757_at	1.7174997
219209_at	0.5908494
226117_at	0.62022686
223692_at	0.5978923
208792_s_at	-0.26990128
228001_at	0.45221138
223471_at	0.45405197

225681_at	0.8079088
215948_x_at	0.28057194
204655_at	0
218532_s_at	0.42813778
214079_at	-0.95312643
204472_at	0.2534442
233329_s_at	0.93368244
223401_at	0.29975748
235142_at	0.16480446
236305_at	0.27855635
209071_s_at	0.3862524
206332_s_at	0.6211319
242916_at	0
236646_at	1.6494107
225220_at	0.3563242
218696_at	0.22093296
209305_s_at	0.57073593
215985_at	0.23923588
209485_s_at	0.6788883
226436_at	1.145494
205773_at	0.16703463
206744_s_at	0.4334221
228661_s_at	0.70451546
222858_s_at	0.22614765
227176_at	0.53031063
228198_s_at	0.43418026
221988_at	0.25032234
239587_at	1.2079587
224763_at	0.8220997
223805_at	0.2038908
213195_at	0.71128464
209545_s_at	0.406106
223821_s_at	0.39852238
212907_at	-0.26163673
222376_at	1.2688594
238790_at	0.7625823
202376_at	0.29111624
202341_s_at	1.177494
220322_at	0
206652_at	-0.028820038
207826_s_at	1.3777828
209355_s_at	0.81446075
234759_at	0
243056_at	0.9159603
207177_at	0.25678325
213820_s_at	0.7171712

228304_at	0.99552965
210001_s_at	0.50558424
209544_at	0.28887272
219195_at	0
218546_at	0.77540684
202375_at	0.15045738
201170_s_at	0.9683161
227004_at	0.24951744
239258_at	0.30792952
231412_at	0.5648341
212226_s_at	0.8725195
235428_at	0.6641569
1556095_at	0.88285255
1557321_a_at	0.29632306
215009_s_at	-0.026354313
219684_at	1.8950377
226337_at	0.13554764
241455_at	0.98017216
1556096_s_at	0.6815944
205114_s_at	-0.51185465
206271_at	0.8630085
223773_s_at	-0.01599884
213704_at	0.13684845
205943_at	0.23515677
222859_s_at	0.37995148
225344_at	1.9254332
230387_at	0.47963333
203797_at	1.0842905
212240_s_at	0.29424143
230142_s_at	0.6471138
205698_s_at	1.6174369
220606_s_at	0.4078083
236798_at	0.42444897
242329_at	-0.4334402
220672_at	0.39777565
229526_at	0.6454382
242907_at	1.1754217
228184_at	0.31340504
217966_s_at	0.64439774
1557236_at	1.0347948
224826_at	0.43836308
219583_s_at	0.99736214
205342_s_at	0.31135607
228181_at	-0.5542784
201294_s_at	0.6271801
244377_at	0.8573947

244070_at	0.34341192
235620_x_at	0.3644209
220177_s_at	2.1313615
224964_s_at	1.1740441
202531_at	0.86906433
212230_at	0.9546604
219910_at	0.2549038
35974_at	1.4968529
235342_at	0.40645027
202342_s_at	1.2350998
202023_at	2.128282
1554418_s_at	0.80940056
238567_at	0.76594067
213056_at	1.3115377
228617_at	0.96749735
240572_s_at	0.91053915
238681_at	1.0741405
215071_s_at	0.19470692
227354_at	-0.21489382
227803_at	1.0952435
220302_at	0.57874966
228153_at	1.9087796
217967_s_at	0.77984905
218145_at	0.9499254
233002_at	0.53718996
235583_at	0.8056874
206157_at	0.7312603
210587_at	0.630043
219477_s_at	0.124678135
1553211_at	0.50858665
219316_s_at	0.85934734
230560_at	1.0846701
225540_at	0.14007092
217371_s_at	0.85459995
241869_at	1.1764145
238725_at	0.98800087
202638_s_at	0.77699375
1555788_a_at	0.7390485
242649_x_at	1.7126384
210163_at	0.70557976
201397_at	1.280324
219716_at	0.863472
205352_at	0.2878914
214954_at	0.48485708
1554980_a_at	0.24815226
238430_x_at	1.5274525

223611_s_at	1.0916033
223774_at	0.047254562
226560_at	1.0869436
1555759_a_at	0.11166763
238029_s_at	1.0554719
202510_s_at	1.3510056
226267_at	1.7545366
231956_at	1.7150345
1405_i_at	0.076408386
238228_at	0.55965424
213555_at	0.91076565
225496_s_at	0.6988416
1554743_x_at	0.40410852
232914_s_at	0.6779647
205027_s_at	1.7020555
227982_at	0.66191626
200924_s_at	0.52315426
226725_at	1.6841512
1554741_s_at	0.26420355
205194_at	1.4910097
228427_at	1.027977
242397_at	1.3452358
1554462_a_at	0.28372097
226899_at	0.69891167
213900_at	0.32742262
205569_at	0.34691286
233500_x_at	1.0767865
223405_at	0.13066149
227481_at	0.16370344
1554742_at	0.4957776
202842_s_at	0.45522118
202847_at	1.2545252
215641_at	0.20111895
211600_at	-0.22956753
214012_at	1.0045738
221577_x_at	0.47222328
244780_at	1.2343512
232000_at	0.8360784
230405_at	1.2133145
203304_at	0.9482775
210004_at	1.5966835
214823_at	1.9477558
205992_s_at	1.3596816
1558212_at	1.1320043
205048_s_at	1.5223346
226181_at	0.7293911

235075_at	1.5631528
223195_s_at	0.87304735
1557078_at	1.3010097
217168_s_at	0.9794121
205830_at	0.30069375
223196_s_at	1.178709
217546_at	1.5832186
1553055_a_at	1.4624372
221210_s_at	-0.2282629
209921_at	1.0617466
203725_at	0.85179806
207536_s_at	0.48768187
208763_s_at	1.2428341
217678_at	0.96764755
243999_at	1.6162004
219338_s_at	0.42944622
202843_at	0.2903595
205590_at	1.8506632
211122_s_at	0.9653599
202672_s_at	0.056657314
219270_at	1.6602807
209383_at	1.2409248
205047_s_at	1.397788
208370_s_at	1.9797192
232593_at	1.604547
206085_s_at	1.3045058
220132_s_at	1.4714601
217127_at	1.7673073
215253_s_at	1.9210496
231202_at	2.8864741

Regorafenib high dose v.s. solvent (fold-change)	Gene Symbol
-4.7653284	IL11
-3.3417702	SERPINB2
-3.0477438	DUSP4
-2.8915658	DUSP6
-2.8615417	DUSP6
-2.834301	DUSP6
-2.8153882	DUSP4
-2.7409768	GPR110
-2.6956615	TNS4
-2.6867933	PRPF4B
-2.6087976	FUT8-AS1
-2.526566	SCG5
-2.4919577	HMGA2
-2.4720068	AKAP12
-2.4532862	LDLR
-2.449305	SHISA2
-2.4445105	RNASE7
-2.4190488	DUSP4
-2.4044409	ADRB2
-2.3993077	19B1///MIR20A///MIR92A1
-2.3950586	FOXL1
-2.380568	ADAMTS6
-2.347309	GPRC5A
-2.272852	NR2F2
-2.1994228	KRTAP2-3///KRTAP2-4
-2.1977005	
-2.1965852	MIR210HG
-2.185413	
-2.1669202	FST
-2.152841	ARL14
-2.1492443	AKAP12
-2.1466799	ANKRD1
-2.1117892	S100A6
-2.1101875	GPRC5A
-2.1088433	GPR110
-2.104947	EMP1
-2.098024	VNN1
-2.0378342	IL1B
-2.0251317	PLAT
-2.017828	NOG
-2.016217	
-2.0160995	TGFB2
-2.0048218	NT5E
-2.0016117	LDLR
-1.9885588	THBS1

-1.9793634	MAT2A
-1.9460039	HPDL
-1.9345388	MYADM
-1.9246392	SLC6A14
-1.9230018	C1orf116
-1.9227057	FOXQ1
-1.9159975	00175704///RP4-555D20.2
-1.9130507	IL1B
-1.9074135	DKK1
-1.9048157	LOC100127972
-1.8799348	LOC100130476
-1.8781214	
-1.8674421	MIR210HG
-1.8649292	THBS1
-1.8525071	MN1
-1.8497643	MMP10
-1.8472385	CTGF
-1.8466024	PI3
-1.8330269	GJC1
-1.8324242	TGFB2
-1.8287292	ANTXR2
-1.8163452	
-1.8129978	FST
-1.8092737	GJB3
-1.8079028	PI3
-1.8011193	PTPRB
-1.7948427	CD59
-1.7941799	AKAP12
-1.7896442	000015137///RP11-30P6.6
-1.7816677	NR2F2
-1.7777734	
-1.7725973	LOC100506377
-1.7673469	ADAM19
-1.7536674	ITPRIPL2
-1.7516847	
-1.7461367	HOXB-AS3
-1.7450356	DUSP2
-1.7432036	WNT3
-1.7427449	DIDO1
-1.7406306	CD59
-1.7393379	
-1.7265725	TGOLN2
-1.7167645	
-1.7124996	RASAL2
-1.7106819	BMP4
-1.7037067	

-1.7007985	TOX3
-1.6991439	00172465///RP11-265N7.1
-1.6986761	CDH6
-1.6984262	ITPRIPL2
-1.6948929	
-1.6890955	HK2
-1.6890125	MAT2A
-1.6816251	LOC401068
-1.6815071	SLC2A1
-1.679862	MMP1
-1.6782074	AIM1L
-1.6705456	FST
-1.6677752	ANTXR2
-1.6639466	GPR110
-1.6549473	CD59
-1.6501522	
-1.6490722	BICD1
-1.641078	IGFBP1
-1.639328	GPR161
-1.6388316	MYL12A
-1.6336594	MYC
-1.6215429	MALT1
-1.6204205	PHLDA1
-1.6101007	SLC14A1
-1.6091356	PIGM
-1.6059184	NABP1
-1.6049705	PPP1R10
-1.5970826	ARHGAP29
-1.5870042	GJB3
-1.5868516	PNMA2
-1.5852323	EVI2A
-1.5834231	THBD
-1.5801992	ITPRIPL2
-1.5801258	MATR3///SNHG4
-1.5769472	FJX1
-1.5704699	PLXNA1
-1.5694594	LOC100505738
-1.5660462	LDLR
-1.5657682	LAMA3
-1.5638456	LOC729680
-1.5627651	DGAT2
-1.5591664	IRS1
-1.5559454	0000184015///RP6-99M1.2
-1.552414	MAFF
-1.5455217	RNASE7
-1.5375652	CD59

-1.5368686	
-1.536397	CD3EAP
-1.5314136	VNN1
-1.529594	ZBTB47
-1.5272865	KIAA1199
-1.5271144	4-Mar
-1.5177608	CARD10
-1.5176101	HEG1
-1.5168686	HCG4
-1.5148878	ESRP2
-1.5140376	SPRY2
-1.5117512	TMEM165
-1.5069385	'//OTTHUMG00000172354
-1.5048513	INHBA
-1.5013943	FOSL1
-1.4964657	ITPRIPL2
-1.495892	HK2
-1.4957743	TMEM165
-1.4949307	SFN
-1.4944277	HEXIM1
-1.4902525	SMURF2
-1.4882898	CTSC
-1.4866204	FGFBP1
-1.4833264	HHIP
-1.4817524	
-1.4816709	LIPG
-1.4814777	C1orf116
-1.4797702	HOXB5
-1.4776249	AQP3
-1.4764423	MIR21///VMP1
-1.475749	SEMA7A
-1.4731979	
-1.463428	SYNCRIP
-1.4614267	INHBA
-1.4606357	
-1.4602757	COL8A1
-1.4584794	AGTR1
-1.4539003	PIGM
-1.4499254	NT5E
-1.4471638	SLC14A1
-1.442173	SERPINE1
-1.441247	F3
-1.44034	LOC284454
-1.4351578	AMIGO2
-1.4349861	PURB
-1.4310179	HS3ST1

-1.4287815	PTPRE
-1.4256692	
-1.4226894	THBD
-1.4193068	TGOLN2
-1.4186459	ZADH2
-1.4183383	AGTR1
-1.4157138	FUT11
-1.4093533	TM4SF18
-1.4087095	TNFRSF10A
-1.40798	ZBED2
-1.4067898	RBM15B
-1.406435	PIGM
-1.4038382	NUP98
-1.4037733	ZC3HAV1L
-1.4014635	THBD
-1.3988934	GJA1
-1.3947964	TM4SF1
-1.3918638	SFN
-1.3904347	TBC1D30
-1.3887072	TARDBP
-1.3842564	CLCF1
-1.3767104	EXOSC6
-1.3759685	PCGF3
-1.3757977	
-1.3756347	CMTM3
-1.3739529	CTSC
-1.371891	DIS3L2
-1.371871	TMEM185B
-1.3640475	LPAR1
-1.3611512	NT5E
-1.359786	HS3ST1
-1.3597703	HTR7P1
-1.358151	
-1.3574905	RASA1
-1.3571701	ATF5
-1.3557186	FAM49B
-1.3554182	RGMB
-1.3501725	4///ZDHHC11///ZDHHC11B
-1.34757	LTBP2
-1.345459	C1orf116
-1.3433495	LOC643837
-1.340406	ZNF143
-1.3403649	RNASE7
-1.3386145	PSMA5
-1.3310871	UBASH3B
-1.3286924	TMEM19

-1.3282938	MIR612///NEAT1
-1.3280578	LRRC8A
-1.3278089	
-1.3274527	CBX3
-1.3267679	UBALD2
-1.3261285	SNORD50A///SNORD50B
-1.3231359	ENY2
-1.3224287	MALAT1
-1.3210263	RPL27A///SNORA3
-1.320528	SFN
-1.3182914	TRPC4
-1.3152554	C15orf54
-1.3144107	FOXL2
-1.3130827	DUSP5
-1.3101583	
-1.309617	SLC2A1
-1.3057284	TGOLN2
-1.303474	SH3TC2
-1.3012137	CD274
-1.299407	LPAR1
-1.2967129	LOC100996496///SFPQ
-1.2941437	LCMT2
-1.2920308	GPRC5B
-1.2914939	HOXB-AS3
-1.2914476	TNFRSF10A
-1.2907696	LOC201651
-1.2899704	TSPAN14
-1.2868056	MALT1
-1.2840009	POU2F2
-1.2830167	GALNT5
-1.2793236	ITGA2
-1.27883	HOXB6
-1.2786274	TM4SF1
-1.2781649	RBM12
-1.2762728	MAFF
-1.2752409	CXCR4
-1.2750173	ANTXR2
-1.2719111	PTPRB
-1.2685857	ZBTB42
-1.2672424	WNT7A
-1.2670965	EMP1
-1.2585058	PHF23
-1.2582941	CCDC71L
-1.2560725	C5orf24
-1.2531805	S100A10
-1.2524967	NANOS1

-1.2523546	MTAP
-1.2516823	PHF23
-1.251606	PHLDA1
-1.2512608	LOC152225
-1.2492824	ZADH2
-1.248323	PPP1R10
-1.2481327	IRS1
-1.2466087	FLVCR1
-1.2442307	RIMKLB
-1.2431602	SRSF1
-1.2414415	
-1.2393608	MACF1
-1.2365055	KLF2
-1.2356138	GPR126
-1.234509	HOXB5
-1.234509	
-1.2341871	C10orf2
-1.2332773	MACF1
-1.2320485	SRSF11
-1.2305288	PURB
-1.2281885	TMEM201
-1.2275333	FAM83H
-1.2267075	TMEM19
-1.2262344	HEXIM1
-1.2260761	B3GALT6
-1.2226038	HNRNPU
-1.2216787	XPNPEP3
-1.2211275	SLC16A3
-1.2211146	B3GNT7
-1.2207909	GPR125
-1.2206917	
-1.2192559	
-1.2171159	HHIP
-1.2167063	TM4SF1
-1.2162819	MACF1
-1.2140746	SH3KBP1
-1.2107759	ILF3-AS1
-1.2098389	MALT1
-1.20895	ITGA2
-1.2084522	178927///RP11-196G18.23
-1.2063694	MCM3AP-AS1
-1.2051411	'//OTTHUMG00000086919
-1.204431	RBM12
-1.2040882	LINC00667
-1.2039337	ETS1
-1.202899	FLJ37453

-1.2028351	CXCR4
-1.1930017	PDLIM4
-1.1923995	ARHGEF39
-1.1905127	EPM2AIP1
-1.1902914	TMEM92
-1.1902509	THAP10
-1.1891136	MYADM
-1.1885977	LOC158402
-1.1883898	SLC7A6
-1.1866751	SLC16A3
-1.1863918	TUG1
-1.183073	EMP1
-1.1830664	METAP2
-1.181694	GEMIN4
-1.1797099	OTUB2
-1.1792345	LOC101060521///POLR3E
-1.1784463	PATZ1
-1.1774921	VPS53
-1.1773429	SLC7A6
-1.1756535	PDLIM5
-1.1743212	METAP2
-1.1730008	'//OTTHUMG00000172744
-1.1718388	CYP1B1
-1.171566	PDLIM5
-1.1689844	STC1
-1.1667037	
-1.1656771	ANKRD37
-1.1651464	GFOD1
-1.1650105	MIR636///SRSF2
-1.1647582	ANXA10
-1.163259	ZADH2
-1.1630383	UHRF1
-1.1628232	FLJ38717
-1.1622787	NR2F2
-1.1614528	\63///SNORA81///SNORD2
-1.1610084	LINC00525
-1.1599874	CXCR4
-1.1597137	KRT6A
-1.1562614	MTAP
-1.1559882	IL37
-1.1549549	C16orf52///LOC101060634
-1.1507087	VMP1
-1.1499987	TRIB1
-1.1499863	'9///SNORD80///SNORD81
-1.1496639	ARL4C
-1.1496143	MDN1

-1.1479616	LAMC2
-1.1479087	LEPROT
-1.1477685	SRSF7
-1.1464486	00032910///RP11-157P1.4
-1.1456971	PROSER2
-1.1453505	ARGLU1
-1.1439009	C4orf3
-1.1409369	EMP1
-1.1404357	
-1.1401958	INHBB
-1.1395669	TBRG4
-1.1373882	GPRC5A
-1.1367817	STC1
-1.1351962	TMPO-AS1
-1.1350679	ACOXL
-1.1329503	UBE2Q1
-1.1327796	
-1.1327429	
-1.1323152	
-1.1322904	LOC100996341
-1.1322279	UBASH3B
-1.132122	TRHDE
-1.1319876	LPAR1
-1.13167	EPHB6
-1.1311803	COL8A1
-1.1311312	TGOLN2
-1.1297693	CD274
-1.1277022	CDC42EP2
-1.1273327	CHML
-1.1242595	SLC7A6
-1.1236515	NSD1
-1.1220737	ERF
-1.1214032	C5orf24
-1.1213999	TPM3
-1.1213636	RASA3
-1.1206226	LOC285628///MIR146A
-1.1202874	BDNF
-1.1193085	TGFB2
-1.1182246	C17orf96
-1.11709	TMX3
-1.1165004	BAIAP2-AS1
-1.1155958	SLC19A1
-1.1155939	RRS1
-1.1155472	CEACAM6
-1.1138315	TOR4A
-1.1097198	

-1.1086721	DGCR14///TSSK2
-1.1080546	ZNF514
-1.1078262	TCN1
-1.1077728	LINC00938
-1.1077132	ERVMER34-1
-1.1072664	CHPF2
-1.1064	
-1.1063452	IRS1
-1.1050804	'//OTTHUMG00000172354
-1.103034	FLVCR1
-1.1030097	RGCC
-1.1028209	CDS2
-1.1024227	HHIP-AS1
-1.101675	PDLIM5
-1.1009812	DDX46
-1.099854	LRFN4
-1.0977669	NLK
-1.097023	QKI
-1.096931	NR3C1
-1.0967607	CBX3
-1.0963593	RBM4
-1.0948696	CALM1///CALM2///CALM3
-1.0943856	EVI2B
-1.0903234	MISP
-1.0898523	SOX9
-1.0888538	SLC4A7
-1.0888319	SOX9
-1.0879426	DIO2
-1.0868397	LETM1
-1.0860982	TOX3
-1.0857449	MTAP
-1.0846167	ZC3H4
-1.0840383	POU2F2
-1.0837097	CCL28
-1.0808382	RCL1
-1.0800867	PROSER2
-1.0792408	POU2F2
-1.0782199	POU2F2
-1.0770302	AKAP1
-1.0755529	
-1.0751705	
-1.0744643	SMURF2
-1.0718927	ARL4C
-1.0715218	NR2F2
-1.071209	MMD
-1.0711212	DAPK1

-1.0703363	ATXN7L3B
-1.0689783	0190986///LOC101060564
-1.0684233	BCLAF1
-1.0677996	MTAP
-1.0665679	PRPF4B
-1.0656471	
-1.0655475	TNFRSF10A
-1.065445	ARL4C
-1.0650749	SIM2
-1.0641737	F2RL1
-1.0641336	ELK4
-1.063467	
-1.0614657	ZADH2
-1.0611653	ASXL1
-1.0609288	C9orf152
-1.0606985	ZFP36L1
-1.0597715	LRRC8A
-1.0584431	TOR4A
-1.0578618	DLG1
-1.0576401	HOXB3
-1.0569811	RASA1
-1.0561786	
-1.053649	GPR110
-1.0525551	GLIPR1
-1.050767	THBS1
-1.049962	C15orf52
-1.0498486	ARHGAP23
-1.0495672	MRPS12
-1.0494037	CYP1B1
-1.0484381	CALM1///CALM2///CALM3
-1.0476985	FAM117B
-1.047555	TMEM158
-1.0470295	0159296///RP11-271C24.3
-1.0459809	GPR161
-1.0446548	MARCKS
-1.0446286	ING5
-1.0432968	UBASH3B
-1.0431385	MAFK
-1.0426121	TMEM19
-1.0424023	ZMYND19
-1.0419745	CHPF2
-1.040204	
-1.0400429	ALDH1A3
-1.0398636	LRP8
-1.0369368	EIF4E2
-1.0351791	AMD1

-1.0350962	ETS1
-1.0341206	CCND1
-1.0339723	ATP10A
-1.0322604	RGS4
-1.0312438	DGKH
-1.0300403	TNFSF14
-1.0299168	URB2
-1.0284662	NSD1
-1.0264521	AK4///LOC100507855
-1.024662	IMP3
-1.0240793	GEMIN6
-1.0229826	
-1.0225644	TGFA
-1.0222406	TNFSF11
-1.020601	TOX3
-1.0199513	PXDN
-1.0198045	C8orf58
-1.0195723	POGK
-1.019022	CHML
-1.0180387	DAW1
-1.0179548	BCL2L1
-1.0173483	LOC100996496///SFPQ
-1.0167465	MACF1
-1.0144858	CEACAM6
-1.0143337	RNF44
-1.013843	TNS4
-1.0137262	EHF
-1.0135555	CTSC
-1.0128412	LOC100505729///VPS8
-1.012732	IL11
-1.0124364	
-1.0116792	UBALD2
-1.0114398	EHF
-1.0103703	NOP16
-1.0102186	HBEGF
-1.0085535	DHX9
-1.0081587	C11orf95
-1.0073957	ZFAS1
-1.0066147	
-1.0065951	PRDM10
-1.0059671	VCL
-1.005569	INHBA
-1.0044689	NR2F2
-1.0044374	TM4SF1
-1.0043387	PDLIM5
-1.0039053	LFNG

-1.0037975	FAM60A
-1.0034657	
-1.002708	'//OTTHUMG00000172354
-1.0012755	EED
-0.99062014	HAS3
-0.9758644	HBEGF
-0.96754646	LOC100505812
-0.96659136	LINC00883
-0.95915556	S100A8
-0.95485353	DLEU2
-0.95334196	API5
-0.9434047	UTP15
-0.9312415	LETM2
-0.92885923	DUSP1
-0.9221573	TAF4B
-0.9105277	MCL1
-0.90669537	SRSF6
-0.9029908	2///LOC728802///PDE4DIP
-0.89949226	AGPAT9
-0.887074	PHLDA1
-0.88335085	00179824///RP11-173M1.8
-0.8758879	LRRC8C
-0.86548805	PTHLH
-0.8621597	FGD6
-0.85362816	TFB2M
-0.8295274	NAV3
-0.79129124	CTPS1
-0.7874365	
-0.78020287	EGR1
-0.7643895	NOC2L
-0.7623229	UBIAD1
-0.7438345	
-0.73940563	DNMBP
-0.7382741	
-0.7360978	
-0.723917	MCL1
-0.6939564	MCL1
-0.6659751	NDUFAF4
-0.643137	LYAR
-0.6354089	PNO1
-0.62041855	NAV3
-0.61398935	SGMS2
-0.60382366	CYCS
-0.60078335	TNFRSF10D
-0.5786905	SLC25A32
-0.566628	

-0.56317425	GRPEL1
-0.548584	ARHGAP29
-0.5447235	KLHL21
-0.54122496	TXLNG
-0.5389452	EGR1
-0.5388627	MIR100HG
-0.521646	RCSD1
-0.4980569	MTMR1
-0.4621277	YRDC
-0.45597076	LBH
-0.4482355	
-0.40985346	ING3
-0.36180878	TSC22D1
-0.29241943	PHLDA2
-0.26754665	TSPAN8
-0.26332474	CCDC82
-0.13866329	KBTBD8
-0.12958002	PCDH7
-0.10509682	JUN
-0.021551132	BLACAT1
0	JUN
0.055818558	VTCN1
0.07112503	FAM198B
0.07131386	IGFL1
0.07289171	CLIC5
0.10606289	MYLK
0.11457634	WNT10A
0.123440266	SOSTDC1
0.13314533	COL4A3
0.14795637	SMAD6
0.17808533	NNMT
0.18086243	SCD5
0.1845336	ELF3
0.19161797	C8orf4
0.20162487	CLIC3
0.22675467	FAM65C
0.23251486	ERP27
0.2430296	FAT4
0.26915646	TNFSF10
0.28625345	LOXL4
0.28630304	IL6R
0.2963972	TNFSF10
0.31002998	ALS2
0.31589985	PLD1
0.3183365	FGD3
0.33488655	NUPL1

0.33873558	TNFRSF11B
0.3398223	LOC100505633
0.35113716	TNFSF10
0.36127186	BCL2A1
0.36522722	ELF3
0.36768913	TNFRSF11B
0.38346386	LOC100288911
0.3895812	
0.3947425	TRIM6
0.43393898	NSUN7
0.44674683	CCL2
0.46076584	ID2
0.46412754	LOC100507463
0.46430683	JUN
0.4837408	00167327///RP11-315O6.1
0.5167732	ID2
0.53277063	
0.53931236	TMEM133
0.5664334	ATOH8
0.61136293	COL5A2
0.6384382	ZNF608
0.6569271	APOBEC3B
0.70525265	LUM
0.7191067	GSTM4
0.7230177	CSF1
0.72994184	LOX
0.7560501	TLR5
0.7570195	SCIN
0.75943613	METTL7A
0.78410816	KIAA1211L
0.82082796	RDM1
0.8351202	ST3GAL4-AS1
0.84608746	JDP2
0.85711956	LTB
0.8705034	LOC101060503///TXNIP
0.8801093	ARID5B
0.8827877	PHF21A
0.920104	LOC101060503///TXNIP
0.9393859	STEAP4
0.94031715	FAM46A
0.94563293	KCNMB4
0.95922375	SORBS2
0.9799714	BDKRB2
0.98568535	NOV
0.9885359	IFIT1
1.0000048	SLC50A1

1.0008478	CLK4
1.0016775	ZNF554
1.0027089	SARNP
1.0029716	NFKB2
1.0033793	ZBP1
1.0036387	ZFP69B
1.0040126	RDH12
1.0049009	//OTTHUMG00000163262
1.0055718	FBXO10
1.005961	ACACB
1.0064421	ATP2B1
1.006588	GOT1
1.006712	DPF3
1.0071778	ZC2HC1A
1.007895	HSPB8
1.010407	SAMD9L
1.0107155	SLC33A1
1.0116673	NUDT6
1.0125237	DRAM1
1.0131011	MEF2C
1.0134525	SORBS2
1.0144138	LRRC37B
1.0146217	CLEC2D
1.0171833	MOCOS
1.0175982	CGRRF1
1.0180478	VEPH1
1.0185423	CDADC1
1.0193336	DNAJC12
1.0204597	USP30
1.0209017	ZNF738
1.021277	LOC100506844
1.0214858	TTLL1
1.0218544	SYBU
1.0225716	XBP1
1.022943	TMEM231
1.0235591	SMAD1
1.024055	CDADC1
1.0241361	
1.0246716	CARS
1.0261688	ATP2B1
1.026195	SERAC1
1.0267591	
1.0270147	HSPA13
1.0271225	LMO4
1.0288358	
1.0298619	SEPT7P2

1.0300007	HSPA13
1.0306234	TMEM182
1.0306587	MORN4
1.0306897	MAP1B
1.0311975	
1.0328112	MAPKAPK5-AS1
1.0337791	
1.0343785	ZNF184
1.0345373	TMEM187
1.0357356	KIAA1211
1.0358262	CCDC15
1.0373878	DNAJC27
1.0373988	TNKS1BP1
1.0379844	TNFRSF1B
1.0389867	RNF144B
1.0396605	MFSD9
1.039782	TRIM36
1.0405712	DAPP1
1.0416183	LOC728730
1.0417094	CLHC1
1.0420876	KRCC1
1.0451398	KCNMB4
1.0453181	ZNF638///ZNF638-IT1
1.0467167	CEBPG
1.0485492	PAG1
1.048943	
1.0506039	FNIP1
1.0551529	THAP9
1.0578547	LOC101060503///TXNIP
1.0583572	C14orf28
1.0586648	UFL1
1.0595994	DHDH
1.0596476	CARS
1.0600567	PMAIP1
1.0610337	GPAM
1.0616512	TYW5
1.061924	PPAP2A
1.0620041	ARRDC4
1.0620894	0168442///RP11-705C15.3
1.0628023	)0179927///RP11-373L24.1
1.0643787	PLK1S1
1.0647893	TMEM18
1.0654907	C6orf58
1.0660429	C16orf72
1.0660477	ZFP90
1.0660992	RCHY1

1.0666404	FLCN
1.0672002	ATG12
1.0673928	CCDC163P
1.0682201	LINS
1.0691037	SLFN5
1.0706515	RGS16
1.0726848	GPCPD1
1.0735369	GBP1
1.0744667	MALAT1
1.0766349	DPF3
1.078062	ZC3H12C
1.0789356	BBS4
1.0795393	
1.0812969	ZNF148
1.0817604	RASSF4
1.0830159	CLN5
1.0864906	FAM220A
1.088211	DYNC2LI1
1.0894718	TNFAIP2
1.0898309	GPR115
1.0915895	BEX5
1.092823	UFL1
1.0946431	EFCAB2
1.0951719	HMGCL
1.0955124	ELOVL7
1.0958762	FRMD4A
1.0959187	GCA
1.0979891	PPAP2A
1.1019607	GUCY1B3
1.102798	TMEM182
1.1031871	NR1D2
1.1043692	SAMD9L
1.1046267	KLF10
1.1076086	LOC100507557
1.1087604	00175830///RP1-228H13.5
1.1088037	
1.1092491	TMEM106B
1.109416	LARP6
1.1101727	PTCD2
1.1105442	HS1BP3
1.1116211	ALOXE3
1.1132736	ATP1A1OS
1.1138654	FNIP1
1.1151953	SLC2A6
1.1152177	ALOX12B
1.1159868	ELAC1

1.1161752	SEPSECS
1.1161928	VCAM1
1.1164279	TSC22D3
1.1167345	LOC101060511///PRKAB2
1.1187134	STAT4
1.1206303	X5-GPRASP2///GPRASP2
1.1223392	FAM161B
1.1239634	RNFT1
1.1248083	ANKRA2
1.1249952	PCYOX1L
1.1253152	C9orf72
1.1253767	C5orf34
1.1258798	KITLG
1.1262493	ZNF30
1.1265817	LOC389765
1.1279554	RAPH1
1.1282115	C16orf72
1.1282387	SEC24D
1.1288934	GUCY1B3
1.1302333	ZBED3-AS1
1.1306248	CD69
1.1311226	PLCE1
1.131216	DDIT4
1.1314292	CREB5///LOC401317
1.1329951	
1.1332531	PSMB9
1.1337185	MT1G
1.1341636	ECM2
1.1353207	KLHDC9
1.1358213	KLHL12
1.1374993	LOC101060511///PRKAB2
1.1405363	NUDT6
1.1408405	GOSR2
1.1410122	ZNF720
1.1423197	NFKBIA
1.1425357	MFSD4
1.142849	GCC1
1.1431417	CCL4
1.1440306	ARIH2OS
1.1457214	VAMP4
1.1458073	ZNF365
1.1463714	FAM200B
1.14677	GKAP1
1.1500869	000036478///RP1-28O10.1
1.1517758	FAM76A
1.1518278	LHFP

1.1518936	CARS
1.1524291	SPOCK3
1.1555805	C9orf85
1.1568842	CASP7
1.1572866	CEP120
1.1582727	UNC13C
1.1594262	IFNAR2
1.1594472	LRMP
1.1608934	GEMIN8
1.1609073	ARL5B
1.1610923	
1.1615252	TCEANC
1.1626711	RWDD2B
1.1627755	SAMD9L
1.1632919	LOC100505519///TIAM2
1.1637602	IFI44
1.1641226	DENR
1.1665697	HINT3
1.1671658	RFESD
1.1681719	MAPKAPK5-AS1
1.171946	
1.1722426	RAB3IP
1.1748333	RND1
1.1754107	C16orf72
1.1754718	WIPF3
1.1754956	RNF125
1.1790843	PPP4R4
1.1796408	RELB
1.1803207	RWDD2B
1.1810727	SLC2A11
1.1821866	PAIP2B
1.1833901	ZNF449
1.1834793	PLCE1
1.1857481	KRCC1
1.1875348	SREK1
1.1878138	CSTA
1.1880436	GPR56
1.1886106	TATDN3
1.1891527	RSBN1L-AS1
1.1891546	SMCO4
1.1897602	ZNF273
1.1898665	CREB5///LOC401317
1.1907272	MAP1LC3B
1.1913514	GCOM1///MYZAP
1.1916165	CCDC148
1.1940632	ZNF441

1.1946411	
1.1948891	AGR2
1.1958947	AMDHD1
1.1976647	3H///HIST1H3I///HIST1H3J
1.1998138	CCDC174
1.2009811	DSG3
1.2026005	PPP1R36
1.2034025	LINC00662
1.2084007	PPP1R15A
1.20891	GRHL1
1.2089224	00178878///RP11-214C8.5
1.2094059	TRIM4
1.2096701	SULT1C2
1.20998	
1.2101364	LACC1
1.2112665	
1.2119884	ZCCHC24
1.2136917	RAPH1
1.2184358	POPDC2
1.218688	GBP1
1.2205038	RIT1
1.2260866	CLU
1.2273455	HOXC6
1.2273469	PAQR8
1.2281046	//GOLGA6L5///GOLGA6L9
1.2313385	SPATA7
1.2333999	APOL6
1.2347927	TUBB2B
1.2349548	BHLHE40
1.2354054	ARMCX3
1.2375154	ARRDC3
1.2383742	TMEM79
1.2385345	USP18
1.2392898	MGEA5
1.2430124	ATP2B1
1.2443233	MTHFSD
1.2455053	TMEM159
1.2474833	/NPHP3///NPHP3-ACAD11
1.249567	LURAP1L
1.250206	OSBPL1A
1.250287	EPB41L4A-AS1
1.250639	IFNAR2
1.2516518	TGDS
1.251955	PIK3R1
1.2521372	.64P12.4///RP11-164P12.5
1.2524414	GOLPH3L

1.255775	0161676///RP11-164P12.5
1.2578521	PEAR1
1.2588506	PPA2
1.2588892	SFMBT2
1.2611866	LETMD1
1.2613049	SMAD1
1.2630625	LOC728730
1.2656536	RAB3IP
1.2671242	
1.2721963	KLHL28
1.2753115	LOC100129518///SOD2
1.2757258	LOC344887
1.2785606	MBNL2
1.2829647	
1.283967	ZBTB21
1.2871509	RAB30
1.2912979	GABARAPL1
1.2921038	C6orf48
1.293664	EAF2
1.2944593	TRIM68
1.2950349	MEF2C
1.295044	JPX
1.2992783	CEBPG
1.2997866	SMIM14
1.3011274	IFI16
1.3036551	NLRC5
1.3053656	BC3///MIR3190///MIR3191
1.3071795	ZBTB41
1.3073263	ARMCX3
1.3080578	UHRF1BP1
1.3084993	FBXL14
1.3109837	IFIT3
1.3154817	SEC22A
1.3162766	GADD45B
1.3189521	ACVR2A
1.3194389	ICAM1
1.3215957	TRIM2
1.3276181	ZNF616
1.3281856	3501///ZNF658///ZNF658B
1.3285704	OSBPL6
1.3312664	AARS
1.3320312	SLC33A1
1.3338628	
1.3354197	NOD2
1.3370008	GBP1
1.3370903	PREPL

1.3374739	ABARAPL1///GABARAPL3
1.3391652	RGS16
1.3414111	FSD1L
1.3427777	
1.3439322	OTUD7B
1.3458271	TNF
1.3478174	GADD45B
1.3530841	MRGPRX3
1.354104	GABBR1///UBD
1.3580375	BTN2A2
1.3581333	NFIL3
1.3612127	SLC10A7
1.3653517	LINC00888
1.3667784	IFIT3
1.3696027	PPM1H
1.370533	28///SNORD29///SNORD31
1.371767	LOC101060511///PRKAB2
1.3752046	
1.376656	ZHX2
1.3770614	XAF1
1.3774018	DNAJC27
1.3814168	ZNF627
1.3845873	GIMAP2
1.3907228	COX19
1.3912778	CLK4
1.3925023	TNF3///C1QTNF3-AMACR
1.3986883	CBX4
1.3992887	FAM46A
1.3993602	ZC3H6
1.4007769	XAF1
1.4059296	PPP1R15A
1.4069581	TRIM22
1.4085021	ZNF599
1.4089589	SLC7A11
1.4105597	TNFRSF9
1.4110737	IFI16
1.4133863	CCDC15
1.4142141	ICAM1
1.4170966	TNF3///C1QTNF3-AMACR
1.4198513	IFIT2
1.420609	IFIH1
1.4209366	TIFA
1.4217424	NMNAT1
1.421948	CLU
1.4228001	TMEM50B
1.423626	RAB3IP

1.4270556	CTHRC1
1.432282	ZMYM5
1.4343948	CCL5
1.435525	FAM134B
1.4372826	DHRS2
1.437614	GEM
1.4409609	KRCC1
1.441185	ADPRM
1.4411993	ZBTB8A
1.4424844	RFESD
1.4472303	RGS5
1.4516525	IFI16
1.4516649	CNTRL
1.4546056	TMEM52B
1.4549618	SNHG8///SNORA24
1.4555206	EIF2AK3
1.4567137	GADD45B
1.4573035	ZNRD1-AS1
1.4584284	OSBPL1A
1.4621654	RASSF4
1.4627824	CPEB3
1.4654455	ZMYM5
1.4670577	0506029///LOC100506051
1.4682035	DAPP1
1.4711533	SLC2A13
1.4713464	FAHD2CP
1.4764681	SMIM7
1.4765453	
1.4778261	LOC100506548///RPL37
1.4839296	OSBPL6
1.4842982	LYRM9
1.4872398	RIPK2
1.4900794	SUSD4
1.4902773	SLC30A1
1.4934545	0131122///RP11-499E18.1
1.494719	LOC374443
1.4956341	SERPINA3
1.4986701	TRIM2
1.5018883	IL36G
1.5023093	ZMYM5
1.5094261	ID3
1.513135	PPAP2B
1.5134151	LOC100287497
1.525137	C12orf60
1.5289869	PTGFR
1.5306525	STARD5

1.53437	RBM43
1.5383215	SOCS1
1.5425606	RIPK2
1.5428743	PPARGC1A
1.5437446	C1orf115
1.544538	SEC24D
1.544879	BHLHE40
1.5501361	
1.5533233	
1.572505	LOC100506342
1.574873	PPAP2B
1.578906	LOC100507316
1.5800443	UNC13C
1.5812471	CAPN14
1.5828481	THAP9-AS1
1.5889063	RTP4
1.5961456	GORAB
1.5997915	C6orf132
1.6011281	UNC13C
1.6022305	/CCL3L3///LOC101060267
1.6026425	TLR3
1.6059513	A///SNORA44///SNORA61
1.6062994	/SNORD45B///SNORD45C
1.6084087	TDO2
1.6144438	DAPP1
1.6183052	NCOA7
1.6203661	
1.6211691	VSNL1
1.6336608	PIK3R1
1.654758	CIRBP
1.6772923	MAP2K6
1.6833601	ADPRM
1.6907787	LINC00888
1.6923437	CREB5///LOC401317
1.6924148	PPP4R4
1.6949301	AQP11
1.6950431	GBP2
1.6999559	DISP1
1.7176561	FAM129A
1.7194681	APOL6
1.7209425	GPCPD1
1.7314396	SPATA7
1.7352071	SULT1C2
1.7410059	SLC30A1
1.7455282	WSB1
1.7471113	SLC1A4

1.7476993	SYNE1
1.7567048	ZMYM5
1.7586665	TMPRSS3
1.7637291	GNG2
1.7811861	IRF1
1.7815495	PPAP2B
1.7884922	FICD
1.8006096	LRMP
1.8175287	SPOCK3
1.8309498	TRIM2
1.8376927	EFNA1
1.839179	SPOCK3
1.8401318	SGPP2
1.8469563	FRMD4B
1.8519759	XAF1
1.8531384	LOC374443
1.8538132	GDPD1
1.854826	HIST1H2AC
1.8562255	PAG1
1.869535	ENPP5
1.8931184	MAK
1.8942695	RNF144B
1.9150066	FAM129A
1.9214382	TRIB3
1.9306245	PPP4R4
1.940289	ILDR1
1.9478941	PTX3
1.9655437	INHBE
1.9697847	MRPS31P5///THSD1
1.9779966	ANKFN1
1.9789844	FLVCR2
1.9990411	STXBP6
2.0092983	MAP2
2.0109906	IL15
2.017261	APOL6
2.0199242	IRF1
2.0214329	ICAM1
2.0301423	TRIB3
2.0367312	HMGN2P46
2.0483122	CXCL11
2.0580225	PHGDH
2.058587	APOL6
2.0688143	SERPINI1
2.073923	SUSD5
2.075327	ATF3
2.0899315	SLFN5

2.0912085	LNX1
2.0974188	SA///SNORA44///SNORA61
2.1000662	
2.1185007	CCL5
2.1195989	SLC16A14
2.120306	TNFAIP2
2.124343	JDP2
2.1391711	RNF213
2.152903	CCL5
2.1550765	
2.170165	RWDD2A
2.1773663	SYTL2
2.1795511	PMS1
2.1817894	SYTL2
2.207686	MAP3K8
2.2155552	SEPSECS
2.230216	SLC3A2
2.2337976	SLFN5
2.2443085	FGF7///KGFLP1///KGFLP2
2.254799	PSPH
2.2779536	FBXO16
2.2875643	
2.2955198	DNAJB9
2.3013515	UNC5B
2.3334465	FAM189A2
2.3345528	LAMP3
2.3400335	CLEC2D
2.350257	NPL
2.3549447	CNKSR3
2.3676257	PMS1
2.3682375	DNAJB9
2.3694744	PCK2
2.3793561	SEC24D
2.3912592	PTPRO
2.3951921	ERAP1
2.3960524	GDF15
2.4129782	SGPP2
2.4199069	
2.4381533	C5orf56
2.44909	BAMBI
2.4526653	OLR1
2.4672675	ZNF204P
2.474564	IL15
2.4751778	FLJ35024
2.4985805	PSPH
2.5060592	TUBE1

2.578658	DSG3
2.5846696	SESN2
2.58528	SLFN5
2.5905838	HERPUD1
2.6132448	CLGN
2.6149292	SESN2
2.6328049	MT1M
2.7175727	SLFN5
2.7359743	NPL
2.7602415	SLC7A11
2.7647047	GADD45A
2.825283	TNFRSF9
2.8309574	TSC22D3
2.872302	SLC7A11
2.8929892	SLFN5
2.896964	LRRC49
2.9203057	DNAJB9
2.928122	RASGRP1
3.0064156	CXCL11
3.034503	ATF3
3.1600833	CHAC1
3.181509	DDIT3
3.2287016	ASNS
3.2854033	RCAN1
3.4082327	NEURL3
3.44284	CTH
3.5035713	CLEC2D
3.738473	CTH
3.7477784	RCAN1
4.2090034	ALDH1L2
