# Supplementary figures and images for: Regorafenib inhibits epithelial-mesenchymal transition and suppresses cholangiocarcinoma metastasis via YAP1-AREG axis
Source: Cell Death Dis. 2022 Apr 21;13(4):391. doi: 10.1038/s41419-022-04816-7 (PMC9023529; doi:10.1038/s41419-022-04816-7)

Fig 1F

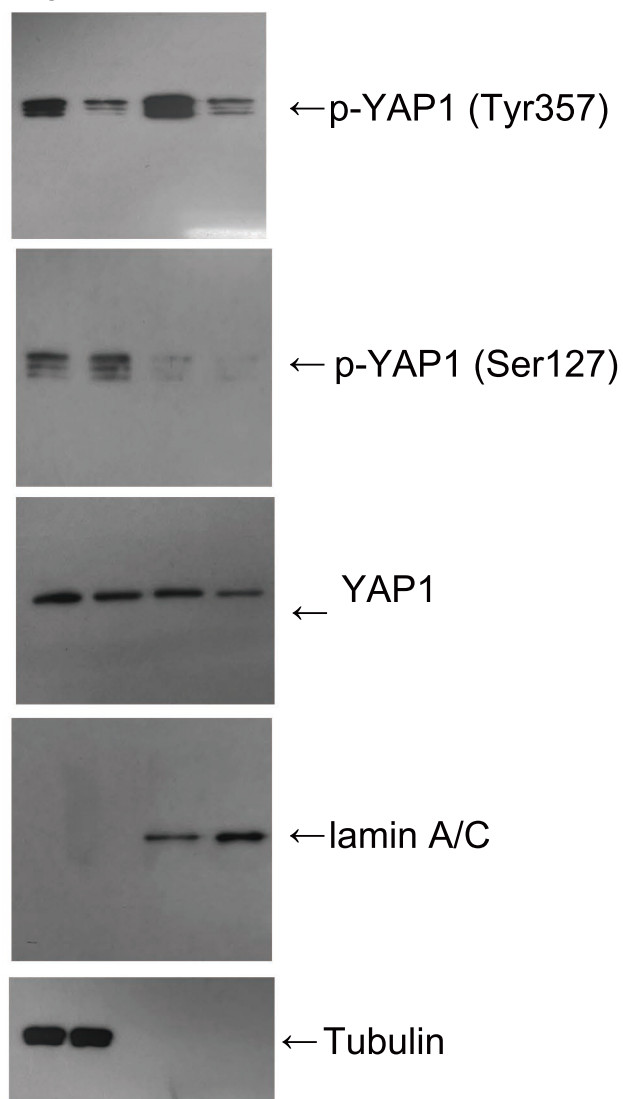

Fig 2C

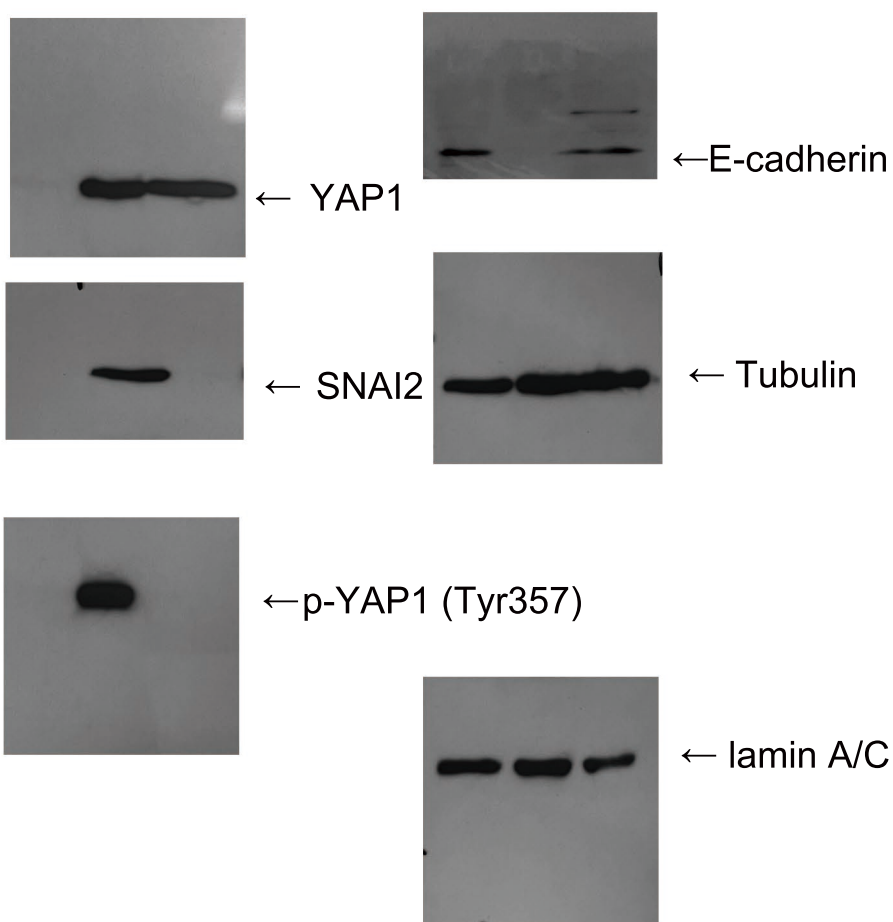

Fig.4C

Fig 2B

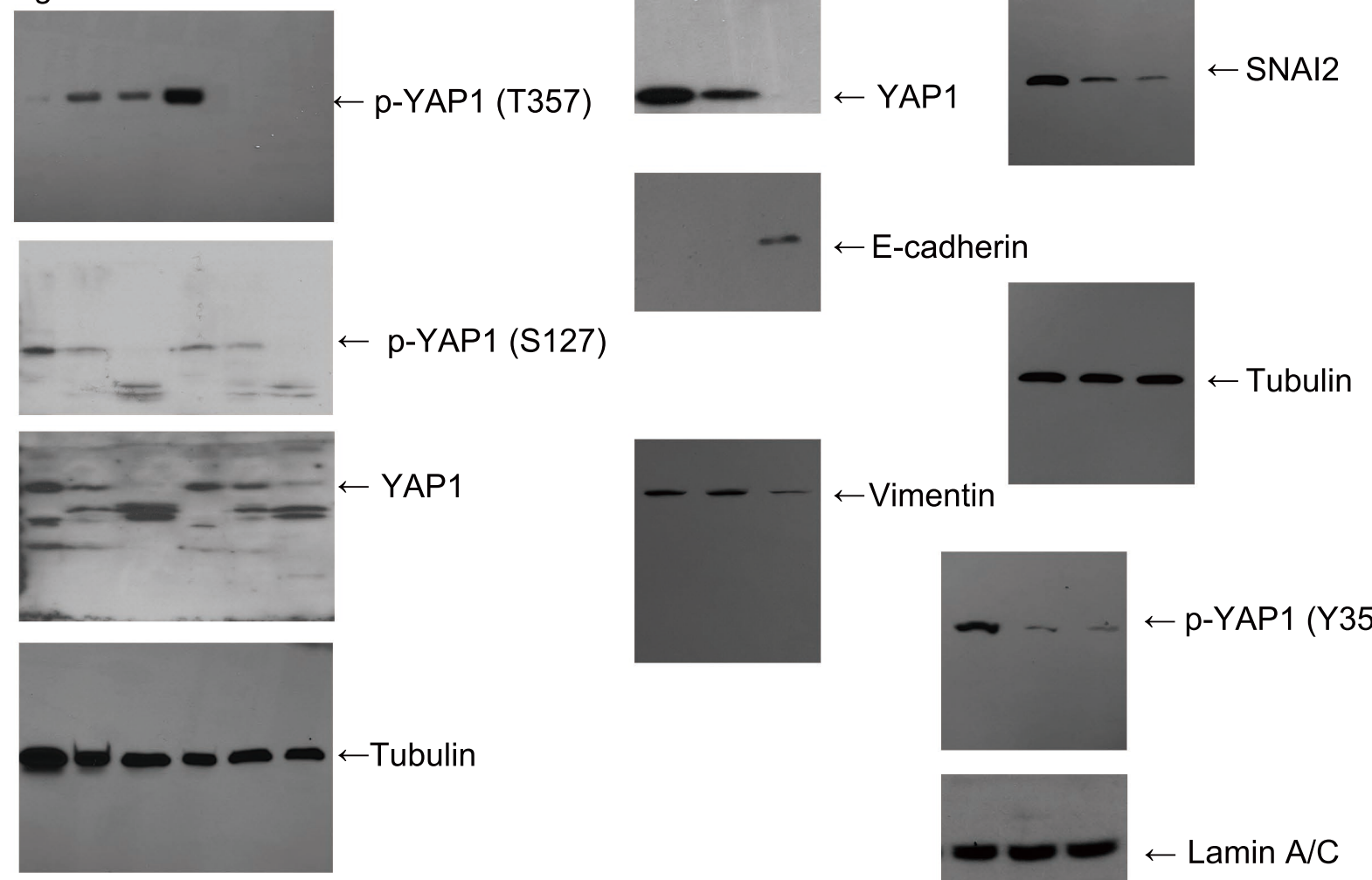

Fig. 4D

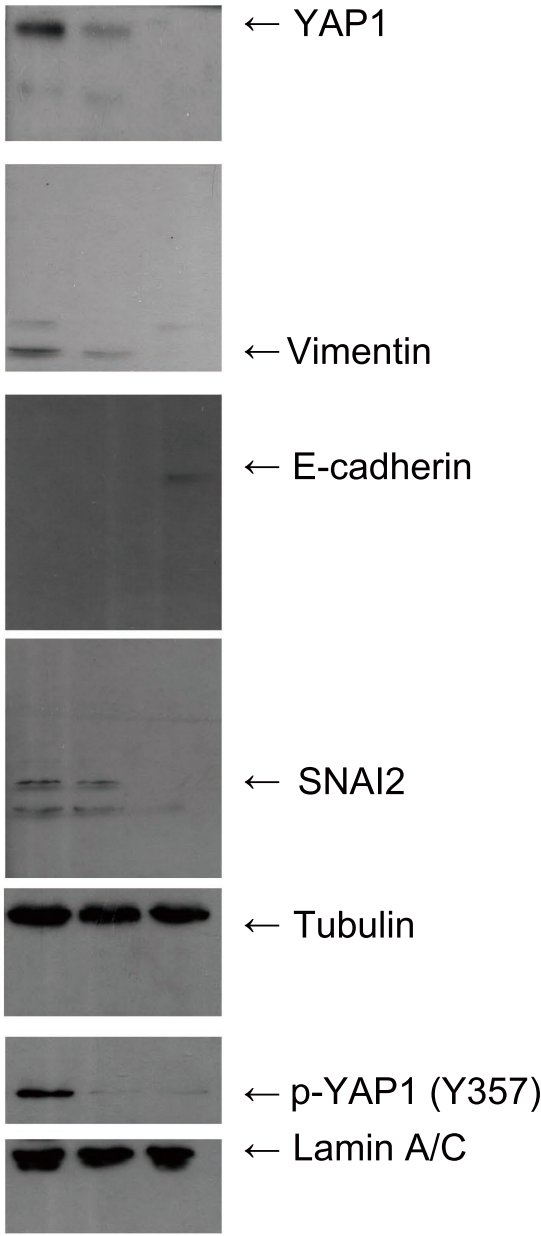

Fig 4E

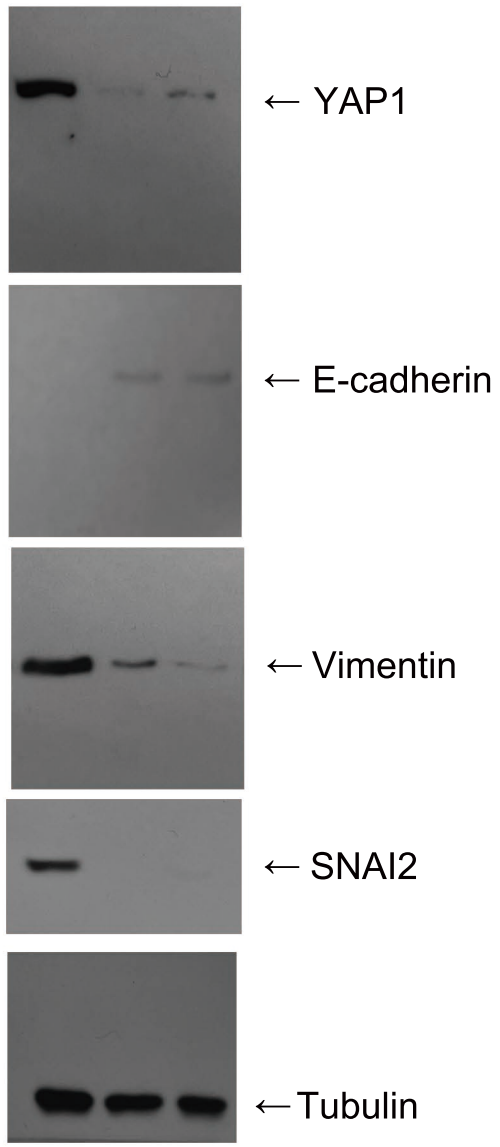

Fig 4G

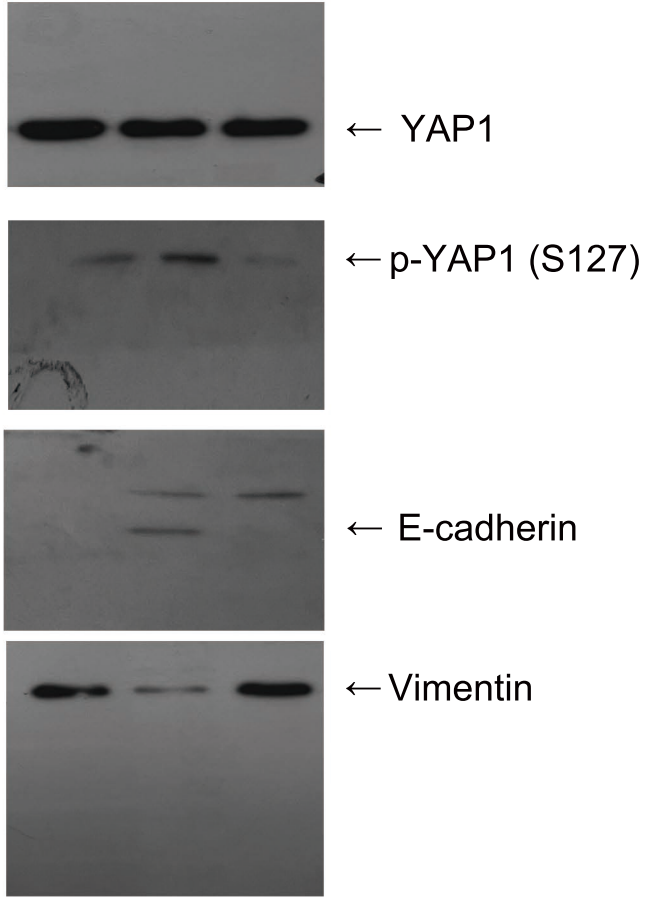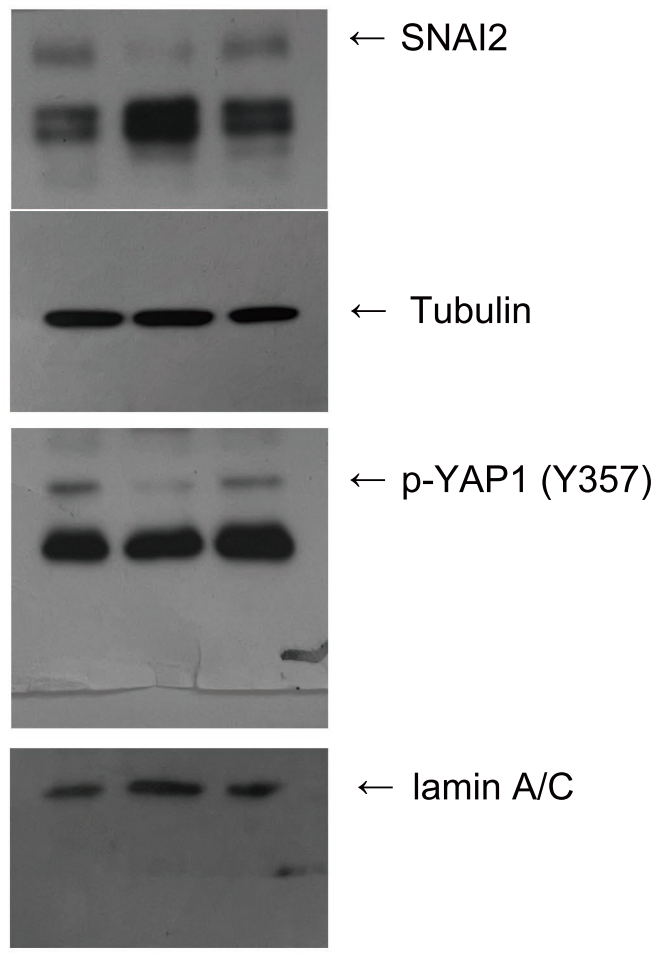

Fig S4

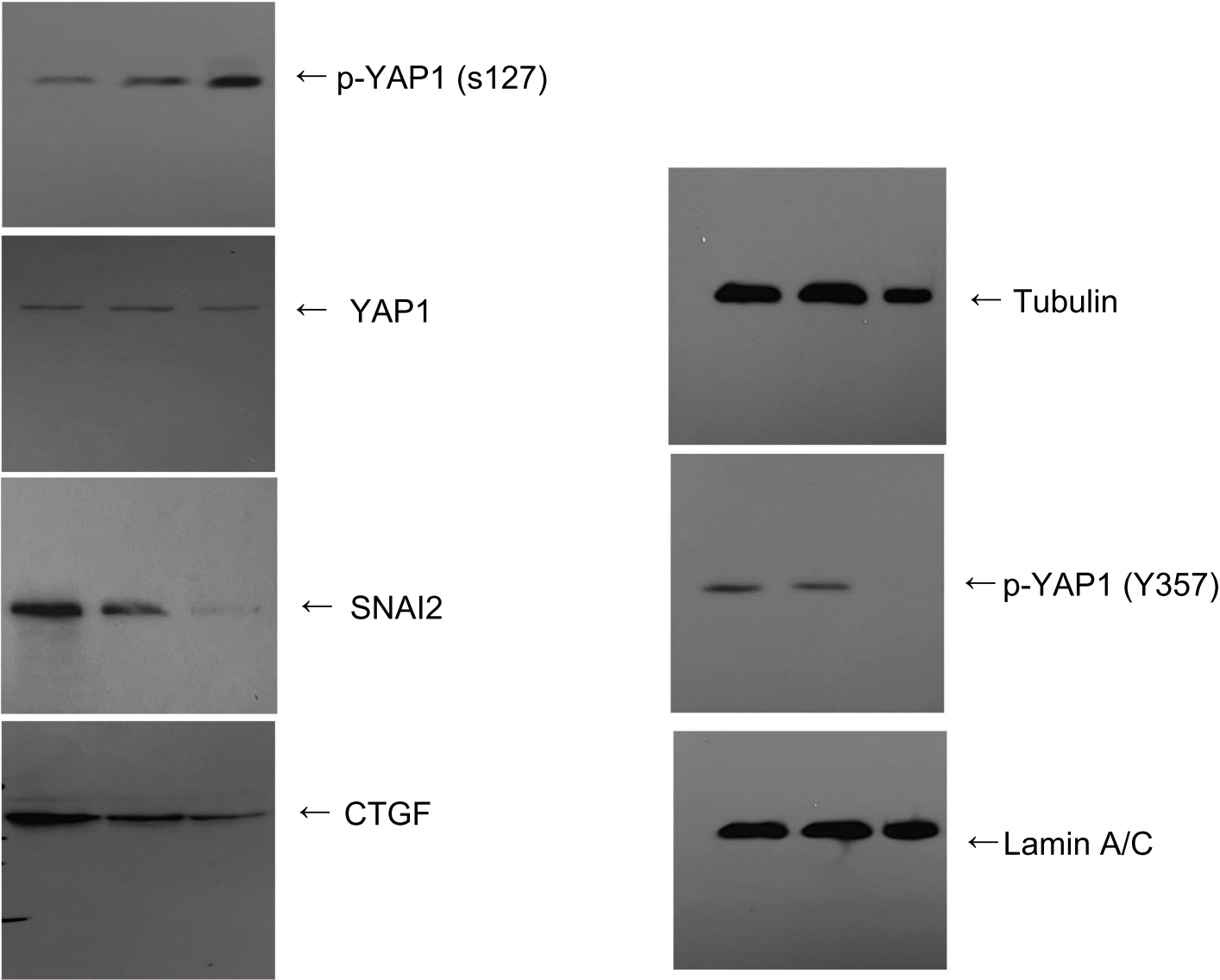

Supplement: Supplementary file 5 — original WB blots [file 41419_2022_4816_MOESM5_ESM.pdf]
